# Oxidative fluorination with Selectfluor: A convenient procedure for preparing hypervalent iodine(V) fluorides

**DOI:** 10.3762/bjoc.20.157

**Published:** 2024-07-29

**Authors:** Samuel M G Dearman, Xiang Li, Yang Li, Kuldip Singh, Alison M Stuart

**Affiliations:** 1 School of Chemistry, University of Leicester, Leicester, LE1 7RH, UK; 2 School of Chemical Engineering, Dalian University of Technology, No. 2 Linggong Road, Dalian, 116024, P. R. China

**Keywords:** fluorination, fluorobenziodoxoles, halogen bonding, hypervalent iodine, Selectfluor

## Abstract

The ability to investigate hypervalent iodine(V) fluorides has been limited primarily by their difficult preparation traditionally using harsh fluorinating reagents such as trifluoromethyl hypofluorite and bromine trifluoride. Here, we report a mild and efficient route using Selectfluor to deliver hypervalent iodine(V) fluorides in good isolated yields (72–90%). Stability studies revealed that bicyclic difluoro(aryl)-λ^5^-iodane **6** was much more stable in acetonitrile-*d*_3_ than in chloroform-*d*_1_, presumably due to acetonitrile coordinating to the iodine(V) centre and stabilising it via halogen bonding.

## Introduction

**Scheme 1:**
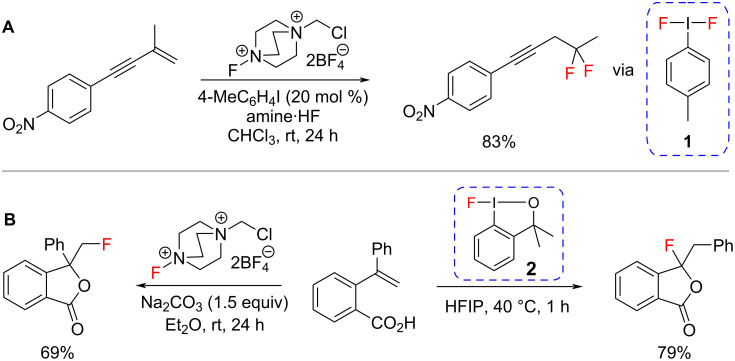
Examples of fluorination using hypervalent iodine(III) reagents **1** and **2**.

An important strategy in the drug discovery process is the incorporation of fluorine into biologically active molecules because fluorine can improve bioactivity and pharmacokinetic properties [1]. Consequently, 22% of all small-molecule drugs contain at least one fluorine atom [2]. Hypervalent iodine(III) fluorides, such as difluoroiodotoluene **1** and fluoroiodane **2**, have been key to the development of numerous, new synthetic procedures for C–F bond formation over the last decade. Since difluoroiodotoluene **1** has low chemical stability and is highly hygroscopic, it is often prepared in situ and Gilmour [3–8] has reported a range of fluorination protocols utilising hypervalent iodine(I/III) catalysis (Scheme 1A). Lennox has also demonstrated that **1** can be generated cleanly by electrochemical oxidation [9–10]. In an alternative approach, we reported the first application of using fluoroiodane **2** as a fluorinating reagent in 2013 [11]. The chelate sidearm makes **2** an air-stable, easy-to-handle solid with excellent fluorinating ability and it often exhibits different reactivity to that observed with fluoroaza reagents such as Selectfluor (Scheme 1B) [12–20].

**Scheme 2:**
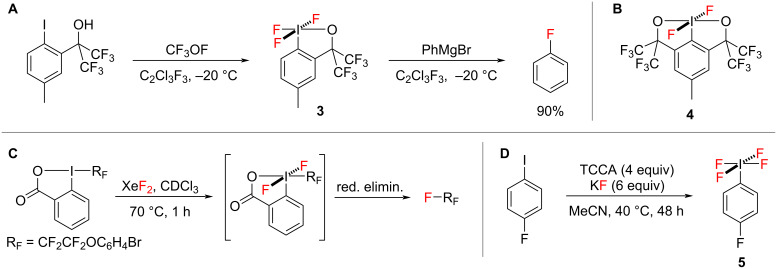
Preparations and reactions of hypervalent iodine(V) fluorides.

In contrast to the chemistry of hypervalent iodine(III) reagents, very little is known about hypervalent iodine(V) fluorides. One problem that has blocked research into these compounds has been the lack of synthetic procedures to access them easily because they normally require harsh fluorinating reagents. The synthesis of hypervalent iodine(V) fluoride **3** was reported by Amey and Martin in 1979 using the highly toxic gas, trifluoromethyl hypofluorite (Scheme 2A), and they later prepared bicyclic hypervalent iodine(V) fluoride **4** using bromine trifluoride (Scheme 2B) [21–22]. They also showed that hypervalent iodine(V) fluoride **3** fluorinated phenylmagnesium bromide in Freon-113 to form fluorobenzene in 90% yield (Scheme 2A) and so, it is very surprising that this reagent has not been investigated further. Since then, Gruber [23] reacted a perfluorinated iodine(III) compound with XeF_2_ and postulated the formation of a (perfluoroalkyl)iodine(V) difluoride intermediate which underwent a reductive elimination to afford perfluorinated products (Scheme 2C). In 2019 Togni reported a safer route to a range of acyclic iodine(V) fluorides such as **5** (Scheme 2D) using large excesses of trichloroisocyanuric acid (TCCA) and potassium fluoride [24]. The iodine(V) fluorides were formed in good spectroscopic yields (79–94%), but only one product, tetrafluoro(4-fluorophenyl)-λ^5^-iodane **5**, was isolated from the reaction mixture by performing multiple extractions into hexane under a nitrogen atmosphere in a glovebox. A similar synthetic approach to acyclic iodine(V) fluorides was developed more recently by Ismalaj and co-workers by reacting iodoarenes with 6 equivalents of KF and ex situ-generated chlorine gas within a two-chamber reactor setup, but again the iodine(V) fluorides were not isolated [25].

We became interested in developing a convenient procedure to access these intriguing reagents and to investigate their ability to fluorinate aryl Grignard reagents. In this paper, we report a straightforward route to hypervalent iodine(V) fluorides by reacting iodine(III) precursors with commercially available Selectfluor. The method avoids large excesses of reagents and pure iodine(V) fluorides are isolated after a simple work-up.

## Results and Discussion

### Preparation of bicyclic difluoro(aryl)-λ^5^-iodanes

**Figure 1:**
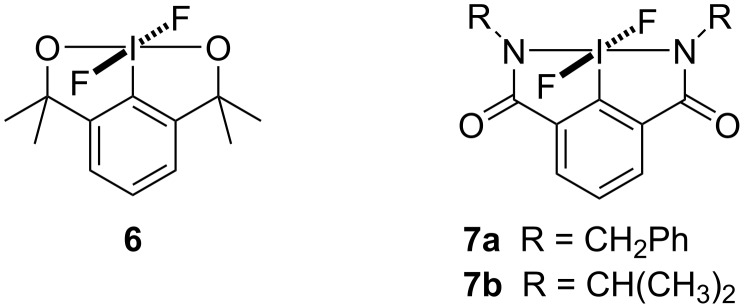
Bicyclic difluoro(aryl)-λ^5^-iodanes.

Two different types of bicyclic difluoro(aryl)-λ^5^-iodanes were designed originally because of the stabilisation afforded from two five-membered rings (Figure 1). We started our investigation with bicyclic difluoro(aryl)-λ^5^-iodane **6**, building on the hypervalent iodine core skeleton used in fluoroiodane **2**, with an additional 5-membered ring to stabilise the iodine(V) centre. Both sidearms were also changed to amides because the NR group is a point of diversity which could be used to modulate the sterics and electronics of these novel hypervalent iodine(V) compounds.

Initially, we applied Togni’s oxidative fluorination protocol to iodine(I) precursor **8** (Table 1). Reacting **8** with 4 equivalents of trichloroisocyanuric acid (TCCA) and 6 equivalents of potassium fluoride in dry acetonitrile at 40 °C for 48 hours formed difluoroiodane **6** in a 90% spectroscopic yield (Table 1, entry 1). An iodosyl decomposition product **9** was also formed during the work-up procedure in air. When the reaction time was shortened to 24 hours, a complex reaction mixture was obtained (Table 1, entry 2). Reducing the amount of TCCA to 3 equivalents (Table 1, entry 3) delivered difluoroiodane **6** in the same 90% yield. Finally, we performed the reaction and work-up under inert conditions and an excellent 99% yield of difluoroiodane **6** was achieved. The main issue with this procedure, however, was that we could not extract the pure difluoroiodane **6** into hexane and separate it from the large excesses of TCCA. Selectfluor was therefore explored as an oxidative fluorinating reagent (Table 1, entry 5). When **8** was reacted with 4 equivalents of freeze-dried Selectfluor in dry acetonitrile at 40 °C for 48 hours, difluoroiodane **6** was formed in 85% spectroscopic yield. However, the iodosyl decomposition product **9** was also produced in 15% spectroscopic yield, despite working the reaction up under inert conditions.

**Table 1:** Oxidative fluorination of iodine(I) substrate **8**.

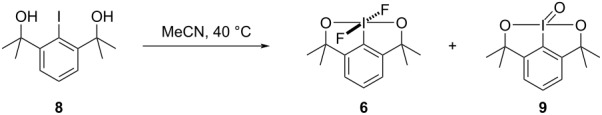				

Entry	Reaction conditions	Time (h)	Yield^a^	

			**6** (%)	**9** (%)

1	TCCA (4 equiv), KF (6 equiv)	48	90	10
2	TCCA (4 equiv), KF (6 equiv)	24	complex mixture	
3	TCCA (3 equiv), KF (6 equiv)	48	90	10
4	TCCA (3 equiv), KF (6 equiv), work-up under N_2_	48	99	1
5	Selectfluor (4 equiv), work-up under N_2_	48	85 (56)	15

^a^Yield calculated by ^1^H NMR spectroscopy, isolated yield shown in parenthesis.

Consequently, we decided to investigate the oxidative fluorination of iodine(III) substrate **10** with Selectfluor (Table 2). We were delighted that difluoroiodane **6** was formed in 93% spectroscopic yield and the iodosyl byproduct **9** was formed in a much lower 7% spectroscopic yield, when **10** was reacted with a large excess of Selectfluor (5.1 equivalents) in dry acetonitrile at 40 °C for 48 hours (Table 2, entry 1). More importantly, difluoroiodane **6** was isolated successfully in an excellent 91% yield by a simple extraction into dry dichloromethane providing an efficient separation from the excess Selectfluor and its byproduct. Reducing the amount of Selectfluor to 2.5 equivalents and the reaction time to 24 hours (Table 2, entry 2) resulted in a similar high yield of difluoroiodane **6**. The reaction also proceeded well at either room temperature for 24 hours (Table 2, entry 3) or at 40 °C for 6 hours (Table 2, entry 4). Finally, reducing the amount of Selectfluor to 1.5 equivalents led to an excellent 90% isolated yield and the conclusion that Selectfluor delivered one electrophilic fluorine (from the N–F) and one nucleophilic fluoride (from the tetrafluoroborate, BF_4_^−^) to form difluoroiodane **6**.

**Table 2:** Oxidative fluorination with Selectfluor^a^.

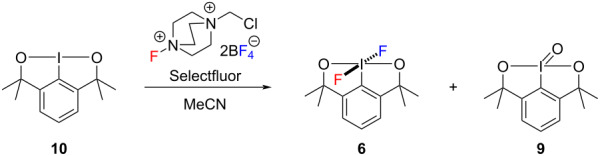						

Entry	Selectfluor (equiv)	Temp. (^°^C)	Time (h)	Conversion (%)	Yield^b^	

					**6** (%)	**9** (%)

1	5.1	40	48	100	93 (91)	7
2	2.5	40	24	100	98 (80)	2
3	2.5	25	24	97	98 (58)	2
4	2.5	40	6	99	95 (55)	5
5	1.5	40	24	99	96 (90)	4

^a^All reactions performed in dry acetonitrile with freeze-dried Selectfluor under nitrogen and work-ups performed in dry solvents under nitrogen; ^b^Yield calculated by ^1^H NMR spectroscopy, isolated yield shown in parenthesis.

The formation of difluoroiodane **6** was identified by a singlet at −23.0 ppm in the ^19^F NMR spectrum. As expected, the aromatic signals in the ^1^H NMR spectrum shifted downfield from a doublet at 7.21 ppm and a triplet at 7.56 ppm for iodine(III) substrate **10** to 7.71 ppm and 7.96 ppm, respectively, for iodine(V) product **6**. Similarly, the ^13^C NMR spectrum showed a major downfield shift for the aromatic carbon attached to iodine from a chemical shift of 105.3 ppm in iodine(III) substrate **10** to 132.4 ppm for difluoroiodane(V) product **6**.

**Scheme 3:**
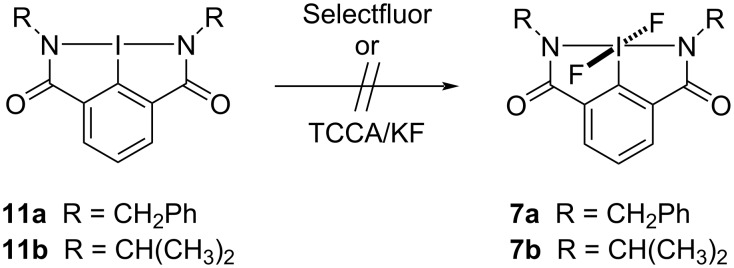
Attempted oxidative fluorination of hypervalent iodine(III) amides.

Since Selectfluor was shown to be the best reagent for preparing bicyclic iodine(V) difluoride **6**, this route was first investigated for the oxidative fluorinations of hypervalent iodine(III) amides **11a** and **11b** (Scheme 3). Unfortunately, these reactions did not work and difluoro(aryl)-λ^5^-iodanes **7a** and **7b** were not produced. Togni’s protocol using TCCA (4 equivalents) and KF (6 equivalents) was then applied to both bicyclic iodine(III) amides **11a** and **11b**, but these reactions also failed to form either difluoroiodane **7a** or **7b**.

Following the successful preparation and isolation of difluoroiodane **6**, we investigated its ability to fluorinate PhMgBr as reported with iodine(V) fluoride **3** by Amey and Martin [21]. Difluoroiodane **6** was first reacted with phenylmagnesium chloride in dry toluene at 0 °C, but fluorobenzene was not formed under these reaction conditions. The reaction was then repeated using phenylmagnesium bromide, but disappointingly, no fluorination was observed. The disparity in reactivity between difluoroiodane **6** and trifluoroiodane **3** towards aryl Grignard reagents could be attributed to the different relationships between the fluorine ligands on the iodine(V) centre. In difluoroiodane **6** the fluorine ligands are restricted to a *trans*-configuration because of the bicyclic carbon skeleton. Trifluoroiodane **3**, on the other hand, has both *trans*- and *cis*-configurations of the fluorine ligands which could play a key role in the reductive elimination step in the fluorination of phenylmagnesium bromide. Trifluoroiodane **3** also contains two trifluoromethyl groups in the sidearm which could alter the electronic effects significantly. We therefore decided to prepare a small series of monocyclic trifluoro(aryl)-λ^5^-iodanes, where the sidearm substituents were changed stepwise from methyl to trifluoromethyl groups, so that we also formed an analogue of Amey and Martin’s monocyclic trifluoroiodane **3**.

### Preparation of monocyclic trifluoro(aryl)-λ^5^-iodanes

**Scheme 4:**
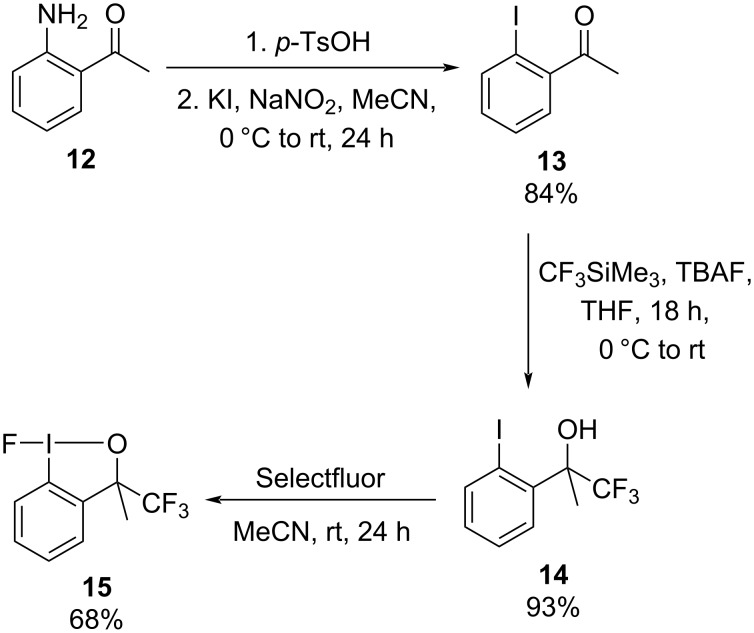
Synthesis of methyl(trifluoromethyl)fluoroiodane **15**.

Our investigation into the synthesis of monocyclic trifluoro(aryl)-λ^5^-iodanes began with the preparation of the key iodine(III) precursors for our oxidative fluorination protocol. Fluoroiodane **2** was already available in our laboratory [11] and the three-step synthesis of methyl(trifluoromethyl)fluoroiodane **15** is shown in Scheme 4. The first step was a diazotisation of 2’-aminoacetophenone **12** to form 2’-iodoacetophenone **13**, which was then reacted with Ruppert’s reagent (CF_3_SiMe_3_) to afford iodoalcohol **14** in 93% yield. In the final step iodoalcohol **14** underwent an oxidative fluorination with Selectfluor at room temperature to deliver methyl(trifluoromethyl)fluoroiodane **15** in a good 68% yield after recrystallisation from toluene.

**Scheme 5:**
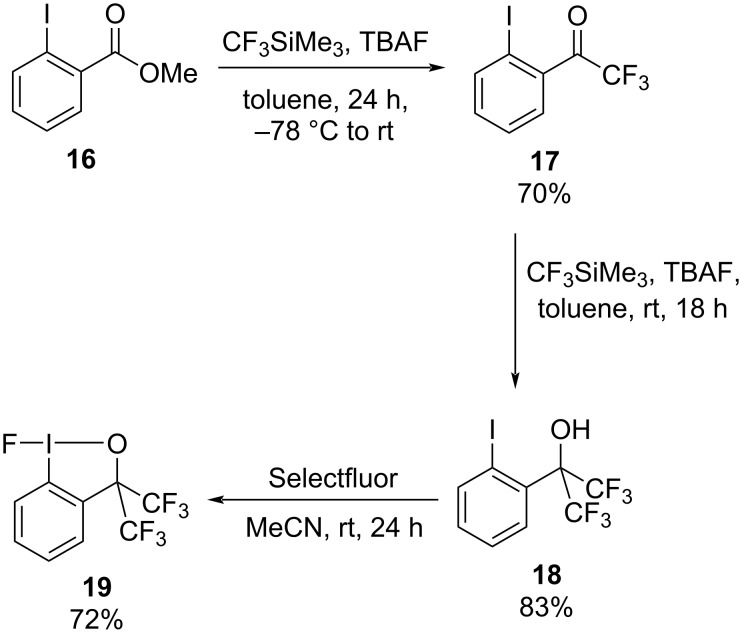
Synthesis of bis(trifluoromethyl)fluoroiodane **19**.

Although bis(trifluoromethyl)fluoroiodane **19** has been reported before [26–28], we developed a new synthetic route which is shown in Scheme 5. In the first step trifluoromethylketone **17** was prepared by a nucleophilic acyl substitution of methyl 2-iodobenzoate **16** with Ruppert’s reagent. Ketone **17** was then reacted with an excess of Ruppert’s reagent and TBAF in order to form bis(trifluoromethyl)iodoalcohol **18**, which was treated with Selectfluor in the final step to deliver bis(trifluoromethyl)fluoroiodane **19** in 72% yield.

A small series of trifluoro(aryl)-λ^5^-iodanes were successfully prepared and isolated in good yields (Table 3). Dimethyltrifluoroiodane **20** was readily formed under mild reaction conditions using 2.5 equivalents of Selectfluor at 40 °C for 24 hours. However, the introduction of trifluoromethyl groups to the sidearm of the iodine(III) fluoroiodanes led to harsher oxidative fluorination conditions being required because the increased electron-withdrawing effect made the fluoroiodane precursors more resistant to oxidation. Consequently, higher temperatures, longer reaction times and more equivalents of Selectfluor were required to prepare trifluoroiodanes **21** and **22**. Trifluoro(aryl)-λ^5^-iodane **22** was also prepared directly from its iodine(I) precursor **18** in 73% isolated yield in a one-pot procedure using 5.5 equivalents of Selectfluor (see Supporting Information File 1).

**Table 3:** Oxidative fluorination of monocyclic fluoroiodanes.

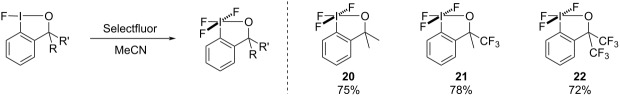						

Entry	R/R’	Product	Selectfluor (equiv)	Temp. (°C)	Time (h)	Yield^a^ (%)

1	CH_3_/CH_3_	**20**	2.5	40	24	75
2	CH_3_/CF_3_	**21**	3.0	60	48	78
3	CF_3_/CF_3_	**22**	3.0	80	72	72

^a^Isolated yield.

**Scheme 6:**
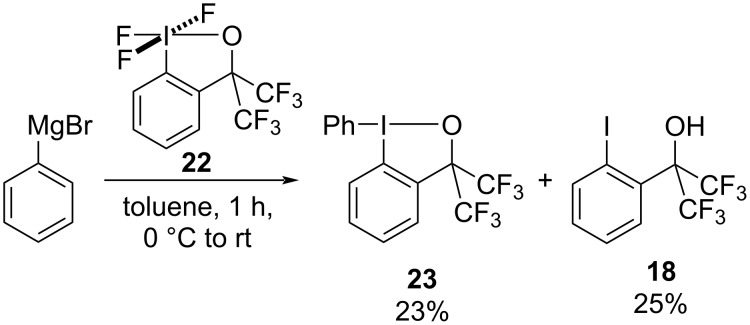
Reaction of phenylmagnesium bromide with bis(trifluoromethyl)trifluoroiodane **22**.

Unfortunately, we were never able to replicate Amey and Martin’s fluorination of phenylmagnesium bromide using bis(trifluoromethyl)trifluoroiodane **22** (Scheme 6). The major products were phenyliodane **23**, presumably a result of ligand exchange and reduction, and iodoalcohol **18**. Different solvents, temperatures and activators were investigated and the results are shown in Table S3 in Supporting Information File 1. Fluorobenzene was only ever observed in trace amounts (1–3 % spectroscopic yield) when BF_3_·OEt_2_ was added to the reaction mixture.

### X-ray crystallography and DFT calculations

**Figure 2:**
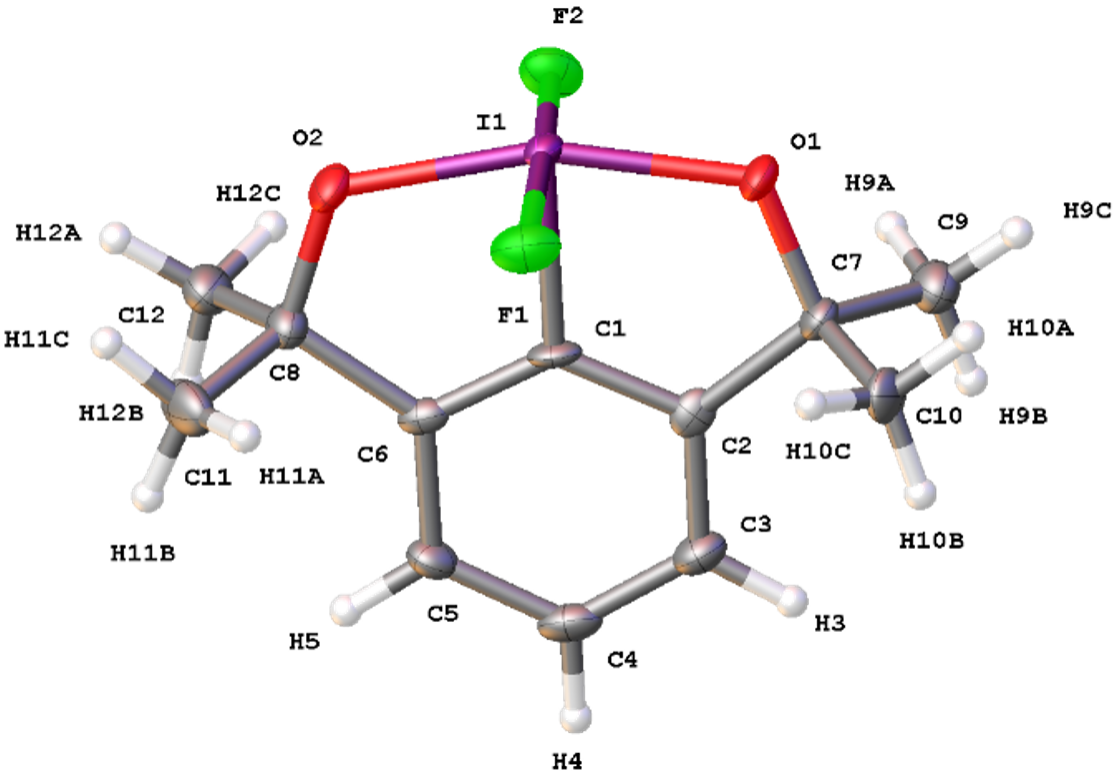
Molecular structure of difluoroiodane **6** showing 50% displacement ellipsoids.

The solid-state structure of difluoroiodane **6** is shown in Figure 2 and displays the expected square pyramidal geometry around the iodine atom, with only minor distortion (*τ*_5_ = 0.191). Since there were two unique molecules in the unit cell, Table 4 compares the average bond lengths and average bond angles of difluoroiodane **6** with trifluoroiodane **20**, which was reported by Togni [24], and fluoroiodane **2** [11]. The I–F bond lengths in difluoroiodane **6** (range from 1.959(4) to 1.990(4) Å) are very similar to those in trifluoroiodane **20** (range from 1.956(4) to 1.984(4) Å), but are shorter than that in fluoroiodane **2** (2.048(3) to 2.058(3) Å) suggesting that fluorine is bound more strongly to the iodine(V) centre than in iodine(III) compounds. There is a similar contraction in the I–O bond lengths when comparing iodine(V) compounds, difluoroiodane **6** (range from 1.977(4) to 1.993(4) Å) and trifluoroiodane **20** (1.924(5) Å), with their respective iodine(III) precursors, **10** (2.096(2) Å) [29] and fluoroiodane **2** (2.029(3) Å). The F–I–F bond angle for difluoroiodane **6** (172.2(2)° to 173.6(2)°) deviates from 180°, but not to the same extent as seen with the aryl-IF_4_ compounds (169.9(1) to 170.4(1)°) or with trifluoroiodane **20** (167.9(2)° to 169.0(2)°) [24]. On the other hand, the O–I–O bond angle for difluoroiodane **6** (161.4(2)°) is much smaller than the F’–I–O bond angles in both trifluoroiodane **20** (167.2(2)° to 168.6(2)°) and fluoroiodane **2** (166.7(2)°), presumably due to the strain caused by the two five-membered rings. Similar to trifluoroiodane **20**, there are two short intermolecular I^…^F contacts of 2.942(4) Å and 2.999(4) Å in the packing diagram of difluoroiodane **6** (see Figure S1 in Supporting Information File 1).

**Table 4:** Selected average bond lengths (Å) and average bond angles (°) with estimated standard deviations (e.s.d.s.) in parenthesis for difluoroiodane **6**, trifluoroiodane **20** and fluoroiodane **2**.

Average bond lengths (Å) and bond angles (°)	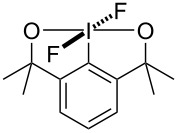 **6**	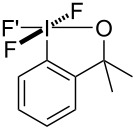 **20**	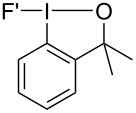 **2**

C–I	2.031(6)	2.072(7)	2.089(5)
I–F	1.975(4)	1.963(4)	–
I–F’	–	1.979(4)	2.053(3)
I–O	1.986(4)	1.924(5)	2.029(3)

F’/O–I–O	161.4(2)	167.9(2)	166.7(2)
F–I–F	172.9(2)	168.5(2)	–
C–I–F	86.7(2)	85.8(3)	–
C–I–F’	–	85.8(3)	86.8(2)
C–I–O	80.7(2)	82.5(2)	80.4(2)


DFT calculations were carried out to gain further insight into the structures of iodine(V) fluorides **21** and **22** whose X-ray structures could not be obtained, and hypothetical iodine(V) amides **7a** and **7b** which could not be made. Comparisons were made with iodine(V) compounds **6** and **20**, as well as with iodine(III) compounds **2** and **19**. Geometry optimisations were performed on all the compounds using Gaussian 16 at wB97xD/cc-pvdz, with a cc-pvdz-PP basis set used for the iodine atom. The calculated bond lengths and bond angles are reported in Table 5 and are in good agreement with the solid state structures. As expected, the calculated atomic charge on iodine was much higher for the iodine(V) fluorides (1.689–1.766) than in the iodine(III) fluorides (0.957–1.009) resulting in shorter I–F and I–O bond lengths. Interestingly, there is a slightly lower charge on iodine in bicyclic iodane **6** (1.689) compared to monocyclic trifluoroiodanes **20**–**22** (1.738–1.766) and the ipso carbon atom is slightly less electronegative (−0.355 vs −0.414) resulting in a less polar, but slightly shorter C–I bond in difluoroiodane **6** (2.031(6) Å) compared to trifluoroiodane **20** (2.072(7) Å). As you go across the series of monocyclic trifluoroiodanes **20** to **22**, the I–O bond length increases slightly due to the electron-withdrawing effect of the trifluoromethyl groups in the sidearms and consequently, the synergistic effect of the 3-centre-4-electron bond causes the I–F’ bond length to decrease slightly. The only major difference between the bicyclic and monocyclic iodine(V) fluorides is the much smaller O–I–O bond angle (161.4(2) °) in difluoroiodane **6** compared to the F’–I–O bond angle (167.9(2) ° in **20**) in the monocyclic iodanes **20**–**22**. In fact, DFT calculations predicted that hypothetical difluoroiodanes **7a** and **7b** containing the amide sidearms would have an even more acute N–I–N bond angle (156.6–156.9 °). Furthermore, the internal chelate NCC bond angle in **7a/b** (111.7°) was calculated to be bigger than the corresponding OCC angle (108.3(5)° to 109.2(5)°) in **6** due to the sp^2^-hybridised carbon in **7a/b** and an sp^3^-hybridised carbon in **6**. This NCC bond angle (111.7°) would certainly increase the angle strain in **7a/b** and this, combined with the acute N–I–N bond angle (156.6–156.9°) caused by these two five-membered rings, could be the reason that we could not prepare these compounds.

**Table 5:** Comparing properties of hypervalent iodine(V) fluorides with hypervalent iodine(III) fluorides **2** and **19**^a^.

	d(C–I) (Å)	d(I–F) (Å)	d(I–F’) (Å)	d(I-O/N) (Å)	q_C_	q_I_	q_F_	q_F‘_	q_O/N_	θ_F–I–F_ (°)	θ_O-I-O/F’_/ θ_N-I-N_ (°)

**6**	2.04	2.00^b^	–	2.03	−0.355	1.689	−0.460	–	−0.561	173.6	161.1
**7a**	2.06	2.00	–	2.12	−0.312	1.668	−0.426	–	−0.609	175.9	156.6
**7b**	2.05	1.99^b^	–	2.12	−0.314	1.667	−0.444	–	−0.591	176.7	156.9

**20**	2.09	1.98^b^	1.99	1.99	−0.419	1.738	−0.453	−0.449	−0.556	171.8	168.8
**21**	2.09	1.98^c^	1.98	2.01	−0.415	1.753	−0.445^d^	−0.439	−0.550	171.7	168.6
**22**	2.10	1.97^b^	1.97	2.03	−0.407	1.766	−0.438	−0.428	−0.540	171.4	168.5

**2**	2.10	–	2.04	2.07	−0.403	0.957	–	−0.490	−0.574	–	167.9
**19**	2.10	–	2.02	2.09	−0.387	1.009	–	−0.469	−0.552	–	167.8


^a^Calculations performed at wB97xD/cc-pvdz, with a cc-pvdz-PP basis set used for the iodine atom. C refers to ipso carbon atom, F and F’ refers to fluorine atom bound to iodine. ^b^Structure is nearly symmetric about F–I–F (mirror plane). ^c^Average between 1.99 and 1.97. ^d^Average between −0.441 and −0.449.

### Stability studies of hypervalent iodine(V) fluorides in solution

**Figure 3:**
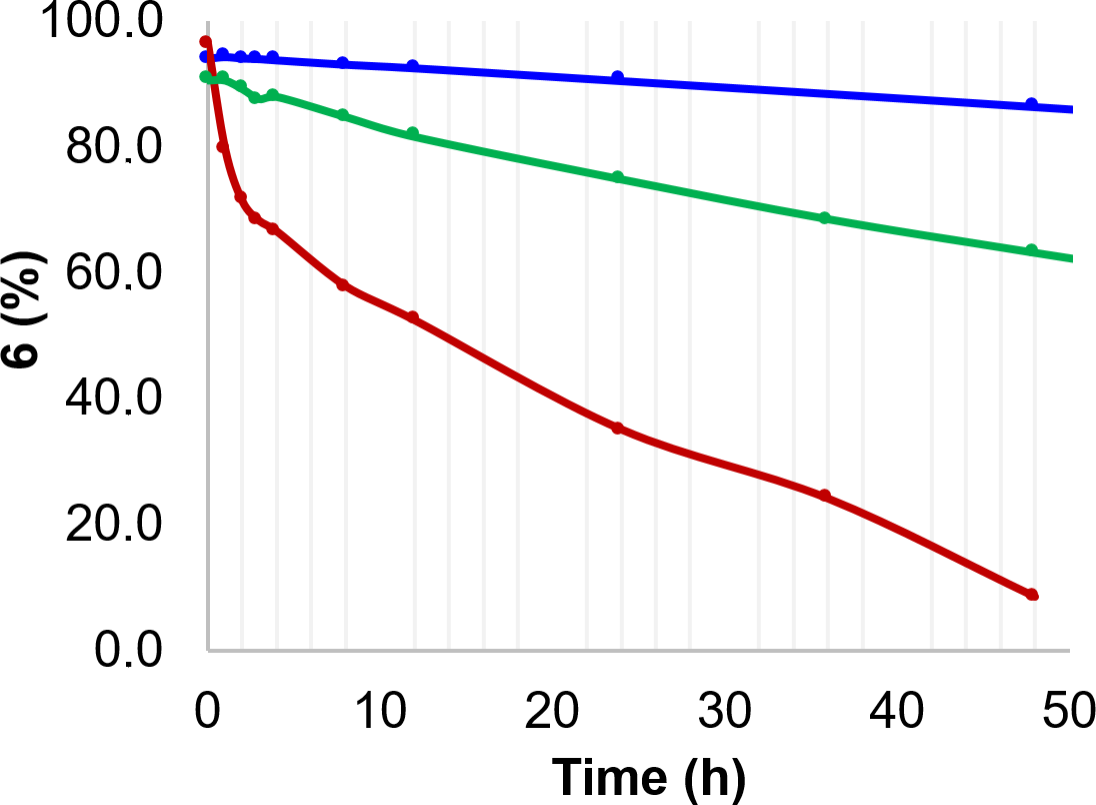
Stability of difluoroiodane **6** in air in dry CD_3_CN (blue line), dry CDCl_3_ with 2.4 equivalents of dry pyridine (green line), and dry CDCl_3_ (red line).

The stability of hypervalent iodine(V) fluorides **6**, **20**, **21** and **22** was studied in dry acetonitrile-*d*_3_ by ^1^H and ^19^F NMR spectroscopy over 7 days under an argon atmosphere. All four hypervalent iodine(V) fluorides were stable for the 7-day period. When the same experiment was repeated in air, iodine(V) fluorides **6**, **21** and **22** decomposed to 55–65% remaining after 7 days presumably due to the moisture in the air, whereas trifluoroiodane **20** was less stable with only 37% remaining. Difluoroiodane **6** was also stable in dry chloroform-*d*_1_ under argon over 7 days**,** but it decomposed completely to iodosyl **9** after just 48 hours in dry chloroform-*d*_1_ in air (red line in Figure 3). The difference in the stability of difluoroiodane **6** in CDCl_3_ and CD_3_CN was attributed to the ability of acetonitrile to coordinate to the iodine(V) centre. Stabilisation via halogen bonding is well-established in hypervalent iodine(III) compounds and Dutton showed that pyridine formed a weak complex with dichloroiodobenzene via halogen bonding [30–32]. We therefore added dry pyridine (2.4 equivalents) to difluoroiodane **6** in CDCl_3_ to help stabilise the iodine(V) centre and the rate of decomposition was reduced significantly (green line in Figure 3).

**Figure 4:**
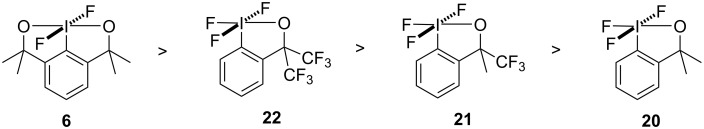
Order of hydrolytic stability for the four hypervalent iodine(V) fluorides.

The hydrolysis of the four hypervalent iodine(V) fluorides was also investigated in acetonitrile-*d*_3_ by adding 5 equivalents of water. All four compounds decomposed to their corresponding iodosyl compounds and the order of stability is shown in Figure 4. Difluoroiodane **6**, containing two 5-membered rings, was the most stable iodine(V) fluoride whereas monocyclic trifluoroiodane **20** was the least stable and decomposed completely within the first minute. As expected, the stability of the monocyclic trifluoroiodanes **21** and **22** was increased by the stepwise incorporation of the trifluoromethyl groups into the sidearm, but trifluoroiodane **22** was less stable than bicyclic difluoro(aryl)-λ^5^-iodane **6**.

## Conclusion

In summary, we have developed a new strategy using Selectfluor for the convenient preparation and isolation of hypervalent iodine(V) fluorides in good yields (72–90%). Unfortunately, none of the iodine(V) fluorides reacted with phenylmagnesium bromide to form fluorobenzene and we were never able to repeat Amey and Martin’s fluorination of phenylmagnesium bromide. A solid-state structure of **6** and DFT calculations on **6** and **20**–**22** gave insights into the geometries of the iodine(V) fluorides compared to the iodine(III) precursors. DFT results also suggested a possible reason for not being able to make iodine(V) amides **7a** and **7b**. An investigation into the hydrolysis of the four hypervalent iodine(V) fluorides revealed that bicyclic difluoro(aryl)-λ^5^-iodane **6** was more stable than monocyclic trifluoro(aryl)-λ^5^-iodanes **20**–**22** due to the incorporation of the second 5-membered ring in the 3-centre-4-electron bond.

## Experimental

Dioxoiodane **10** (0.58 g, 1.8 mmol), Selectfluor (0.97 g, 2.7 mmol) and dry acetonitrile (10 mL) were charged to a dry Schlenk flask under a nitrogen atmosphere. The flask was sealed and heated to 40 °C for 24 hours. After cooling the reaction mixture to room temperature, the solvent was removed in vacuo to afford a crude orange solid. The orange solid was extracted with dry dichloromethane (3 × 5 mL) under a nitrogen atmosphere and the dichloromethane was removed in vacuo to afford difluoroiodane **6** as a pale orange solid (0.58 g, 90%).

## Supporting Information

Crystallographic data (excluding structure factors) for the structures reported in this paper have been deposited with the Cambridge Crystallographic Data Centre and allocated the deposition numbers CCDC: 2351949 and 2351950.

File 1 Experimental procedures, characterisation data, DFT calculations and ^1^H, ^13^C and ^19^F NMR spectra and crystallographic data.

## Data Availability

All data that supports the findings of this study is available in the published article and/or the supporting information to this article.
