# Supplementary material for: Oxidative fluorination with Selectfluor: A convenient procedure for preparing hypervalent iodine(V) fluorides
Source: Beilstein J Org Chem. 2024 Jul 29;20:1785–93. doi: 10.3762/bjoc.20.157 (PMC11301045; doi:10.3762/bjoc.20.157)
Supplement: File 1 — Experimental procedures, characterisation data, DFT calculations and 1H, 13C and 19F NMR spectra and crystallographic data. [file Beilstein_J_Org_Chem-20-1785-s001.pdf]

BEILSTEIN JOURNAL OF ORGANIC CHEMISTRY

Supporting Information

for

Oxidative fluorination with Selectfluor: A convenient procedure for preparing hypervalent iodine(V) fluorides

Samuel M. G. Dearman, Xiang Li, Yang Li, Kuldip Singh and Alison M. Stuart

Beilstein J. Org. Chem. **2024,** *20,* 1785–1793. doi:10.3762/bjoc.20.157

Experimental procedures, characterisation data, DFT calculations and ^{1}H, ^{13}C and ^{19}F NMR spectra and crystallographic data

License and Terms: This is a supporting information file under the terms of the Creative Commons Attribution License (https://creativecommons.org/licenses/by/4.0). Please note that the reuse, redistribution and reproduction in particular requires that the author(s) and source are credited and that individual graphics may be subject to special legal provisions.
The license is subject to the *Beilstein Journal of Organic Chemistry* terms and conditions: (https://www.beilstein-journals.org/bjoc/terms)

Figure S1 Packing diagram of difluoroiodane **6** showing intermolecular C–I···F–I contacts S2
Figure S2 Molecular structure of iodosyl **9** S2
Figure S3 Packing diagram of iodosyl **9** showing short intermolecular I···O contacts S3
Table S1 Selected bond lengths (Å) and bond angles (°) for **6** and **9** S4
Experimental procedures and characterisation data S5
Preparation of hypervalent iodine(III) precursor **10** S6
Table S2 Optimisation of the Grignard reaction to prepare **8** S10
General procedure for oxidative fluorination of **8** (Table 1) S14
General procedure for oxidative fluorination of **10** with Selectfluor (Table 2) S15
Preparation of hypervalent iodine(III) amides **11a** and **11b** S15
Attempted oxidative fluorination of iodine(III) amides **11a** and **11b** S17
Procedure for the reaction of difluoroiodane **6** with PhMgBr S19
Preparation of methyl(trifluoromethyl)fluoroiodane **15** S20
Preparation of bis(trifluoromethyl)fluoroiodane **19** S22
General procedure for oxidative fluorination of monocyclic fluoroiodanes (Table 3) S24
Table S3 Reaction of trifluoroiodane **22** with PhMgBr (Scheme 6) S27
General procedure for monitoring the stability of iodine(V) fluorides in dry solvents S29
Figure S4 Stability of hypervalent iodine(V) fluorides **6**, **20**, **21** and **22** in air S30
General procedure for hydrolysing iodine(V) fluorides S33
Figure S5 Hydrolysis of hypervalent iodine(V) fluorides **6**, **20**, **21** and **22** S34
Structure solution and refinement S34
Table S4 Crystallographic data for difluoroiodane **6** and iodosyl **9** S35
References S36
NMR spectra S37
DFT calculations S71

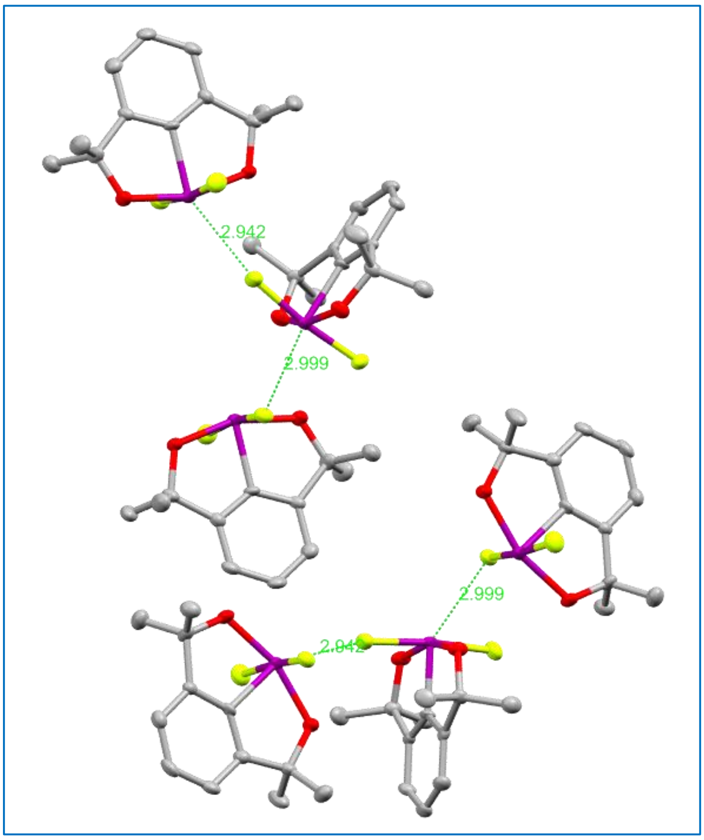

Figure S1: Packing diagram of difluoroiodane **6** showing intermolecular C–I···F–I contacts of 2.942(4) Å and 2.999(4) Å.

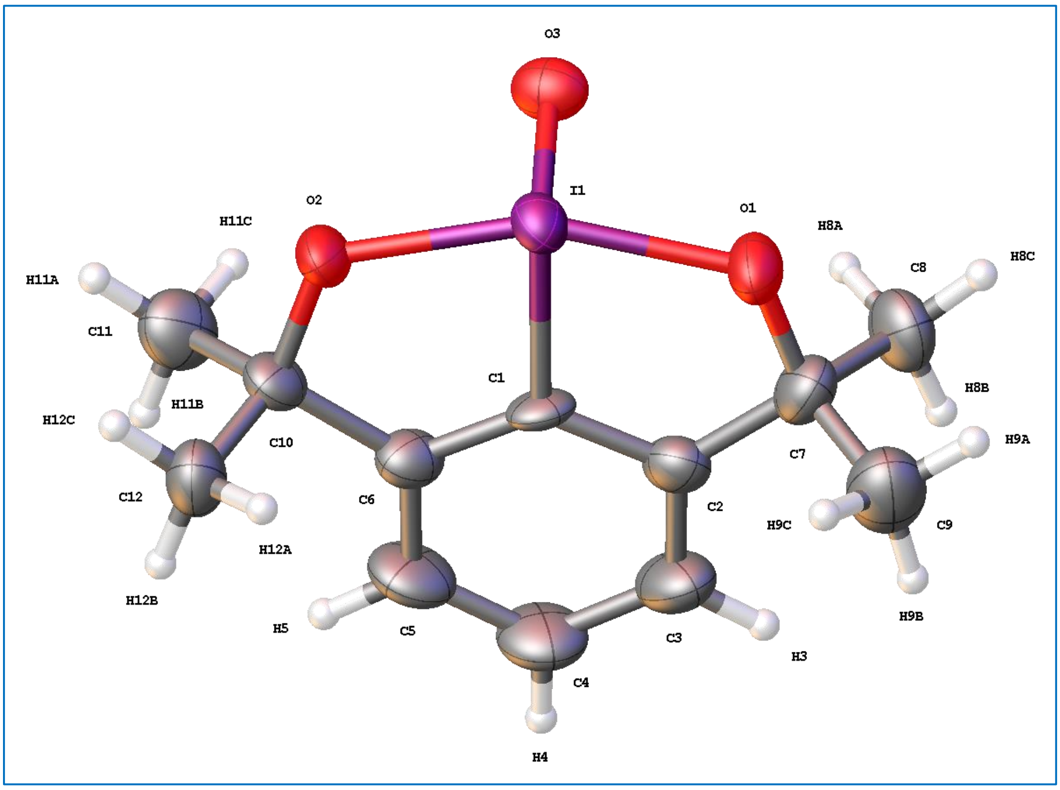

Figure S2: Molecular structure of iodosyl **9** showing 50% thermal displacement ellipsoids

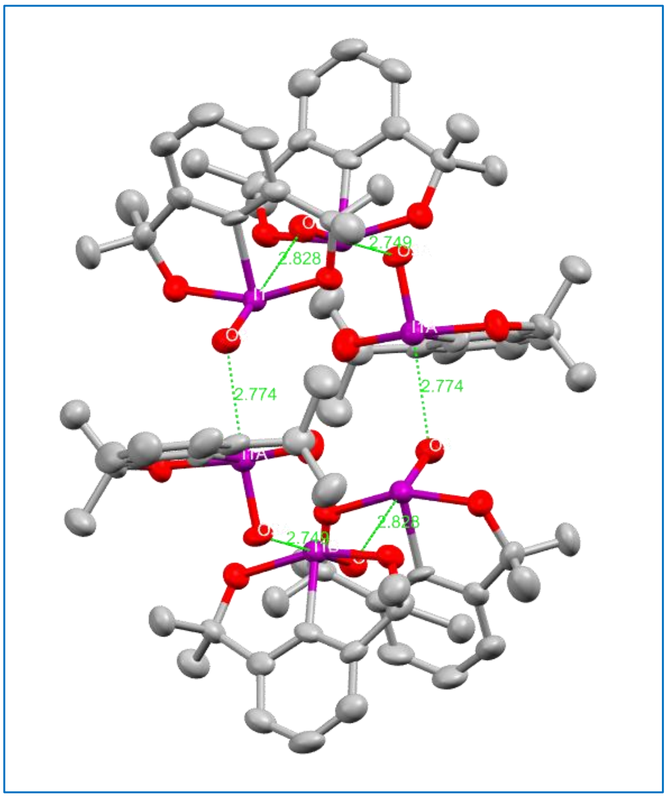

Figure S3: Packing diagram of iodosyl **9** showing intermolecular I···O contacts of 2.749(6) Å, 2.774(6) Å and 2.828(5) Å.

Table S1: Selected bond lengths (Å) and bond angles (°) with estimated standard deviations (e.s.d.s.) in parenthesis for difluoroiodane **6** and iodosyl **9**

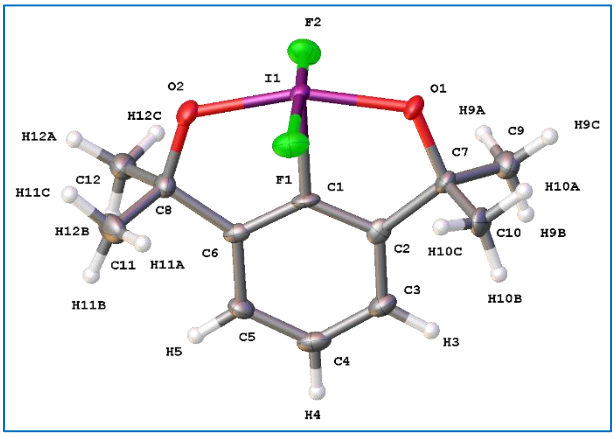

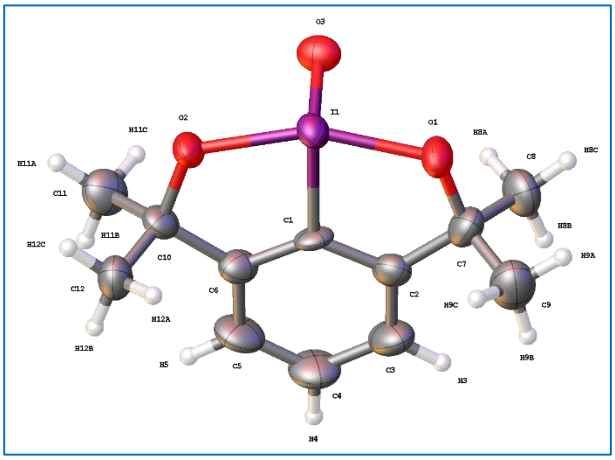

Bond lengths (Å) & bond angles (°)	Difluoroiodane 6[a]		Iodosyl 9[b]		
C(1)-I(1)	2.024(6)	2.038(6)	2.047(8)	2.048(8)	2.041(8)
I(1)-F(1)	1.973(4)	1.976(4)	-	-	-
I(1)-F(2)	1.959(4)	1.990(4)	-	-	-
I(1)-O(1)	1.977(4)	1.989(4)	2.042(6)	2.043(6)	2.051(5)
I(1)-O(2)	1.993(4)	1.983(4)	2.038(5)	2.025(6)	2.057(5)
I(1)-O(3)	-	-	1.808(6)	1.820(5)	1.803(5)
F(1)-I(1)-F(2)	173.58(16)	172.22(16)	-	-	-
O(1)-I(1)-O(2)	161.53(18)	161.34(19)	158.2(2)	158.6(2)	158.0(2)
O(1)-I(1)-O(3)	-	-	94.3(3)	93.9(3)	95.2(2)
O(2)-I(1)-O(3)	-	-	94.4(2)	93.7(3)	92.2(3)
C(1)-I(1)-F(1)	87.1(2)	87.5(2)	-	-	-
C(1)-I(1)-F(2)	87.2(2)	84.8(2)	-	-	-
C(1)-I(1)-O(1)	81.0(2)	80.5(2)	79.1(3)	79.1(3)	79.5(3)
C(1)-I(1)-O(2)	80.5(2)	80.9(2)	79.7(3)	79.6(3)	78.6(3)
C(1)-I(1)-O(3)	-	-	101.0(3)	105.2(3)	103.5(3)

[a] there are two unique molecules in the unit cell; [b] there are three unique molecules in the unit cell.

Experimental procedures and characterisation data

Proton, ^{19}F and ^{13}C nuclear magnetic resonance (NMR) spectra were recorded on either a Bruker AV400 (^{1}H, 400 MHz; ^{13}C, 100 MHz; ^{19}F, 376 MHz), a Bruker AV500 (^{1}H, 500 MHz; ^{13}C, 125 MHz) spectrometer or a Bruker Avance NEO 500 MHz spectrometer (^{1}H, 500 MHz; ^{13}C, 125 MHz, ^{19}F, 471 MHz) with either a 5 mm Prodigy BBO H&F cryoprobe or a 5 mm BBFO smart probe using either deuterated chloroform ($CDCl_3$), or deuterated acetonitrile (CD_3CN). Chemical shifts (δ) were referenced to the residual proton absorption of chloroform-d_1 at δ 7.26 (H) and 77.16 (C) or acetonitrile-d_3 at δ 1.94 (^{1}H) and 118.69 (^{13}C), in parts per million (ppm). Coupling constants are reported in Hertz (Hz). Melting points (m.p.) were determined using a Gallenkamp melting point apparatus (model MFB-595). Atmospheric Solids Analysis Probe (ASAP) mass spectra were recorded on a Xevo QTof mass spectrometer (Waters) and Electrospray (ESI) mass spectra were obtained by LC-MS using a Xevo QTof mass spectrometer (Waters) coupled to an Acquity LC system (Waters) with an Acquity UPLC BEH C18 column (2.1 × 50 mm). X-ray crystallography data were collected on a Bruker Apex SMART 2000 diffractometer using graphite-monochromated Mo-Kα radiation (λ = 0.71073 Å).

All reagents were received either from Sigma-Aldrich, Fisher Scientific or Fluorochem and were used without further purification unless otherwise stated. Dry solvents (MeCN, DCM, toluene, THF, diethyl ether) were obtained dry from the solvent purification system PuresolveTM, and were stored in sealed ampoules over 4 Å molecular sieves under an atmosphere of dry nitrogen. HFIP was dried over 4 Å molecular sieves under an atmosphere of dry nitrogen for 72 hours, then stored over fresh 4 Å molecular sieves under an atmosphere of dry nitrogen. All manipulations were performed under an inert atmosphere of nitrogen using Schlenk line techniques, oven dry glassware and dry solvents unless otherwise stated. Concentrated refers to removal of solvent under reduced pressure on a rotary evaporator. Column chromatography was carried out using Merck Kieselgel 60 (230–400 mesh). Analytical thin-layer chromatography (TLC) was carried out on silica gel F254/366 60 Å plates with visualisation using UV light (254 nm). The hypervalent fluoroiodane reagent **2** was prepared following the literature procedure [11].

Preparation of hypervalent iodine(III) precursor 10 [29]

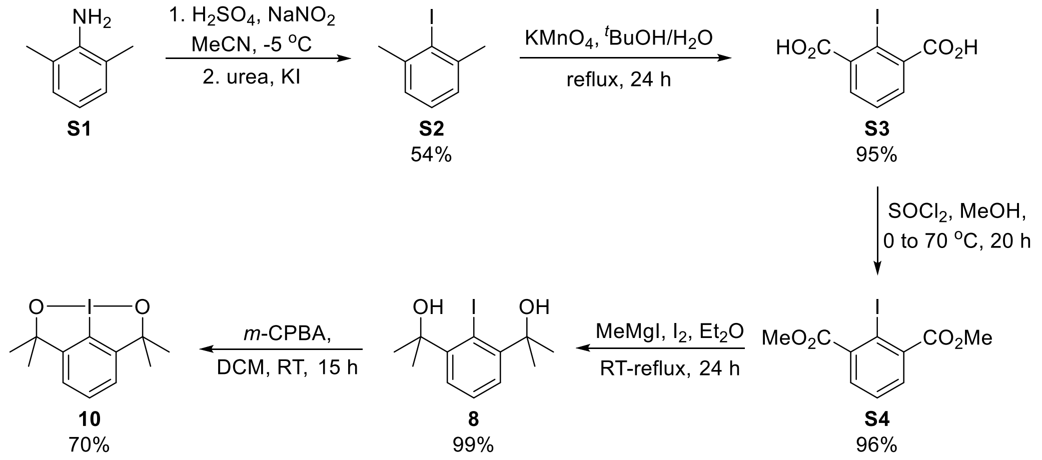

Preparation of 2,6-dimethyliodobenzene (S2) [33]

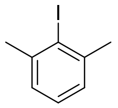

S2

A solution of 2,6-dimethylaniline (**S1**, 5.0 mL, 40.6 mmol) in acetonitrile (35 mL) was added to a 3-neck 500 mL RBF, equipped with condenser, internal thermometer and addition funnel. Conc. H_2SO_4 (4.4 mL, 18.4 M, 81.0 mmol) in H_2O (105 mL) was added dropwise at room temperature, and the solution was cooled to −5 °C, with an ice/acetone bath. Separately, $NaNO_2$ (6.42 g, 93.1 mmol) was dissolved in H_2O (24 mL), and this mixture was then added dropwise to the reaction mixture over 5 minutes. On complete addition, the reaction mixture was warmed to room temperature for 40 minutes. Hereafter, the reaction mixture was cooled to -5 °C, with an ice/acetone bath, and urea (0.52 g, 8.7 mmol) was added. KI (14.58 g, 87.8 mmol) in H_2O (35 mL) was then added slowly over 20 minutes (an exotherm was observed). On complete addition the reaction mixture was stirred for a further 45 minutes at −5 °C, then warmed to room temperature and left stirring for 16 hours. Subsequently, the reaction mixture was quenched with addition of saturated $NaHCO_3$ (50 mL), followed by aqueous $Na_2S_2O_3$ (50 mL, 3.3 M). The solution was then filtered through celite, washed through with ethyl acetate (200 mL) and the filtrate was extracted with EtOAc (3 × 100 mL). The organic extracts were combined, dried ($MgSO_4$), filtered and the solvent was removed in vacuo to afford a crude orange oil (8.81 g). The crude oil was purified by column chromatography (SiO_2, 20wt, 4% ethyl acetate in petroleum ether) to afford pure product **S2** as a pale orange oil (5.10 g, 54%). The characterisation data was in agreement with the literature data [34]. δ_H ($CDCl_3$, 500 MHz) 2.47 (6H, s, CH_3), 7.04 (2H, d, $^3J_{HH}$ = 7.4 Hz, ArH), 7.11 (1H, t, $^3J_{HH}$ = 7.4 Hz, ArH). δ_C ($CDCl_3$, 125 MHz) 29.9 (CH_3), 108.6 (CI), 127.1 (CH), 127.7 (CH), 142.2 (C). m/z (ASAP) 231.9751 ($[M]^+$, C_8H_9I requires 231.9749, 100 %).

Preparation of 2-iodoisophthalic acid (S3) [33]

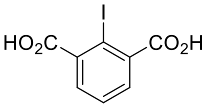

S3

$KMnO_4$ (12.05 g, 76.3 mmol) was added to a solution of 2,6-dimethyliodobenzene (**S2**, 6.12 g, 26.4 mmol) in *t*-BuOH:H_2O (1:1, 360 mL) and the reaction mixture was refluxed for 4 hours. After cooling to 60 °C, a further portion of $KMnO_4$ (12.08 g, 76.5 mmol) was added. The reaction mixture was then refluxed for a further 16 hours (monitored by TLC, Al_2O_3, 5% MeOH in DCM). Hereafter, the reaction mixture was filtered whilst hot, through Celite and under suction. The purple solution was treated with Na_2SO_3 (2.00 g, 15.9 mmol) and the resulting solution was re-filtered through Celite. After cooling the solution, it was concentrated in vacuo to a fifth of the volume. This was subsequently acidified with concentrated hydrochloric acid (ca. 10 mL) and a yellow precipitate was formed. This was filtered to afford 2-iodoisophthalic acid **S3** as a fluffy pale yellow powder (7.29 g, 95%). The characterisation data was in agreement with the literature data [35]. m.p. 237-239 °C (lit., 239-240 °C) [36]. δ_H (d_6-DMSO, 500 MHz) 7.49 (1H, dd, $^3J_{HH}$ = 8.5 & 6.6 Hz, ArH), 7.55 (2H, d, $^3J_{HH}$ = 7.2 Hz, ArH), 13.46 (2H, s, COOH). δ_C (d_6-DMSO, 125 MHz) 91.5 (CI), 128.7 (CH), 130.0 (CH), 141.7 (C), 169.7 (CO). m/z (ESI$^+$) 292.9313 ($[MH]^+$, $C_8H_6IO_4$ requires 292.9311, 100 %), 274.9206 ($[M – OH]^+$, $C_8H_4IO_3$ requires 274.9205, 20%).

Preparation of dimethyl 2-iodoisophthalate (S4) [29]

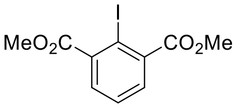

S4

Thionyl chloride (7.0 mL, 96.5 mmol) was added dropwise over 10 minutes to a solution of 2-iodoisophthalic acid (**S3**, 5.16 g, 17.7 mmol) in methanol (130 mL) at 0 °C under a nitrogen atmosphere. On complete addition, the reaction mixture was refluxed at 70 °C for 20 hours. The excess thionyl chloride was removed *in vacuo*, affording a residue which was extracted with ethyl acetate (70 mL). The organic phase was then washed with brine (50 mL), followed by water (2 × 50 mL), and the aqueous phase was extracted with ethyl acetate (30 mL). The organic phases were combined, dried ($MgSO_4$), filtered and the solvent was removed in vacuo to afford the crude product as an orange oil (6.22 g). The crude product was subsequently purified by column chromatography (SiO_2, 5% EtOAc in pet. ether, followed by EtOAc flush), to afford pure **S4** as a pale yellow solid (5.46 g, 96%). The characterisation data was in agreement with the literature data [37]. δ_H ($CDCl_3$, 400 MHz) 3.93 (6H, s, OCH_3), 7.42 (1H, t, $^3J_{HH}$ = 7.9 Hz, ArH), 7.62 (2H, d, $^3J_{HH}$ = 7.9 Hz, ArH). δ_C ($CDCl_3$, 100 MHz) 52.9 (OCH_3), 91.8 (CI), 128.1 (CH), 131.4 (CH), 139.8 (C), 168.1 (CO). m/z (ESI$^+$) 319.9547 ($[M]^+$, $C_{10}H_9IO_4$, requires 319.9546, 100%).

Preparation of 2,2'-(2-iodo-1,3-phenylene)bis(propan-2-ol) (8) [29]

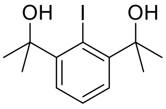

8

Magnesium turnings (1.55 g, 64.5 mmol) were charged to a dry 3-neck RBF, equipped with addition funnel and condenser, along with a few crystals of iodine, under a nitrogen atmosphere. Dry diethyl ether (10 mL) was added. Iodomethane (2.9 mL, 40.6 mmol) was charged to the addition funnel, along with dry diethyl ether (9 mL). This was subsequently added dropwise to the magnesium suspension, over 20 minutes, affording a gentle reflux throughout. On complete addition, the reaction mixture was stirred for 1 hour at room temperature.

The freshly prepared MeMgI solution was transferred under nitrogen by cannula to a new dry 3-neck RBF, equipped with addition funnel and condenser, and a further aliquot of dry diethyl ether (5 mL) was used to rinse the Grignard reagent into the clean flask. Dimethyl 2-iodoisophthalate (**S4**, 1.98 g, 6.2 mmol) was charged to the addition funnel in dry diethyl ether (15 mL), and was subsequently added to the MeMgI solution over 30 minutes at room temperature. On complete addition, the reaction mixture was refluxed for 2 hours. Hereafter, powdered iodine (7.58 g, 29.8 mmol) was dissolved in dry diethyl ether (30 mL) and subsequently charged to the addition funnel. This was then added dropwise to the reaction mixture, at room temperature, over 20 minutes. The reaction was then refluxed for 16 hours. After cooling the reaction mixture to 0 °C, it was quenched with a saturated NH_4Cl solution (30 mL), followed by water (30 mL), diethyl ether (30 mL) and aq. $Na_2S_2O_3$ (50 mL). This was left stirring at room temperature for 1 hour, filtered through Celite under suction, whereby the filtrate was extracted with diethyl ether (3 × 50 mL). The ether extracts were combined, dried ($MgSO_4$), filtered and the solvent was removed in vacuo to afford **8** as a pale yellow solid (1.90 g, 99%). The characterisation data was in agreement with the literature [29]. m.p. 142-143 °C (142-144 °C) [29]. δ_H ($CDCl_3$, 400 MHz) 1.85 (12H, s, CH_3), 2.95 (2H, s, OH), 7.27 (1H, t, $^3J_{HH}$ = 7.7 Hz, ArH), 7.53 (2H, d, $^3J_{HH}$ = 7.8 Hz, ArH). δ_C ($CDCl_3$, 100 MHz) 31.1 (CH_3), 75.6 (C), 94.3 (CI), 126.6 (CH), 128.1 (CH), 150.0 (C). m/z (ASAP) 303.0246 ($[M-OH]^+$, $C_{12}H_{16}IO$ requires 303.0246, 100 %).

Preparation of 1,1,4,4-tetramethyl-1*H*,4*H*-2aλ^3-ioda-2,3-dioxacyclopenta[*hi*]indene (10)

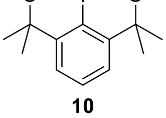

10

meta-Chloroperoxybenzoic acid (0.96 g, 4.3 mmol, 77% purity) was added portionwise to a solution of 2,2'-(2-iodo-1,3-phenylene)bis(propan-2-ol) (**8**, 1.33 g, 4.2 mmol) stirring in dichloromethane (85 mL) at room temperature. On complete addition, the reaction mixture was stirred at room temperature for 16 hours, after which the reaction mixture was washed with 10% aqueous Na_2CO_3 solution (20 mL). The phases were separated, and the organic phase was washed with brine (50 mL), then water (50 mL). The aqueous phases were combined and extracted with dichloromethane (50 mL). The organic extracts were combined, dried

($MgSO_4$), filtered and the solvent was removed in vacuo to afford a pale orange solid (1.38 g). The crude product was purified by column chromatography (40wtSiO_2, 1:1 EtOAc: P.E.) to afford 1,1,4,4-tetramethyl-1*H*,4*H*-2aλ^3-ioda-2,3-dioxacyclopenta[*hi*]indene (**10**) as a crystalline white solid (0.92 g, 70%). The characterisation data was in agreement with the literature [29]. m.p. 179-181 °C (lit. 169-171 °C) [29]. δ_H ($CDCl_3$, 400 MHz) 1.48 (12H, s, 2 × CH_3), 7.21 (2H, d, $^3J_{HH}$ = 7.3 Hz), 7.56 (1H, t, $^3J_{HH}$ = 7.3 Hz, ArH). δ_C ($CDCl_3$, 100 MHz) 29.9 (CH_3), 75.6 (C), 105.7 (CI), 123.7 (CH), 132.3 (CH), 148.0 (C). m/z (ESI^+) 319.0204 ($[MH]^+$, $C_{12}H_{16}O_2I$ requires 319.0195, 100%).

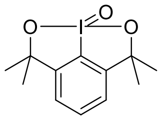

9

Compound **9** was also formed but was not isolated. δ_H ($CDCl_3$, 400 MHz) 1.60 (6H, s, CH_3), 1.66 (6H, s, CH_3), 7.50 (2H, d, $^3J_{HH}$ = 7.3 Hz), 7.84 (1H, t, $^3J_{HH}$ = 7.3 Hz, ArH).

Optimisation of Grignard step

Table S2 – Optimisation of the Grignard reaction to prepare **8**

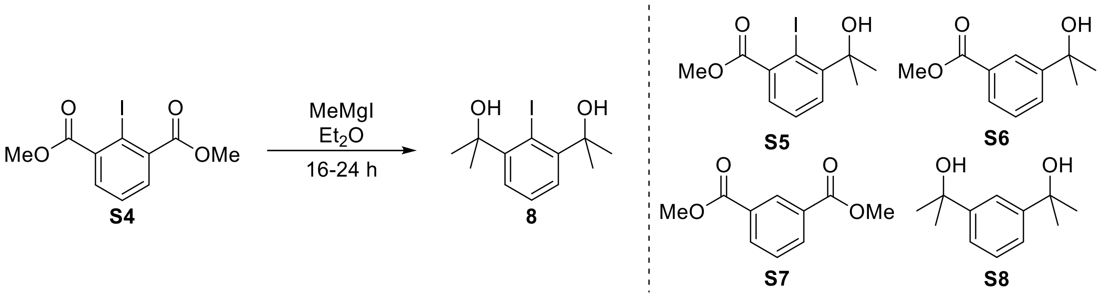

Entry	MeMgI (equiv)	Temp. (°C)	I_2 (equiv)	Product (%)[a]						Yield (%)
				**S4**	**8**	**S5**	**S6**	**S7**	**S8**	
**1**	5.0	25[b]	–	0	0	0	100	0	0	42 (**S6**)
**2**	5.0	25[b]	3	0	14	41	45	0	0	39 (**S5**)
**3**	2.4	25[b]	-	47	0	0	25	28	0	13 (**S7**)
**4**	5.0	Reflux[c]	3	0	39	48	13	0	0	26 (**8**)
**5**	7.5	Reflux[d]	3	0	72	0	0	0	28	50 (**8**)
**6**[f]	7.5	Reflux[e]	3	0	73	6	4	0	17	87 (**8**)
**7**[f]	7.5	Reflux[e]	3	0	77	0	0	0	23	48 (**8**)
**8**[f]	7.5	Reflux[e]	5	0	100	0	0	0	0	99 (**8**)

[a]Calculated by ^1H NMR analysis of the crude reaction mixture; [b]addition of **S4** at 0 °C then warmed to r.t. for stated time; [c]addition of **S4** at 0 °C followed by 1 hour reflux, before iodine addition then reflux for stated time; [d]addition of **S4** at room temperature followed by 4 hour reflux, before iodine addition at room temperature; [e]addition of **S4** at room temperature followed by 2 hour reflux, before iodine addition at room temperature; [f]concentration was higher than standard conditions, due to solvent loss in entry 6 and reduced volume of solvent in entries 7 and 8; all reactions were performed under a N_2 atmosphere.

Preparation of methyl 3-(2-hydroxypropan-2-yl)benzoate (S6) (Table S2, entry 1)

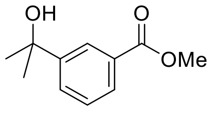

S6

Magnesium turnings (2.41 g, 99.0 mmol) were charged to a dry 3-neck RBF, equipped with addition funnel and condenser, along with a few crystals of iodine, under a nitrogen atmosphere. Dry diethyl ether (18 mL) was added and the solution was cooled to 0 °C. Iodomethane (4.5 mL, 72.3 mmol) was charged to the addition funnel, along with dry diethyl ether (18 mL). This was subsequently added dropwise to the magnesium suspension, over 20 minutes, affording a gentle reflux throughout. On complete addition, the reaction mixture was raised to room temperature and stirred for *ca.* 1 hour.

The freshly prepared MeMgI solution was transferred under nitrogen by cannula to a new dry 3-neck RBF, equipped with addition funnel and condenser, and a further aliquot of dry diethyl ether (10 mL) was used to rinse the Grignard reagent into the clean flask. This was subsequently cooled to 0 °C and dimethyl 2-iodoisophthalate (**S4**, 4.61 g, 14.4 mmol) was charged to the addition funnel with dry diethyl ether (40 mL), and was subsequently added to the MeMgI solution over 20 minutes. The reaction mixture was warmed to room temperature and left stirring for 22 hours. Hereafter, the reaction mixture was cooled to 0 °C and quenched with a saturated NH_4Cl solution (25 mL), followed by water (25 mL). This was left stirring at room temperature for 1.5 hours, filtered through Celite under suction, whereby the filtrate was extracted with diethyl ether (3 × 50 mL). The ether extracts were combined, dried ($MgSO_4$), filtered and the solvent was removed in vacuo to afford a pale yellow oil (3.17 g). The crude product was purified by column chromatography on silica gel (1:1 EtOAc:pet. ether) to afford pure **S6** as a pale yellow oil (1.17 g, 42%). δ_H ($CDCl_3$, 400 MHz) 1.61 (6H, s, 2 × CH_3), 1.78 (1H, s, OH), 3.92 (3H, s, OCH_3), 7.42 (1H, t, $^3J_{HH}$ = 7.8 Hz, ArH), 7.72 (1H, ddd, $^3J_{HH}$ = 7.8 Hz, $^4J_{HH}$ = 1.9 & 1.2 Hz, ArH), 7.93 (1H, dt, $^3J_{HH}$ = 7.7 Hz, $^4J_{HH}$ = 1.3 Hz, ArH), 8.16 (1H, t, $^4J_{HH}$ = 1.8 Hz, ArH). δ_C ($CDCl_3$, 100 MHz) 32.0 (CH_3), 52.3 (OCH_3), 72.6 (C), 125.8 (CH), 128.1 (CH), 128.5 (CH), 129.3 (CH), 130.3 (C), 149.7 (C), 167.4 (CO). m/z (ASAP) 177.0917 ($[M-OH]^+$, $C_{11}H_{13}O_2$ requires 177.0916, 100 %).

Preparation of dimethyl isophthalate (S7) (Table S2, entry 3)

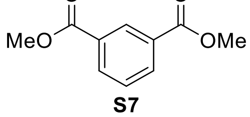

S7

Magnesium turnings (0.91 g, 37.6 mmol) were charged to a dry 3-neck RBF, equipped with addition funnel and condenser, along with a few crystals of iodine, under a nitrogen atmosphere. Dry diethyl ether (5 mL) was added. Iodomethane (1.65 mL, 26.5 mmol) was charged to the addition funnel, along with dry diethyl ether (5 mL). This was subsequently added dropwise to the magnesium suspension, over 10 minutes, affording a gentle reflux throughout. On complete addition, the reaction mixture was raised to room temperature and stirred for ca. 1 hour.

The freshly prepared MeMgI solution was transferred under nitrogen by cannula to a new dry 3-neck RBF, equipped with addition funnel and condenser, and a further aliquot of dry diethyl ether (5 mL) was used to rinse the Grignard reagent into the clean flask. The Grignard reagent was subsequently cooled to 0 °C and dimethyl 2-iodoisophthalate (**S4**, 3.53 g, 11.0 mmol) was charged to the addition funnel with dry diethyl ether (15 mL), and was subsequently added to the MeMgI solution over 15 minutes. The reaction mixture was warmed to room temperature and left stirring for 20 hours. Hereafter, the reaction mixture was cooled to 0 °C and quenched with a saturated NH_4Cl solution (20 mL), followed by water (150 mL). The solution was left stirring at room temperature for 1 hour, filtered through Celite under suction, whereby the filtrate was extracted with diethyl ether (3 × 50 mL). The ether extracts were combined, dried ($MgSO_4$), filtered and the solvent was removed *in vacuo* to afford a pale yellow oil (1.75 g). The crude product was purified by column chromatography on silica gel (60wtSiO_2, 1:1 EtOAc:pet. ether) to afford pure **S7** as a white solid (0.28 g, 13%). The characterisation data was in agreement with the literature [38]. m.p. 65-67 °C (lit., 63-65 °C) [38]. δ_H ($CDCl_3$, 500 MHz) 3.95 (6H, s, OCH_3), 7.52 (1H, t, $^3J_{HH}$ = 7.8 Hz, ArH) 8.22 (2H, dd, $^3J_{HH}$ = 7.8 Hz, $^4J_{HH}$ = 1.7 Hz , ArH), 8.67 (1H, t, $^4J_{HH}$ = 1.5 Hz). δ_C ($CDCl_3$, 125 MHz) 52.3 (OCH_3), 128.6 (CH), 130.6 (CH), 130.7 (C), 133.8 (CH), 166.2 (CO). m/z (ESI^+) 195.0658 ($[MH]^+$, $C_{10}H_{11}O_4$, requires 195.0657, 100%).

Dimethyl 2-iodoisophthalate (**S4**) and methyl 3-(2-hydroxypropan-2-yl)benzoate (**S6**) were isolated as a mixture (1.21 g, 3:2 (**S4**:**S6**)).

Preparation of methyl 3-(2-hydroxypropan-2-yl)-2-iodobenzoate (S5) [29] (Table S2, entry 2)

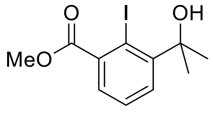

S5

Magnesium turnings (2.51 g, 103.7 mmol) were charged to a dry 3-neck RBF, equipped with addition funnel and condenser, along with a few crystals of iodine under a nitrogen atmosphere. Dry diethyl ether (13 mL) was added. Iodomethane (4.7 mL, 75.5 mmol) was charged to the addition funnel, along with dry diethyl ether (12 mL). This solution was subsequently added dropwise to the magnesium suspension over 30 minutes affording a gentle reflux throughout. On complete addition, the reaction mixture was stirred for 1 hour at room temperature.

The freshly prepared MeMgI solution was transferred under nitrogen by cannula to a new dry 3-neck RBF, equipped with addition funnel and condenser, and a further aliquot of dry diethyl ether (8 mL) was used to rinse the Grignard reagent into the clean flask. Dimethyl iodoisophthalate (**S4**, 4.82 g, 15.1 mmol) was charged to the addition funnel in dry diethyl ether (12 mL), and was subsequently added to the MeMgI solution over 15 minutes at 0 °C. On complete addition, the reaction mixture

was stirred at room temperature for 1 hour. Hereafter, powdered iodine (11.75 g, 46.3 mmol) was added portion wise to the reaction mixture, at 0 °C, and on complete addition, a further aliquot of dry diethyl ether (16 mL) was added. The reaction was then stirred at room temperature for 18 hours. After cooling the reaction mixture to 0 °C, it was quenched with a saturated NH_4Cl solution (30 mL), followed by water (30 mL), diethyl ether (30 mL) and aqueous $Na_2S_2O_3$ (30 mL, 3 M). This was left stirring at room temperature for 1 hour, filtered through Celite under suction, whereby the filtrate was extracted with diethyl ether (3 × 50 mL). The ether extracts were combined, dried ($MgSO_4$), filtered and the solvent was removed in vacuo to afford a crude orange solid (4.74 g). The crude product was purified by column chromatography on silica gel (40wt.SiO_2, 40% ethyl acetate in petroleum ether) to afford **S5** as an orange oil (2.03 g, 42%). The characterisation data was in agreement with the literature [29]. δ_H ($CDCl_3$, 500 MHz) 1.81 (6H, s, CH_3), 2.52 (1H, s, OH) 3.95 (3H, s, OCH_3), 7.22 (1H, dd, $^3J_{HH}$ = 7.7 Hz, $^4J_{HH}$ = 1.7 Hz, ArH), 7.37 (1H, t, $^3J_{HH}$ = 7.7 Hz, ArH), 7.72 (1H, dd, $^3J_{HH}$ = 7.7 Hz, $^4J_{HH}$ = 1.7 Hz, ArH). δ_C ($CDCl_3$, 125 MHz) 30.1 (CH_3), 52.9 (OCH_3), 74.5 (C), 90.8 (CI), 127.1 (CH), 128.3 (CH), 128.3 (CH), 143.1 (C), 149.9 (C), 170.4 (CO). m/z (ASAP) 302.9886 ($[M-OH]^+$, $C_{11}H_{12}IO_2$ requires 302.9882, 100 %).

Preparation of 2,2'-(1,3-phenylene)bis(propan-2-ol) (S8) (Table S2, entry 5)

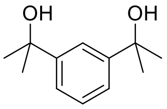

S8

Magnesium turnings (4.40 g, 181.1 mmol) were charged to a dry 3-neck RBF, equipped with addition funnel and condenser, along with a few crystals of iodine under a nitrogen atmosphere. Dry diethyl ether (24 mL) was added. Iodomethane (8.2 mL, 131.5 mmol) was charged to the addition funnel, along with dry diethyl ether (22 mL). This was subsequently added dropwise to the magnesium suspension over 20 minutes affording a gentle reflux throughout. On complete addition, the reaction mixture was stirred for 1 hour at room temperature.

The freshly prepared MeMgI solution was transferred under nitrogen by cannula to a new dry 3-neck RBF, equipped with addition funnel and condenser, and a further aliquot of dry diethyl ether (15 mL) was used to rinse the Grignard reagent into the clean flask. Dimethyl 2-iodoisophthalate (**S4**, 5.60 g, 17.5 mmol) was charged to the addition funnel in dry diethyl ether (20 mL), and was subsequently added to the MeMgI solution over 30 minutes at room temperature. On complete addition, the reaction mixture was refluxed for 4 hours. Hereafter, powdered iodine (13.46 g, 53.0 mmol) was dissolved in dry diethyl ether (70 mL) and subsequently charged to the addition funnel. This was then added dropwise to the reaction mixture, at room temperature, over 20 minutes. The reaction was then refluxed for 15 hours. After cooling the reaction mixture to 0 °C, it was quenched with a saturated NH_4Cl solution (30 mL), followed by water (30 mL), diethyl ether (30 mL) and aqueous $Na_2S_2O_3$ (50 mL). This was left stirring at room temperature for 1 hour, filtered through Celite under suction,

whereby the filtrate was extracted with diethyl ether (3 × 50 mL). The ether extracts were combined, dried ($MgSO_4$), filtered and the solvent was removed in vacuo to afford a crude yellow solid (4.60 g, 82%). The crude product was purified by column chromatography on silica gel (60wt., 1:1 EtOAc:pet. ether) to afford pure **8** as a white solid (2.81 g, 50%) and **S8** as a pale yellow solid (0.62 g, 18%). The characterisation data for **S8** was in agreement with the literature [39]. m.p. 130-132 °C (lit., 138-139 °C) [39]. δ_H ($CDCl_3$, 400 MHz) 1.60 (12H, s, CH_3), 7.30-7.33 (1H, m, ArH), 7.35-7.38 (2H, m, ArH), 7.67 (1H, t, $^4J_{HH}$ = 1.9 Hz, ArH). δ_C ($CDCl_3$, 100 MHz) 32.0 (CH_3), 72.9 (C), 120.4 (CH), 123.0 (CH), 128.2 (CH), 149.2 (C). m/z (ASAP) 177.1280 ($[M\text{-}OH]^+$, $C_{12}H_{17}O$ requires 177.1279, 100 %).

General procedure for oxidative fluorination of 8 with TCCA/KF (Table 1)

2,2'-(2-Iodo-1,3-phenylene)bis(propan-2-ol) (**8**, 0.52 g, 1.6 mmol), the required amount of TCCA (either 1.15 g, 4.9 mmol or 1.49 g, 6.4 mmol) and spray-dried KF (0.57 g, 9.8 mmol) were charged to a dry Schlenk flask under a nitrogen atmosphere. Dry acetonitrile (16 mL) was added to afford a suspension, which was stirred at 40 °C for either 24 or 48 hours. Hereafter, the reaction mixture was cooled to room temperature, filtered through celite under air or filtered via cannula into a new dry Schlenk flask under nitrogen. The solvent was removed in vacuo from the filtrate to afford a mixture of difluoroiodane **6** and iodosyl **9** (9:1, **6**:**9**) as a crude white solid.

Difluoroiodane **6** is not soluble in hexane and so, it could not be extracted away from the excess TCCA. We also tried to extract difluoroiodane **6** into other solvents such as dichloromethane, toluene and chloroform. Dichloromethane dissolved both TCCA and difluoroiodane **6**, whilst extraction with either toluene or chloroform led to isolation of the iodosyl byproduct **9**.

General procedure for oxidative fluorination of 8 with Selectfluor (Table 1, entry 5)

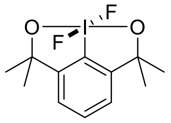

6

2,2'-(2-Iodo-1,3-phenylene)bis(propan-2-ol) (**8**, 0.58 g, 1.8 mmol) and Selectfluor (2.58 g, 7.3 mmol) were charged to a dry Schlenk flask under an argon atmosphere. Dry acetonitrile (18 mL) was added and the flask was sealed and heated to 40 °C for 48 hours. Hereafter, the reaction mixture was cooled to room temperature and solvent was removed *in vacuo* under inert conditions, to afford a crude orange solid. The orange solid was then extracted with dry dichloromethane (2 × 25 mL, 1 × 15 mL), filtered via a cannula transfer into a new dry Schlenk flask, where the solvent was removed in vacuo under inert conditions to afford difluoroiodane **6** as a pale orange solid (0.36 g, 56%). A small amount of iodosyl compound **9** was observed (0.02 g, 5%) along with a small amount of iodine(III) compound **10** (0.01 g, 1%).

General procedure for oxidative fluorination of 10 with Selectfluor (Table 2)

1,1,4,4-Tetramethyl-1*H*,4*H*-2aλ3-ioda-2,3-dioxacyclopenta[*hi*]indene (**10**, 0.51 g, 2 mmol) and the required amount of freeze-dried Selectfluor (3-10.2 mmol) were charged to a dry Schlenk flask under a nitrogen atmosphere. Dry acetonitrile (19 mL) was then added and the solution was heated at 40 °C for 6–24 hours in a sealed flask under nitrogen. Hereafter, the reaction was cooled to room temperature and the solvent was removed in vacuo under inert conditions to afford a crude orange solid. The crude solid was then extracted with dry dichloromethane (3 × 10 mL) and the suspension was filtered via a cannula transfer into a new dry Schlenk flask, where the solvent was removed *in vacuo* under inert conditions to afford difluoroiodane **6** as pale-yellow solid (55–91%).

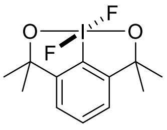
6

δ_H (CD_3CN, 400 MHz) 1.60 (12H, s, CH_3), 7.69 (2H, d, $^3J_{HH}$ = 7.4 Hz, ArH), 7.92 (1H, t, $^3J_{HH}$ = 7.3 Hz, ArH). δ_C (CD_3CN, 100 MHz) 32.6 (CH_3, t, $^4J_{CF}$ = 3.1 Hz), 80.0 (C), 125.1 (CH), 132.8 (CI, t, $^2J_{CF}$ = 11.9 Hz), 137.2 (CH), 146.6 (C). δ_F (CD_3CN, 376 MHz) -22.9 (2F, s, IF_2). m/z (ASAP$^+$) 337.0097 ([M-F]$^+$, $C_{12}H_{15}O_2FI$ requires 337.0101, 100%). A crystal suitable for X-ray diffraction was grown from a saturated dry acetonitrile solution that was placed under nitrogen and in a freezer for 48 hours.

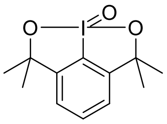
9

δ_H (CD_3CN, 400 MHz) 1.53 (6H, s, CH_3), 1.54 (6H, s, CH_3), 7.60 (2H, d, $^3J_{HH}$ = 7.4 Hz, ArH), 7.89 (1H, t, $^3J_{HH}$ = 7.4 Hz, ArH). δ_C (CD_3CN, 100 MHz) 31.3 (CH_3), 34.7 (CH_3), 80.0 (C), 125.3 (CH), 129.4 (CI), 136.8 (CH), 152.0 (C). m/z (ESI$^+$) 335.0135 ([MH]$^+$, $C_{12}H_{16}IO_3$ requires 335.0144, 100 %), 356.9957 ([MNa]$^+$, $C_{12}H_{15}O_3INa$ requires 356.9964, 15 %).

Preparation of hypervalent iodine(III) amides 11a and 11b

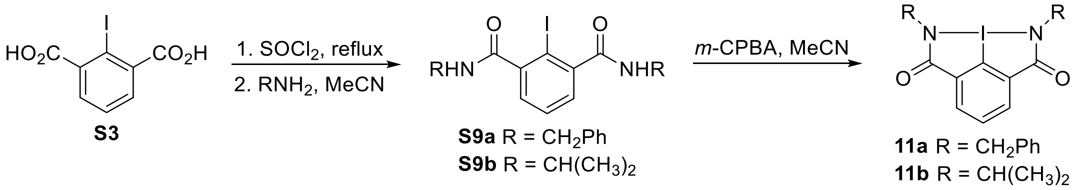

Preparation of N^1,N^3-dibenzyl-2-iodoisophthalamide (S9a)

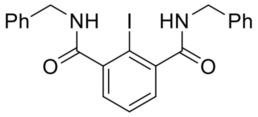
S9a

A dry round bottom flask, was charged with 2-iodoisophthalic acid (**S3**, 2.05 g, 7.0 mmol) and thionyl chloride (7.2 mL, 62.5 mmol) under nitrogen. The reaction mixture was refluxed for ca. 2.5 hours, after which excess thionyl chloride was removed in vacuo, affording a crude white solid. Benzylamine (5.2 mL, 47.6 mmol) was then added slowly over 10 minutes to a suspension of the crude white solid stirring in dry acetonitrile (16 mL) at 0 °C under nitrogen. On complete addition, the reaction mixture was stirred for a further

30 minutes at 0 °C, then warmed to room temperature for 16 hours. Hereafter, the solvent was removed in vacuo to afford a white solid which was washed with water (3 × 50 mL), dichloromethane (3 × 50 mL) and toluene (10 mL), followed by filtering to afford a white solid (2.12 g, 64%). m.p. 248-249 °C. δ_H (d_6-DMSO, 400 MHz) 4.45 (4H, d, $^3J_{HH}$ = 6.1 Hz, CH_2), 7.24-7.48 (13H, m, ArH), 8.94 (2H, t, $^3J_{HH}$ = 6.1 Hz, NH). δ_C (d_6-DMSO, 100 MHz) 42.5 (CH_2), 92.0 (CI), 126.8 (CH), 127.4 (CH), 127.8 (CH), 128.0 (CH), 128.3 (CH), 139.1 (C), 144.5 (C), 169.1 (CO). m/z (ESI^+) 471.0570 ($[MH]^+$, $C_{22}H_{20}IN_2O_2$ requires 471.0569, 100%).

Preparation of 2-Iodo-*N*¹,*N*³-diisopropylisophthalamide (S9b) [40]

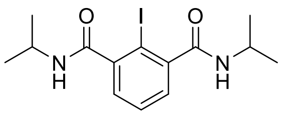

S9b

A dry round bottom flask, was charged 2-iodoisophthalic acid (**S3**, 2.08 g, 7.1 mmol) and thionyl chloride (7.3 mL, 63.4 mmol) under nitrogen. The reaction mixture was refluxed for ca. 2.5 hours, after which excess thionyl chloride was removed *in vacuo*, affording a crude white solid. A solution of dry triethylamine (6.0 mL, 43.3 mmol) and isopropylamine (3.7 mL, 43.1 mmol) in dry acetonitrile (8 mL) was added slowly over 10 minutes to a suspension of the crude white solid stirring in dry acetonitrile (8 mL) at 0 °C under nitrogen. On complete addition, the reaction mixture was stirred at 0 °C for another hour, then warmed to room temperature for 17 hours. Hereafter, solvent was removed in vacuo to afford a crude white solid, which was dissolved in dichloromethane (300 mL) and washed with water (3 × 100 mL). The organic phase was separated, dried ($MgSO_4$), filtered and the solvent was removed in vacuo to afford **S9b** as a pure white solid (1.66 g, 62%). The characterisation data was in agreement with the literature [40]. m.p. 271-272 °C (lit. 259.5 - 260.4 °C) [40]. δ_H ($CDCl_3$, 500 MHz) 1.28 (12H, d, $^3J_{HH}$ = 6.6 Hz, CH_3), 4.29 (2H, m, CH) 5.58 (2H, br d, $^3J_{HH}$ = 8.0 Hz, NH), 7.31 (2H, d, $^3J_{HH}$ = 6.4 Hz, ArH), 7.37 (1H, t, $^3J_{HH}$ = 6.4 Hz, ArH). δ_C ($CDCl_3$, 125 MHz) 22.7 (CH_3), 42.4 (CH), 90.8 (CI), 128.3 (CH), 128.6 (CH), 144.5 (C), 168.9 (CO). m/z (ESI^+) 375.0572 ($[MH]^+$, $C_{14}H_{20}IN_2O_2$ requires 375.0569, 100%), 397.0386 ($[MNa]^+$, $C_{14}H_{19}IN_2NaO_2$ requires 397.0389, 10%).

Preparation of 2,3-dibenzyl-2aλ³-ioda-2,3-diazacyclopenta[*hi*]indene-1,4(2*H*,3*H*)-dione (11a)

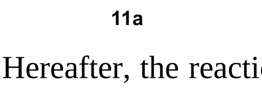

11a

meta-Chloroperoxybenzoic acid (0.49 g, 2.2 mmol. 77% purity) was added to a suspension of *N¹*,*N³*-dibenzyl-2-iodoisophthalamide (**S9a**, 0.51 g, 1.1 mmol) in acetonitrile (48 mL), and the solution was stirred at 30 °C for 48 hours. Hereafter, the reaction solution was cooled to room temperature and solvent was removed in vacuo to afford a white solid, which was suspended in diethyl ether (50 mL) and stirred for 1 hour. The suspension was filtered and the filter cake was washed with diethyl ether (3 × 10 mL), affording **11a**

as a white solid (0.41 g, 79%). This was then recrystallized (hot MeCN/hexane) to afford pure **11a** (0.27 g, 53%). M.p. 247-249 °C. δ_H (CD_3CN, 400 MHz) 4.62 (4H, s, CH_2), 7.28-7.33 (10 H, m, ArH), 7.93 (1H, t, $^3J_{HH}$ = 7.5 Hz, ArH), 8.22 (2H, d, $^3J_{HH}$ = 7.5 Hz, ArH). δ_C (CD_3CN, 100 MHz) 47.3 (CH_2), 115.5 (CI), 128.3 (CH), 128.8 (CH), 129.4 (CH), 131.0 (CH), 132.7 (C), 133.1 (CH), 139.8 (C), 164.0 (CO). m/z (ESI^+) 469.0413 ($[MH]^+$, $C_{22}H_{18}IN_2O_2$ requires 469.0413, 100%).

Preparation of 2,3-diisopropyl-2aλ^3-ioda-2,3-diazacyclopenta[hi]indene-1,4(2*H*,3*H*)-dione (11b) [40]

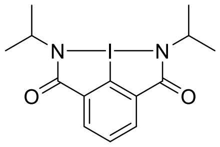

11b

meta-Chloroperoxybenzoic acid (0.72 g, 3.2 mmol, 77% purity) was added to a suspension of 2-iodo-N^1,N^3-diisopropylisophthalamide (**S9b**, 0.72 g, 1.9 mmol) in acetonitrile (28 mL) and the reaction mixture was stirred at room temperature for 24 hours. Hereafter, the solvent was removed in vacuo to afford a white solid, which was suspended in diethyl ether (50 mL) and filtered. The filter cake was washed with diethyl ether (3 × 50 mL), affording **11b** as a white powder (0.67 g, 93%). The white solid was then washed with 10% aq. Na_2CO_3 solution (20 mL) to afford pure **11b** as a white solid (0.54 g, 75%). The characterisation data was in agreement with the literature [40]. M.p 271-272 °C (lit. 259.5-260.4 °C) [40]. δ_H ($CDCl_3$, 500 MHz) 1.38 (12H, d, $^3J_{HH}$ = 6.6 Hz, CH_3), 4.38 (2H, sept, $^3J_{HH}$ = 6.6 Hz, CH), 7.88 (1H, t, $^3J_{HH}$ = 7.5 Hz, ArH), 8.29 (2H, d, $^3J_{HH}$ = 7.5 Hz, ArH). δ_C ($CDCl_3$, 125 MHz) 24.5 (CH_3), 46.2 (CH), 113.1 (CI), 131.1 (CH), 132.2 (CH), 132.7 (C), 162.9 (CO). m/z (ESI^+) 373.0413 ($[MH]^+$ $C_{14}H_{18}IN_2O_2$ requires 373.0413, 100%) 395.0223 ($[MNa]^+$, $C_{14}H_{17}INaN_2O_2$ requires 395.0232, 5%).

Attempted preparation of difluoro(aryl)-λ^5-iodane 7a with Selectfluor

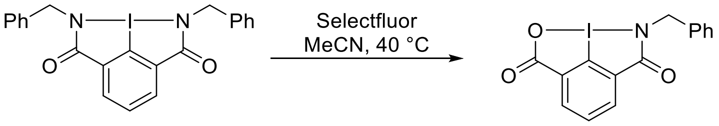

11a

A dry Schlenk flask was charged with **11a** (0.28 g, 0.6 mmol), freeze-dried Selectfluor (1.02 g, 2.9 mmol) and dry acetonitrile (10 mL) under a nitrogen atmosphere. The flask was sealed under nitrogen and the reaction solution was heated to 40 °C for 48 hours. Hereafter, the reaction solution was cooled to room temperature, where the solvent was removed in vacuo to afford a crude white solid. The crude solid was extracted with dry dichloromethane (10 mL), and filtered via cannula into a new dry Schlenk flask, where the solvent was removed in vacuo to afford a pale yellow solid. Analysis of the crude solid by ^{1}H NMR spectroscopy showed a mixture of iodine(III) compounds; proposed

iodine(III) product: δ_H (CD_3CN, 400 MHz) 4.79 (2H, s, CH_2), 7.37-7.43 (5H, m, ArH), 7.98 (1H, t, $^3J_{HH}$ = 7.5 Hz, ArH). 8.26-8.30 (2H, m, ArH).

For compound **11a** δ_H (CD_3CN, 400 MHz) 4.62 (4H, s, CH_2), 7.31 (10 H, m, ArH), 7.93 (1H, t, $^3J_{HH}$ = 7.5 Hz, ArH), 8.22 (2H, d, $^3J_{HH}$ = 7.5 Hz, ArH).

Attempted preparation of difluoro(aryl)-λ^5-iodane 7a with TCCA/KF

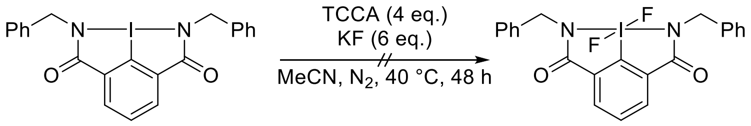

A dry Schlenk flask was charged with compound **11a** (0.28 g, 0.6 mmol), TCCA (0.56 g, 2.4 mmol) and spray-dried KF (0.22 g, 3.7 mmol), under a nitrogen atmosphere, followed by addition of dry acetonitrile (7.2 mL). The flask was sealed under nitrogen and the solution was heated to 40 °C for 48 hours. The solution was cooled to room temperature, filtered into a new dry Schlenk flask via cannula, and the solvent was removed in vacuo under inert conditions to afford a crude white solid. Analysis by ^{1}H and ^{19}F NMR spectroscopy showed an indecipherable mixture.

Attempted preparation of difluoro(aryl)-λ^5-iodane 7b with Selectfluor

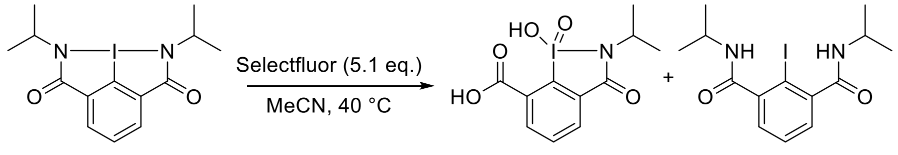

A dry Schlenk flask was charged with **11b** (0.40 g, 1.1 mmol), freeze-dried Selectfluor (1.93 g, 5.4 mmol) and dry acetonitrile (18.9 mL) under a nitrogen atmosphere. The flask was sealed under nitrogen and the reaction solution was heated to 40 °C for 48 hours. Hereafter, the reaction solution was allowed to cool to room temperature, where the solvent was removed in vacuo to afford a crude white solid. The crude solid was extracted with dry dichloromethane (10 mL), and filtered via cannula into a new dry Schlenk flask, where the solvent was removed in vacuo to afford a white solid. Analysis of the crude solid by ^{1}H NMR spectroscopy showed a mixture of products, alongside unreacted **11b**; proposed iodine(V) product: δ_H (CD_3CN, 400 MHz) 1.35 (6H, d, $^3J_{HH}$ = 6.5 Hz, CH_3), 4.33 (1H, sept., $^3J_{HH}$ = 6.5 Hz, CH), 7.95 (1H, t, $^3J_{HH}$ = 7.5 Hz, ArH), 8.23 (1H, dd, $^3J_{HH}$ = 7.5 Hz, $^4J_{HH}$ = 1.2 Hz, ArH), 8.27 (1H, dd, $^3J_{HH}$ = 7.5 Hz, $^4J_{HH}$ = 1.2 Hz, ArH). For compound **S9b**: δ_H (CD_3CN, 400 MHz)

1.21 (12H, d, $^3J_{HH}$ = 6.6 Hz, CH_3), 4.11 (1H, sept., $^3J_{HH}$ = 6.6 Hz, CH), 7.24 (2H, d, $^3J_{HH}$ = 7.2 Hz, ArH), 7.40 (1H, t, $^3J_{HH}$ = 7.2 Hz, ArH), 8.45 (2H, s, NH).

For compound **11b**: δ_H (CD_3CN, 400 MHz) 1.35 (12H, d, $^3J_{HH}$ = 6.5 Hz, CH_3), 4.27 (2H, sept., $^3J_{HH}$ = 6.6 Hz, CH), 7.89 (1H, t, $^3J_{HH}$ = 7.4 Hz, ArH), 8.15 (2H, d, $^3J_{HH}$ = 7.4 Hz, ArH).

Attempted preparation of difluoro(aryl)-λ^5-iodane 7b with TCCA/KF

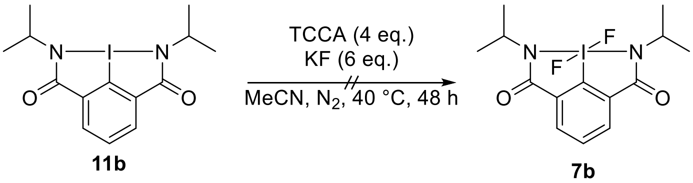

A dry Schlenk flask was charged with compound **11b** (0.41 g, 1.1 mmol), TCCA (1.04 g, 4.5 mmol) and spray-dried KF (0.32 g, 5.6 mmol), under a nitrogen atmosphere, followed by addition of dry acetonitrile (12 mL). The flask was sealed under nitrogen and the solution was heated to 40 °C for 48 hours. The solution was cooled to room temperature, filtered into a new dry Schlenk flask via cannula, and the solvent was removed in vacuo under inert conditions to afford a crude white solid. Analysis by ^{1}H and ^{19} F NMR spectroscopy showed an indecipherable mixture.

Procedure for the reaction of difluoroiodane 6 with PhMgCl

Difluoroiodane **6** (0.15 g, 0.4 mmol) was charged to a dry Schlenk flask under an inert atmosphere, followed by dry toluene (5 mL). The solution was cooled to 0 °C, then PhMgCl (0.2 mL, 2 M, 0.4 mmol) was added dropwise over several minutes. On complete addition, the solution was stirred at room temperature under an inert atmosphere for 1 hour, after which an internal standard (1-fluoro-3-nitrobenzene (85 µL, 0.8 mmol, 2 equiv, internal standard) was added and an aliquot was taken for NMR spectroscopy with a C_6D_6 insert. $\delta_F\{^1H\}$ (1-fluoro-4-nitrobenzene) -109.6 (1F, s, ArF).

Procedure for the reaction of difluoroiodane 6 with PhMgBr

Difluoroiodane **6** (0.07 g, 0.2 mmol) was charged to a dry Schlenk flask, under an inert atmosphere, followed by dry toluene (2 mL) and dry benzotrifluoride (22 µL, 0.2 mmol, 1 equiv, internal standard). The solution was cooled to 0 °C, then PhMgBr (0.2 mL, 1 M, 0.2 mmol) was added dropwise over several minutes. On complete addition, the solution was stirred at room temperature for 1 hour under an inert atmosphere, after which an aliquot was taken for NMR spectroscopy with a C_6D_6 insert. $\delta_F\{^1H\}$ (benzotrifluoride) -62.6 (3F, s, $ArCF_3$).

Preparation of 2’-iodoacetophenone (13) [41]

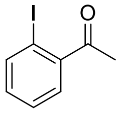

13

2’-Aminoacetophenone (**12**, 4.3 mL, 37.6 mmol) was added to a solution of *p*-toluenesulphonic acid (20.32 g, 106.8 mmol) in acetonitrile (150 mL) with stirring. The solution was cooled to 0 °C and sodium nitrite (6.06 g, 87.8 mmol) and potassium iodide (14.66 g, 88.3 mmol) in water (30 mL) was added dropwise over 1 hour and 20 minutes. On complete addition, the reaction mixture was raised to room temperature for 24 hours. Hereafter, the reaction was quenched with saturated $NaHCO_3$ (120 mL), followed by addition of aqueous $Na_2S_2O_3$ (40 mL, 3.3 M). The solution was separated and the aqueous phase was extracted with diethyl ether (4 × 100 mL). The organic extracts were combined and washed with brine (20 mL), then the organic phase was dried ($MgSO_4$), filtered and the solvent removed in vacuo to afford a dark orange oil (8.08 g). The orange oil was redissolved in diethyl ether (50 mL) and washed with 2 M HCl (3 × 50 mL), then brine (20 mL), and the aqueous phase was combined and extracted with diethyl ether (50 mL). The organic extracts were combined and dried ($MgSO_4$), filtered and solvent was removed in vacuo to afford 2’-iodoacetophenone **13** as a dark orange oil (7.76 g, 84%). The characterization data was in agreement with the literature [42]. δ_H ($CDCl_3$, 400 MHz) 2.62 (3H, s, CH_3), 7.13 (1H, td, $^3J_{HH}$ = 7.9 Hz, $^4J_{HH}$ = 1.8 Hz, ArH), 7.41 (1H, td, $^3J_{HH}$ = 7.7 Hz, $^4J_{HH}$ = 0.9 Hz, ArH), 7.47 (1H, dd, $^3J_{HH}$ = 7.7 Hz, $^4J_{HH}$ = 1.8 Hz, ArH), 7.94 (1H, dd, $^3J_{HH}$ = 7.9 Hz, $^4J_{HH}$ = 0.9 Hz, ArH). δ_C ($CDCl_3$, 100 MHz) 29.7 (CH_3), 91.1 (CI), 128.2 (CH), 128.5 (CH), 132.0 (CH), 141.1 (CH), 144.2 (C), 202.0 (CO). m/z (ESI^+) 246.9614 ($[MH]^+$, C_8H_8IO requires 246.9620, 100%).

Preparation of 1,1,1-trifluoro-2-(2-iodophenyl)propan-2-ol (14)

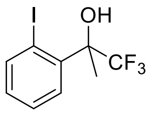

14

2’-Iodoacetophenone (**13**, 4.23 g, 17.2 mmol) was placed in dry THF (22 mL) in a dry RBF under a nitrogen atmosphere. The solution was cooled to 0 °C and (trifluoromethyl)trimethylsilane (5.4 mL, 36.6 mmol) was added, followed by addition of tetrabutylammonium fluoride in dry THF (4.6 mL, 1 M, 4.6 mmol) over 10 minutes. On complete addition, the reaction solution was stirred at 0 °C for 30 minutes, then raised to room temperature for 18 hours. Hereafter, the reaction was quenched with water (50 mL), then extracted with diethyl ether (4 × 50 mL). The organic extracts were combined, washed with water (2 × 50 mL), then brine (100 mL). The organic phase was then hydrolysed with 2 M HCl in THF (100 mL, 1:1) by stirring at room temperature for 4 hours. The phases were separated and the aqueous phase was extracted with diethyl ether (3 × 50 mL). The organic extracts were combined and washed with brine (100 mL). The organic phase was dried ($MgSO_4$), filtered and the solvent was removed in vacuo to afford a dark brown oil (6.84 g). The ^1H NMR spectrum of the crude product showed 88% conversion from 2’-iodoacetophenone **13**. Therefore, the crude oil was dissolved in dry THF (22 mL), in a dry RBF under

nitrogen and cooled to 0 °C. (Trifluoromethyl)trimethylsilane (5.4 mL, 36.6 mmol) was added, followed by addition of tetrabutylammonium fluoride in dry THF (4.6 mL, 1 M, 4.6 mmol) over 10 minutes. On complete addition the solution was stirred at room temperature for 22 hours. Hereafter, the reaction was quenched with water (50 mL), then extracted with diethyl ether (4 × 50 mL). The organic extracts were combined, washed with water (2 × 50 mL), then brine (100 mL). The organic phase was then hydrolysed with 2M HCl in THF (100 mL, 1:1) by stirring at room temperature for 4 hours. The phases were separated and the aqueous phase was extracted with diethyl ether (3 × 50 mL). The organic extracts were combined and washed with brine (100 mL). The organic phase was dried ($MgSO_4$), filtered and the solvent was removed in vacuo to afford a dark orange oil (7.11 g). The crude oil was purified by column chromatography (40 wtSiO_2, 10% ethyl acetate in petroleum ether, then 1:1 ethyl acetate: petroleum ether) to afford **14** as an orange oil (5.05 g, 93%). The characterisation data was in agreement with the literature [43]. δ_H ($CDCl_3$, 400 MHz) 1.91 (3H, q, $^4J_{HF}$ = 1.0 Hz, CH_3), 3.35 (1H, s, OH), 7.00 (1H, td, $^3J_{HH}$ = 7.9 Hz, $^4J_{HH}$ = 1.7 Hz, ArH), 7.37 (1H, td, $^3J_{HH}$ = 8.2 Hz, $^4J_{HH}$ = 1.4 Hz, ArH), 7.54 (1H, d, $^3J_{HH}$ = 8.2 Hz, ArH), 8.04 (1H, dd, $^3J_{HH}$ = 7.9 Hz, $^4J_{HH}$ = 1.3 Hz, ArH). δ_C ($CDCl_3$, 100 MHz) 23.8 (CH_3), 76.6 (C, q, $^2J_{CF}$ = 29.0 Hz), 92.2 (CI), 125.8 (C, q, $^1J_{CF}$ = 286.7 Hz), 128.2 (CH), 129.4 (CH, q, $^4J_{CF}$ = 2.4 Hz), 130.3 (CH), 139.0 (C), 143.9 (CH). δ_F ($CDCl_3$, 376 MHz) -78.0 (3F, s, CF_3). m/z (ESI^-) 314.9506 ($[M\text{-}H]^-$, $C_9H_7F_3IO$ requires 314.9494, 100%), (ESI^+) 332.9611 ($[(M\text{+}OH)]^+$, $C_9H_9F_3IO_2$ requires 332.9599, 100%).

Preparation of 1-fluoro-3-methyl-3-(trifluoromethyl)-1,3-dihydro-1λ³-benzo[*d*][1,2]iodaoxole (15)

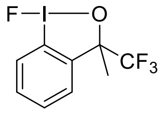

15

A solution of **14** (0.90 g, 2.9 mmol) in dry acetonitrile (40 mL) and freeze-dried Selectfluor (2.12 g, 6.0 mmol) were added to a dry Schlenk flask under a nitrogen atmosphere. The flask was sealed and the solution was stirred at room temperature for 24 hours. Hereafter, the solvent was removed in vacuo to afford crude yellow solid which was then extracted with warm chloroform (3 × 30 mL). The organic extracts were combined and the solvent was removed in vacuo to afford **15** as a crude yellow solid (1.16 g). The crude solid was then recrystallised from warm toluene to afford a pale yellow solid (0.67 g, 68%). m.p. 105-106 °C. δ_H ($CDCl_3$, 400 MHz) 1.70 (3H, s, CH_3), 7.39 (1H, br d, $^3J_{HH}$ = 7.2 Hz, ArH), 7.57 (1H, td, $^3J_{HH}$ = 7.4 Hz, $^4J_{HH}$ = 1.4 Hz, ArH), 7.72 (1H, td, $^3J_{HH}$ = 7.8 Hz, $^4J_{HH}$ = 1.1 Hz, ArH), 7.81 (1H, br d, $^3J_{HH}$ = 8.3 Hz, ArH). δ_C ($CDCl_3$, 100 MHz) 23.2 (CH_3), 84.9 (C, q, $^2J_{CF}$ = 29.4 Hz), 116.8 (CI), 126.2 (CF_3, q, $^1J_{CF}$ = 288.3 Hz), 128.5 (CH), 128.7 (CH), 130.9 (CH), 132.3 (CH), 139.0 (C). δ_F ($CDCl_3$, 400 MHz) -80.9 (3F, s, CF_3), -158.2 (1F, s, IF). m/z (ESI^+) 314.9501 ($[M\text{-}F]^+$, $C_9H_7F_3IO$ requires 314.9494, 100%), 332.9604 ($[M\text{-}H]^+$, $C_9H_6F_4IO$ requires 332.9599, 100%).

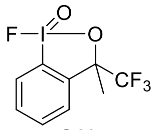

S10

A small amount of iodosyl **S10** was also formed, but it was not isolated (0.05 g, 4%). δ_H (CD_3CN, 400 MHz) 1.73 (3H, q, $^4J_{HF}$ = 1.0 Hz, CH_3), 1.81 (3H, q, $^4J_{HF}$ = 1.0 Hz, CH_3), 7.80, (1H, m, ArH), 7.93 (2H, m, ArH), 8.24 (1H, m, ArH). δ_F (CD_3CN, 376 MHz) -34.3 (1F, s, IF), -34.8 (1F, s, IF) -80.2 (3F, s, CF_3), -80.5 (3F, s, CF_3).

Preparation of 2,2,2-trifluoro-1-(2-iodophenyl)ethan-1-one (17)

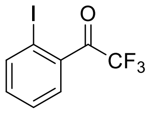

17

(Trifluoromethyl)trimethylsilane (4.0 mL, 27.0 mmol) was added to a solution of methyl 2-iodobenzoate **16** (4.72 g, 18.0 mmol) stirring in dry toluene (75 mL) at –78 °C. Afterwards tetrabutylammonium fluoride in THF (1M, 1.0 mL, 1.0 mmol) was added to the reaction mixture. On complete addition, the reaction temperature was increased to room temperature and stirred for 24 hours (monitored by TLC, 10% ethyl acetate in petroleum ether). The reaction mixture was then cooled in ice, quenched with 4 M HCl solution (30 mL) and stirred for a further 15 minutes at room temperature. Then the layers were separated and the aqueous phase was extracted with ethyl acetate (3 × 50 mL). The organic extracts were combined, washed with brine (100 mL), dried ($MgSO_4$), filtered and the solvent was removed in vacuo to afford a crude orange oil. The crude product was purified by column chromatography (20 wtSiO_2, 5% ethyl acetate in petroleum ether) to afford 2,2,2-trifluoro-1-(2-iodophenyl)ethan-1-one **17** as a crystalline yellow solid (3.76 g, 70%). The data was in agreement with the literature [44]. m.p. 43-45 °C (lit. 40.2 - 40.7 °C) [44]. δ_H ($CDCl_3$, 400 MHz) 7.29 (1H, td, $^3J_{HH}$ = 7.9 Hz, $^4J_{HH}$ = 1.6 Hz, ArH), 7.51 (1H, td, $^3J_{HH}$ = 7.7 Hz, $^4J_{HH}$ = 1.2 Hz, ArH), 7.73 (1H, dquin, $^3J_{HH}$ = 7.9 Hz, $^4J_{HH}$ & $^5J_{HF}$ = 1.5 Hz, ArH), 8.10 (1H, dd, $^3J_{HH}$ = 8.0 Hz, $^4J_{HH}$ = 1.0 Hz, ArH). δ_C ($CDCl_3$, 100 MHz) 93.9 (CI), 115.7 (C, q, $^1J_{CF}$ = 292.0 Hz), 128.0 (CH), 130.1 (CH), 134.2 (CH), 134.6 (C), 142.3 (CH), 182.6 (CO, q, $^2J_{CF}$ = 36.1 Hz). δ_F ($CDCl_3$, 376 MHz) -72.3 (3F, d, $^5J_{HF}$ = 1.7 Hz, CF_3). m/z ($ASAP^+$) 300.9334 ($[MH]^+$, $C_8H_5F_3OI$ requires 300.9337, 100 %).

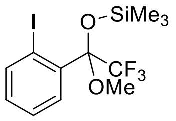

S11

Silyl ether **S11** was observed in the crude reaction mixture by ^{1}H NMR spectroscopy, but it was not isolated. δ_H ($CDCl_3$, 400 MHz) 0.34 (9H, s, $Si(CH_3)_3$), 3.25 (3H, s, OCH_3) 7.00 (1H, td, $^3J_{HH}$ = 7.3 Hz, $^4J_{HH}$ = 1.7 Hz, ArH), 7.37-7.41 (1H, m, ArH), 7.68 (1H, dq, $^3J_{HH}$ = 8.0 Hz, $^4J_{HH}$ & $^5J_{HF}$ = 0.8 Hz, ArH), 8.07 (1H, dd, $^3J_{HH}$ = 8.0 Hz, $^4J_{HH}$ = 1.7 Hz, ArH).

Preparation of 1,1,1,3,3,3-hexafluoro-2-(2-iodophenyl)propan-2-ol (18)

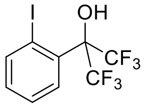

18

(Trifluoromethyl)trimethylsilane (3.4 mL, 25.7 mmol) was added to a solution of 2,2,2-trifluoro-1-(2-iodophenyl)ethan-1-one (**17**, 2.27 g, 7.6 mmol) stirring in dry toluene (36 mL) at room temperature under a nitrogen atmosphere. Afterwards tetrabutylammonium fluoride in dry tetrahydrofuran (1 M, 22.6 mL, 22.6 mmol) was added slowly to the reaction mixture (an exotherm was observed). On complete addition, the reaction was stirred at room temperature for 24 hours, after which 4 M HCl solution (40 mL) was added slowly, and the reaction was left to stir for a further 24 hours. Hereafter, the layers were separated, and the aqueous phase was extracted with ethyl acetate (3 × 80 mL). The organic extracts were combined, washed with water (100 mL) then brine (100 mL), dried ($MgSO_4$), filtered and the solvent was removed in vacuo to afford a crude orange oil (3.08 g). The crude product was purified by column chromatography (20 wt SiO_2, 5% ethyl acetate and 2% triethylamine in petroleum ether) to afford 1,1,1,3,3,3-hexafluoro-2-(2-iodophenyl)propan-2-ol (**18**) as a colourless oil (2.32 g, 83%). The characterisation data was in agreement with the literature [45]. δ_H ($CDCl_3$, 400 MHz) 4.29 (1H, br s, OH), 7.11 (1H, td, $^3J_{HH}$ = 7.7 Hz, $^4J_{HH}$ = 1.6 Hz, ArH), 7.43 (1H, ddd, $^3J_{HH}$ = 8.3 & 7.2 Hz, $^4J_{HH}$ = 1.4 Hz, ArH), 7.64 (1H, d, $^3J_{HH}$ = 8.1 Hz, ArH), 8.13 (1H, dd, $^3J_{HH}$ = 8.0 Hz, $^4J_{HH}$ = 1.4 Hz, ArH). δ_C ($CDCl_3$, 100 MHz) 78.8 (C, septet, $^2J_{CF}$ = 30.0 Hz), 90.4 (CI), 122.7 (C, q, $^1J_{CF}$ = 289.7 Hz), 127.9 (CH), 129.6 (C), 129.8 (CH), 131.3 (CH), 144.6 (CH). δ_F ($CDCl_3$, 376 MHz) -73.5 (6F, s, CF_3). m/z (ESI^- direct infusion) 368.9211 ($[M-H]^-$, $C_9H_4F_6IO$ requires 368.9211, 100%).

Preparation of 1-fluoro-3,3-bis(trifluoromethyl)-1,3-dihydro-1λ^3-benzo[*d*][1,2]iodaoxole (19)

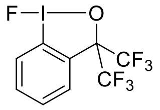

19

A dry Schlenk flask was charged with a solution of iodoalcohol **18** (0.38 g, 1.0 mmol) in dry acetonitrile (14 mL) and freeze-dried Selectfluor (0.55 g, 1.6 mmol), under a nitrogen atmosphere. The flask was sealed and the solution was stirred at room temperature for 24 hours. Hereafter, solvent was removed in vacuo to afford a crude white solid. The solid was then extracted with dry dichloromethane (3 × 10 mL), filtered via cannula transfer to a new dry Schlenk flask where solvent was removed in vacuo under inert conditions. Fluoroiodane **19** was afforded as a white solid (0.29 g, 72%). The characterisation data was in agreement with the literature [46]. δ_H (CD_3CN, 500 MHz) 7.75 (2H, m, ArH), 7.86 (1H, m, ArH), 7.93 (1H, m, ArH). δ_C (CD_3CN, 126 MHz) 87.2 (C, sept., $^2J_{CF}$ = 30.7 Hz), 117.8 (CI, d, $^2J_{CF}$ = 9.8 Hz), 124.6 (CF_3, q, $^1J_{CF}$ = 289.6 Hz), 129.7 (CH), 129.8 (CH), 130.7 (C, sept, $^3J_{CF}$ = 2.3 Hz), 132.9 (CH), 135.6 (CH). δ_F (CD_3CN, 471 MHz) -76.7 (6F, s, CF_3), -173.5 (1F, s, IF). m/z (ESI^+) 368.9208 ($[M-F]^+$, $C_9H_4F_6IO$ requires 368.9211, 100%), 386.9315 ($[M-H]^+$, $C_9H_3F_7IO$ requires 386.9317, 100%).

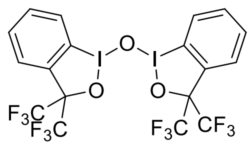
S12

A small amount of **S12** was also formed and the characterisation data was in agreement with the literature (0.01 g, 5%) [47]. δ_H (CD_3CN, 400 MHz) 7.75 (1H, dd, $^3J_{HH}$ = 6.9 Hz, $^4J_{HH}$ = 1.1 Hz, ArH), 7.80 (1H, m, ArH), 7.94 (1H, m, ArH), 8.01 (1H, dd, $^3J_{HH}$ = 7.2 Hz, $^4J_{HH}$ = 1.1 Hz, ArH). δ_C (CD_3CN, 100 MHz) 85.6 (C, m), 117.8 (CI), 125.0 (CF_3, q, $^1J_{CF}$ = 289.9 Hz), 128.4 (CH), 129.0 (CH), 130.8 (C, sept, $^3J_{CF}$ = 2.5 Hz), 132.4 (CH), 134.8 (CH). δ_F (CD_3CN, 376 MHz) -76.7 (12F, s, CF_3). m/z (ESI^+) 754.8454 ($[MH]^+$, $C_{18}H_9F_{12}I_2O_3$ requires 754.8449, 100%).

Preparation of 1,1,1-trifluoro-3,3-dimethyl-1,3-dihydro-1λ⁵-benzo[*d*][1,2]iodaoxole (20) (Table 3, entry 1)

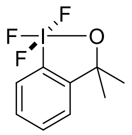
20

Selectfluor (1.93 g, 5.5 mmol) was charged to a dry Schlenk flask under a nitrogen atmosphere. A solution of fluoroiodane **2** (0.61 g, 2.2 mmol) in dry acetonitrile (12 mL) was added and the flask was sealed under nitrogen and heated to 40 °C for 24 hours. Hereafter, the reaction was cooled to room temperature and solvent was removed in vacuo under inert conditions to afford a crude solid. This solid was extracted with dry dichloromethane (3 × 20 mL), the solution was filtered via cannula transfer into a new dry Schlenk flask, where solvent was removed in vacuo under inert conditions to afford a **20** as a pale yellow solid (0.52 g, 75 %). δ_H (CD_3CN, 400 MHz) 1.67 (6H, s, CH_3), 7.70 (1H, dt, $^3J_{HH}$ = 8.0 Hz, $^4J_{HH}$ = 1.6 Hz, ArH), 7.81 (1H, td, $^3J_{HH}$ = 7.9 Hz, $^4J_{HH}$ = 1.3 Hz, ArH), 7.85 (1H, td, $^3J_{HH}$ = 7.5 Hz, $^4J_{HH}$ = 1.0 Hz, ArH), 8.24 (1H, dt, $^3J_{HH}$ = 8.2 Hz, $^4J_{HH}$ = 1.4 Hz, ArH). δ_C (CD_3CN, 100 MHz) 32.2 (CH_3), 89.7 (C), 125.2 (CH, d, $^3J_{CF}$ = 9.0 Hz), 126.7 (CH), 130.9 (CH), 134.9 (CH, d, $^4J_{CF}$ = 1.3 Hz), 146.3 (C, q, $^2J_{CF}$ = 2.0 Hz), 147.3 (CI, q, $^2J_{CF}$ = 11.6 Hz). δ_F (CD_3CN, 376 MHz) -20.2 (2F, d, $^2J_{FF}$ = 107.5 Hz, IF_2), -34.0 (1F, t, $^2J_{FF}$ = 107.5 Hz, IF). m/z ($ASAP^+$) 298.9739 ($[M\text{-}F]^+$, $C_9H_{10}F_2IO$ requires 298.9744, 100%).

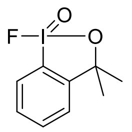
S13

A small amount of iodosyl fluoride **S13** was also formed (0.02 g, 3%). δ_H (CD_3CN, 400 MHz) 1.52 (3H, s, CH_3), 1.64 (3H, s, CH_3), 7.57 (1H, dd, $^3J_{HH}$ = 7.7 Hz, $^4J_{HH}$ = 1.2 Hz, ArH), 7.71 (1H, ddd, $^3J_{HH}$ = 8.2 & 7.1 Hz, $^4J_{HH}$ = 1.2 Hz, ArH), 7.80 (1H, td, $^3J_{HH}$ = 7.7 Hz, $^4J_{HH}$ = 1.0 Hz, ArH), 8.07 (dd, $^3J_{HH}$ = 8.2 Hz, $^4J_{HH}$ = 1.0 Hz, ArH). δ_C (CD_3CN, 100 MHz) 31.4 (CH_3), 35.0 (CH_3), 90.9 (C), 128.0 (CH), 129.4 (CH), 131.8 (CH), 135.6 (CH), 141.9 (C), 153.1 (CI). δ_F (CD_3CN, 376 MHz) -29.9 (F, s, IF). m/z (ESI^+) 276.9730 ($[M\text{-}F]^+$, $C_9H_{10}IO_2$ requires 276.9725, 100%).

Preparation of 1,1,1-trifluoro-3-methyl-3-(trifluoromethyl)-1,3-dihydro-1λ^5-benzo[*d*][1,2]-iodaoxole (21) (Table 3, entry 2)

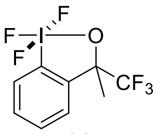

21

A dry Schlenk flask was charged with a solution of **15** (0.33 g, 1.0 mmol) in dry acetonitrile (9.5 mL) and freeze-dried Selectfluor (1.05 g, 4.1 mmol), under a nitrogen atmosphere. The flask was sealed and the solution was heated to 60 °C for 48 hours with stirring. Hereafter, the reaction solution was cooled to room temperature and solvent was removed *in vacuo* to afford a crude orange solid. The crude product was extracted with dry dichloromethane (3 × 15 mL) and filtered via cannula transfer into a new dry Schlenk flask, where solvent was removed in vacuo under inert conditions to afford **21** as a pale yellow solid (0.29 g, 78%). δ_H (CD_3CN, 400 MHz) 1.85 (3H, s, CH_3), 7.89 (1H, m, ArH), 7.96 (2H, m, ArH), 8.33 (1H, dt, $^3J_{HH}$ = 7.8 Hz, $^4J_{HH}$ = 1.5 Hz, ArH). δ_C (CD_3CN, 100 MHz) 25.8 (CH_3), 88.3 (C, q, $^2J_{CF}$ = 31.6 Hz), 126.1 (CH, d, $^3J_{CF}$ = 9.2 Hz), 126.5 (CF_3, q, $^1J_{CF}$ = 284.3 Hz), 128.7 (CH), 133.9 (CH), 136.1 (CH), 137.1 (C), 148.8 (CI, q, $^2J_{CF}$ = 11.2 Hz). δ_F (CD_3CN, 376 MHz) -17.4 (1F, ddq, $^2J_{FF}$ = 139.1 & 119.8 Hz, $^5J_{FF}$ = 6.8 Hz, IF), -19.8 (1F, dd, $^2J_{FF}$ = 139.0 & 107.6 Hz, IF), -38.5 (1F, dd, $^2J_{FF}$ = 120.1 & 107.8 Hz, IF), -80.2 (3F, d, $^5J_{FF}$ = 6.8 Hz, CF_3). m/z ($ASAP^+$) 352.9463 ($[M-F]^+$, $C_9H_7F_5OI$ requires 352.9462, 100 %).

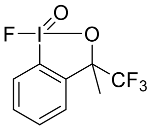

S10

Iodosyl compound **S10** was also formed, but it was not isolated (0.01 g, 2%). δ_H (CD_3CN, 400 MHz) 1.73 (3H, q, $^4J_{HF}$ = 1.0 Hz, CH_3), 1.81 (3H, q, $^4J_{HF}$ = 1.0 Hz, CH_3), 7.80 (1H, m, ArH), 7.93 (2H, m, ArH), 8.24 (1H, m, ArH). δ_F (CD_3CN, 376 MHz) -34.3 (1F, s, IF), -34.8 (1F, s, IF) -80.2 (3F, s, CF_3), -80.5 (3F, s, CF_3).

Preparation of 1,1,1-trifluoro-3,3-bis(trifluoromethyl)-1,3-dihydro-1λ^5-benzo[*d*][1,2]iodaoxole (22) (Table 3, entry 3)

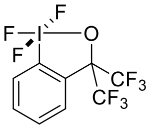

22

A dry Schlenk flask was charged with a solution of **19** (0.8 mmol) in dry acetonitrile (8 mL) and freeze-dried Selectfluor (0.88 g, 2.5 mmol) under a nitrogen atmosphere. The flask was sealed and the solution was heated to 80 °C for 72 hours with stirring. Hereafter, the reaction was cooled to room temperature and solvent was removed in vacuo to afford a crude orange solid. The crude product was extracted with dry dichloromethane (2 × 6 mL) and filtered via cannula transfer into a new dry Schlenk flask, where solvent was removed *in vacuo* to afford a pale-yellow solid (0.25 g, 72%). δ_H (CD_3CN, 400 MHz) 8.07 (2H, m, ArH), 8.15 (1H, td, $^3J_{HH}$ = 7.6 Hz, $^4J_{HH}$ = 1.6 Hz, ArH), 7.93 (1H, m, ArH). δ_C (CD_3CN, 100 MHz) 86.9 (C, sept, $^2J_{CF}$ = 32.2 Hz), 123.6 (CF_3, q, $^1J_{CF}$ = 288.1 Hz), 126.4 (CH, d, $^3J_{CF}$ = 9.3 Hz), 128.1 (C), 129.6 (CH), 135.9 (CH), 136.6 (CH), 149.5 (CI, q, $^2J_{CF}$ = 11.2 Hz). δ_F (CD_3CN, 376 MHz) -16.7 (2F, dsept, $^2J_{FF}$

= 119.2 Hz, ${}^{5}J_{FF}$ = 3.4 Hz, IF_2), -43.9 (1F, t, ${}^{2}J_{FF}$ = 119.0 Hz, IF), -75.9 (6F, t, ${}^{5}J_{FF}$ = 3.4 Hz, CF_3). m/z (ESI^+ direct infusion) 406.9182 ($[M-F]^+$, $C_9H_4F_8IO$ requires 406.9179, 100%).

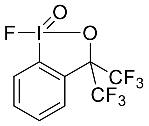
S14

Iodosyl compound **S14** was also formed but not isolated (0.01 g, 4%). δ_H (CD_3CN, 400 MHz) 8.00 (2H, m, ArH), 8.05 (1H, m, ArH), 8.33 (1H, m, ArH). δ_C (CD_3CN, 400 MHz) 88.2 (C, sept, ${}^{2}J_{CF}$ = 31.3 Hz), 123.9 (CF_3, q, ${}^{1}J_{CF}$ = 288.2 Hz), 130.1 (CH), 130.2 (CH), 134.2 (C), 135.5 (CH), 136.2 (CH), 145.4 (CI). δ_F (CD_3CN, 376 MHz) -39.7 (1F, s, IF), -76.0 (6F, s, CF_3).

Preparation of 1,1,1-trifluoro-3,3-bis(trifluoromethyl)-1,3-dihydro-1λ⁵-benzo[*d*][1,2]iodaoxole (22) from iodine(I) precursor 18

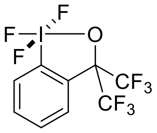
22

A dry Schlenk flask was charged with a solution of iodoalcohol **18** (0.44 g, 1.2 mmol) in dry acetonitrile (13 mL) and freeze-dried Selectfluor (2.34 g, 6.6 mmol) under a nitrogen atmosphere. The flask was sealed and the suspension was stirred at room temperature for 24 hours. Subsequently, the solution was heated to 90 °C for 72 hours with stirring. Hereafter, the reaction was cooled to room temperature and solvent was removed in vacuo to afford a crude orange solid. The crude product was extracted with dry dichloromethane (3 × 10 mL) and filtered via cannula transfer into a new dry Schlenk flask, where solvent was removed in vacuo to afford **22** as a white solid (0.37 g, 73%), along with a trace amount of iodosyl compound **S14** (<0.01 g, 1%).

Table S3: Reaction between trifluoroiodane **22** and PhMgBr (Scheme 6)

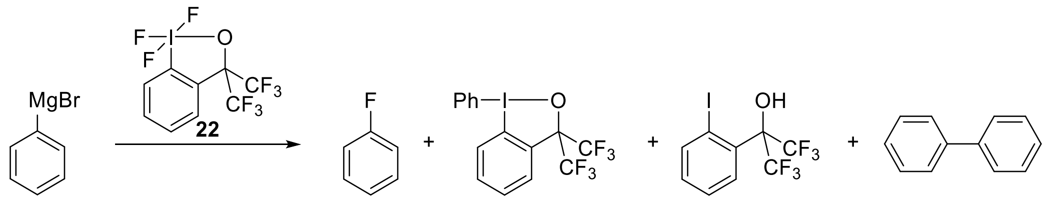

					Spectroscopic yield (isolated)			
			time	Temp.	S15	23	18	S16[a]
Entry	Solvent	Additive	(h)	(°C)	(%)	(%)	(%)	(%)
**1**	hexane	-	4	rt	0	0 (12)	35 (0)	0 (32)
**2[b]**	toluene	-	4	rt	0	0	43 (56)	0 (29)
**3**	toluene	-	4	0	0	0	0	0
**4**	toluene	-	1	0 °C – rt	0	0 (23)	9 (25)	0 (12)
**5[c]**	toluene	$BF_3{\cdot}OEt_2$	1	0 °C – rt	0	0 (7)	86 (30)	0 (10)
**6[d]**	toluene	$BF_3{\cdot}OEt_2$	1	0 °C – rt	1	0	76 (69)	0 (23)
**7[e]**	toluene	$BF_3{\cdot}OEt_2$	1	0 °C – rt	3	0 (16)	69 (12)	0 (35)
**8[f]**	toluene	$B(C_6F_5)_3$	1	0 °C – rt	0	0 (33)	0 (11)	0 (20)
**9[d]**	Et_2O	$BF_3{\cdot}OEt_2$	1	0 °C – rt	0	0 (23)	67 (50)	0 (31)
**10[g]**	toluene	$BF_3{\cdot}OEt_2$	1	0 °C – rt	0	0	0	0

[a]Biphenyl **S16** was a product of the reaction but it was also formed as a byproduct in the preparation of 1 M PhMgBr in diethyl ether in ca. 10% yield; [b]reverse addition, trifluoroiodane was added to PhMgBr; [c]PhMgBr and 0.5 equiv of $BF_3{\cdot}OEt_2$ were mixed prior to addition of trifluoroiodane **22** at 0 °C; [d]0.5 equiv of $BF_3.OEt_2$ added to trifluoroiodane **22** prior to PhMgBr addition; [e]2 equiv of **22**; [f]0.5 equiv of tris(pentafluorophenyl)borane added ($B(C_6F_5)_3$); [g]no trifluoroiodane **22** was added.

General procedure for reaction of PhMgBr with trifluoroiodane 22 (Scheme 6)

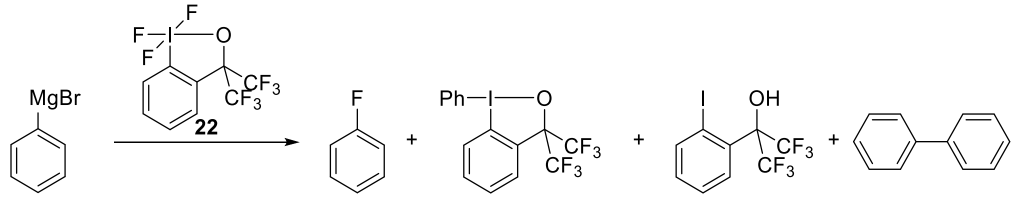

A dry Schlenk flask was charged with trifluoroiodane **22** (0.13 g, 0.3 mmol), dry toluene (3.7 mL) and dry benzotrifluoride (38 µL, 0.3 mmol, 1 equiv of internal standard) under a nitrogen atmosphere. The solution was cooled to 0 °C and an activator was added, followed by slow addition of

phenylmagnesium bromide (0.3 mL, 1 M in diethylether, 0.3 mmol). On complete addition, the solution was stirred at room temperature for 1–4 hours, after which an aliquot was taken for in situ NMR analysis with a C_6D_6 insert and spectroscopic yields for **18** and **S15** were calculated using benzotrifluoride. δ_F (benzotrifluoride) -62.6 (3F, s, $ArCF_3$), (**18**) -73.1 (6F, s, CF_3), (**S15)** -112.8 (1F, s, ArF).

Hereafter, the in situ NMR sample was recombined with the reaction solution, and water (2 mL) was added. The organic layer was decanted, and the aqueous was extracted with dichloromethane (3 × 2 mL). The organic extracts were combined and the solvent was removed in vacuo to afford a crude oil (0.15 g). The crude oil was purified by column chromatography (100 wtSiO_2, gradient elution; petroleum ether, 10% ethyl acetate in petroleum ether, 20% ethyl acetate in petroleum ether then 1:1 ethyl acetate:petroleum ether).

Phenyliodane **23** was isolated as a white solid and the following data was in agreement with the literature [48]. δ_H ($CDCl_3$, 400 MHz) 6.80 (1H, dd, $^3J_{HH}$ = 8.3 Hz, $^4J_{HH}$ = 0.9 Hz ArH), 7.34 (1H, ddd, $^3J_{HH}$ = 8.4 & 7.1 Hz, $^4J_{HH}$ = 1.5 Hz, ArH), 7.54 (3H, m, ArH), 7.69 (1H, tt, $^3J_{HH}$ = 7.5 Hz, $^4J_{HH}$ = 1.5 Hz), 7.89 (3H, m, ArH). δ_C (CD_3Cl, 100 MHz) 81.3 (C, m), 111.5 (CH), 119.0 (CI), 124.4 (CF_3, q, $^1J_{CF}$ = 290.5 Hz), 127.8 (CH), 130.5 (CH), 130.5 (CH), 131.1 (CH), 131.3 (C), 132.0 (CH), 132.2 (C), 137.0 (CH). δ_F (CD_3Cl, 376 MHz) -76.04 (6F, s, CF_3). High resolution mass spectrometry could not be collected as sample did not ionise.

Reaction of methyl(trifluoromethyl)trifluoroiodane (21) with PhMgBr

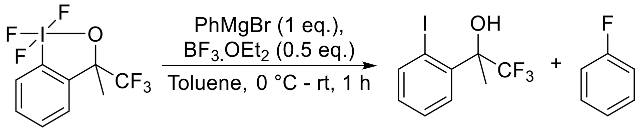

21 **14** **S15**

A dry Schlenk flask was charged with trifluoroiodane **21** (0.08 g, 0.2 mmol), dry benzotrifluoride (26 μL) and dry toluene (3.4 mL) to afford a clear solution, under a nitrogen atmosphere. The solution was then cooled to 0 °C, and boron trifluoroide etherate (0.15 mL, 0.8 M, 0.12 mmol) was added, followed by slow addition of phenylmagnesium bromide (0.2 mL, 1 M, 0.2 mmol). On complete addition, the solution was stirred at room temperature for 1 hour. A sample was taken for in situ NMR analysis and **S15** was formed in 3% spectroscopic yield. δ_F (benzotrifluoride) -62.6 (3F, s, $ArCF_3$), (**14**) -77.7 (3F, s, CF_3), (**S15**) -113.6 (1F, s, ArF).

Hereafter, the reaction solution was quenched with water (2 mL) and the organic phase was decanted. The aqueous was extracted with toluene (3 × 2 mL) and the organic extracts were combined and

solvent was removed in vacuo to afford a crude oil (0.15 g), which was purified by column chromatography (100wtSiO_2, gradient elution, petroleum ether, 5% ethyl acetate in petroleum ether, 30% ethyl acetate in petroleum, 1:1 ethyl acetate to petroleum ether) to afford iodoalcohol **14** as a yellow oil (0.06 g, 82%).

Reaction of dimethyltrifluoroiodane 20 with PhMgBr

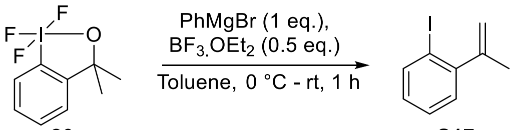

A dry Schlenk flask was charged with trifluoroiodane **20** (0.10 g, 0.3 mmol), dry benzotrifluoride (38 µL, 0.3 mmol) and dry toluene (3.4 mL) to afford a clear solution, under a nitrogen atmosphere. The solution was then cooled to 0 °C, and boron trifluoroide etherate (0.2 mL, 0.8 M, 0.16 mmol) was added, followed by slow addition of phenylmagnesium bromide (0.3 mL, 1 M, 0.3 mmol). On complete addition, the solution was stirred at room temperature for 1 hour. A sample was taken for in situ NMR analysis but no fluorobenzene was formed. δ_F (benzotrifluoride) -62.6 (3F, s, $ArCF_3$).

Hereafter, the reaction solution was quenched with water (2 mL) and the organic phase was decanted. The aqueous was extracted with toluene (3 × 2 mL) and the organic extracts were combined and solvent was removed in vacuo to afford a crude oil (0.15 g), which was purified by column chromatography (100 wtSiO_2, gradient elution, petroleum ether, 5% ethyl acetate in petroleum ether, 30% ethyl acetate in petroleum, 1:1 ethyl acetate to petroleum ether) to afford **S17** as a yellow oil (0.02 g, 31%). The data was in agreement with the literature [49]. δ_H (CD_3Cl, 400 MHz) 4.89 (1H, s, $C\mathit{H_A}H_B$), 5.22 (1H, s, $CH_A\mathit{H_B}$), 6.94 (1H, t, $^3J_{HH}$ = 7.0 Hz, ArH), 7.18 (1H, d, $^3J_{HH}$ = 7.3 Hz, ArH), 7.30 (1H, t, $^3J_{HH}$ = 7.3 Hz, ArH), 7.84 (1H, d, $^3J_{HH}$ = 7.0 Hz, ArH). δ_C (CD_3Cl, 100 MHz) 24.0 (CH_3), 97.1 (CI), 116.2 (CH_2), 128.2 (CH), 128.5 (CH), 128.7 (CH), 139.3 (CH), 148.6 (C), 149.0 (C). m/z ($ASAP^+$) 243.9750 ($[M]^+$, C_9H_9I requires 243.9749, 100 %).

General procedure for monitoring the stability of hypervalent iodine(V) fluorides 6, 20, 21 and 22 in acetonitrile-*d*3 under argon

The samples for the stability studies were prepared in the glovebox under an argon atmosphere. To a J Young's NMR tube was charged hypervalent iodine(V) fluoride (0.05 mmol), dry and degassed benzotrifluoride (6 µL, 0.05 mmol, 1 equiv) and dry acetonitrile-d_3 (0.6 mL). The NMR tube was

sealed under argon and studied by ^{1}H and ^{19}F NMR spectroscopy over 7 days. Key NMR data for each hypervalent iodine(V) fluoride are shown below.

General procedure for monitoring the stability of hypervalent iodine(V) fluorides 6, 20, 21 and 22 in acetonitrile-*d*$_3$ under air

The samples for the stability studies were prepared in the glovebox under an argon atmosphere. To a J Young's NMR tube was charged hypervalent iodine(V) fluoride (0.05 mmol), dry and degassed benzotrifluoride (6 µL, 0.05 mmol, 1 equiv) and dry acetonitrile-*d*$_3$ (0.6 mL). The NMR tube was sealed under argon and ^{1}H and ^{19}F NMR spectra were collected immediately. Hereafter, the *J* Youngs NMR tube was opened to air for ca. 5 minutes, then resealed under an air atmosphere and the solution was studied by ^{1}H and ^{19}F NMR spectroscopy over 7 days.

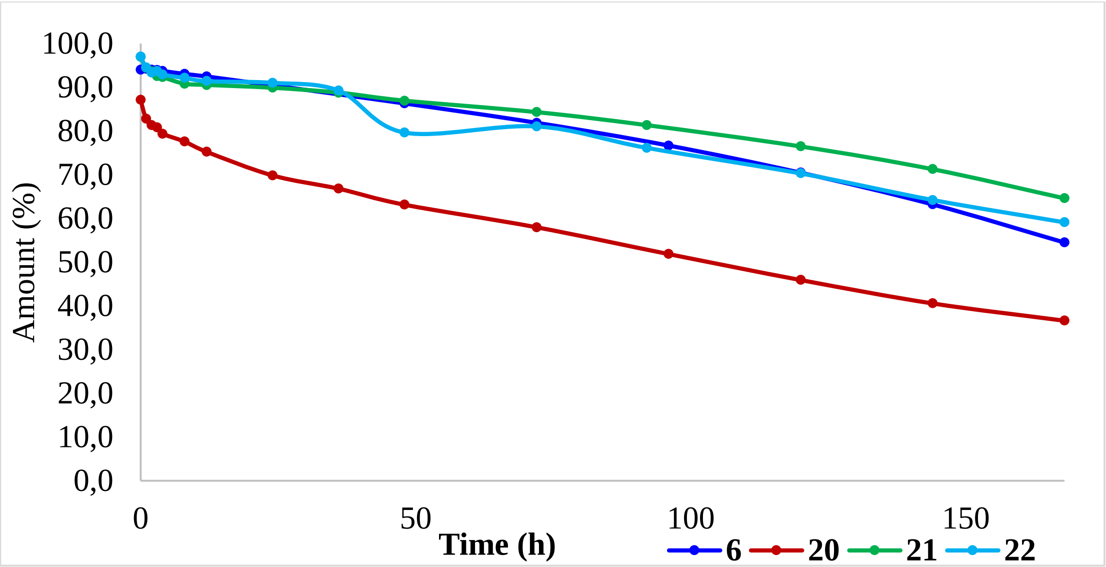

Figure S4: Stability of hypervalent iodine(V) fluorides **6** (dark blue line), **20** (red line), **21** (green line) and **22** (light blue) in CD_3CN under air at room temperature

Key NMR data used in the stability studies:

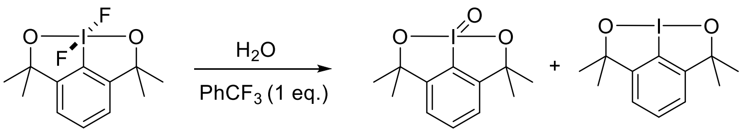

δ_H (CD_3CN, 400 MHz) 1.40 (12H, s, CH_3, **10**), 1.52 (6H, s, CH_3, **9**), 1.54 (6H, s, CH_3, **9**), 1.60 (12H, s, CH_3, **6**), 7.64 (1H, m, ArH, $PhCF_3$).

$\delta_F\{^1H\}$ (CD_3CN, 376 MHz) -22.9 (2F, s, IF_2, **6**), -63.2 (3F, s, CF_3, $PhCF_3$).

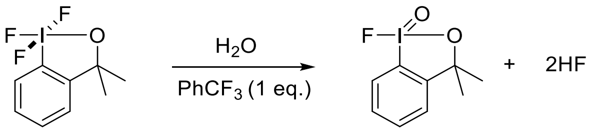

20 **S13**

δ_H (CDCN, 400 MHz) 1.57 (3H, s, CH_3, **S13**), 1.65 (3H, s, CH_3, **S13**), 1.69 (6H, s, CH_3, **20**), 7.64 (1H, m, ArH, $PhCF_3$).

$\delta_F\{^1H\}$ (CD_3CN, 376 MHz) -20.1 (2F, d, $^2J_{FF}$ = 107.5 Hz, IF_2, **20**), -33.9 (1F, t, $^2J_{FF}$ = 107.5 Hz, IF, **20**), -29.8 (1F, s, **S13**), -63.2 (3F, s, CF_3, $PhCF_3$).

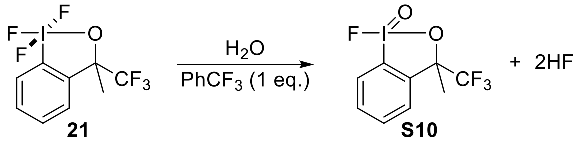

21 **S10**

δ_H (CD_3CN, 400 MHz) 1.74 (3H, q, $^4J_{HF}$ = 1.0 Hz, CH_3, **S10**), 1.82 (3H, q, $^4J_{HF}$ = 1.0 Hz, CH_3, **S10**), 1.86 (3H, s, CH_3, **21**), 7.64 (1H, m, ArH, $PhCF_3$).

$\delta_F\{^1H\}$ (CD_3CN, 376 MHz) -17.3 (1F, ddq, $^2J_{FF}$ = 139.1 & 119.8 Hz, $^5J_{FF}$ = 6.8 Hz, IF, **21**), -19.7 (1F, dd, $^2J_{FF}$ = 139.0 & 107.6 Hz, IF, **21**), -34.2 (1F, s, IF, **S10**), -34.7 (1F, s, IF, **S10**), -38.5 (1F, dd, $^2J_{FF}$ = 120.1 & 107.8 Hz, IF, **21**), -63.2 (3F, s, CF_3, $PhCF_3$), -80.2 (3F, d, $^5J_{FF}$ = 6.8 Hz, **21**), -80.1 (3F, s, CF_3, **S10**), -80.4 (3F, s, CF_3, **S10**), -183.3 (1F, br s, HF).

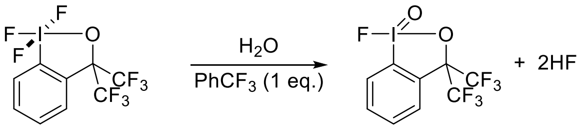

22 **S14**

$\delta_F\{^1H\}$ (CD_3CN, 376 MHz) -16.7 (2F, dsept, $^2J_{FF}$ = 119.2 Hz, $^5J_{FF}$ = 3.4 Hz, IF_2, **22**), -39.6 (1F, s, **S14**), -43.8 (1F, t, $^2J_{FF}$ = 119.0 Hz, IF, **22**), -63.2 (3F, s, CF_3, $PhCF_3$), -75.8 (6F, t, $^5J_{FF}$ = 3.4 Hz, CF_3, **22**), -75.9 (6F, s, CF_3, **S14**), -183.3 (1F, br s, HF).

General procedure for monitoring the stability of difluoroiodane 6 in chloroform-d_1 under argon

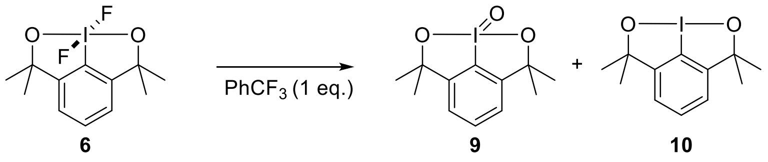

The sample for the stability study was prepared in the glovebox under an argon atmosphere. To a J Young's NMR tube was charged difluoroiodane **6** (18.8 mg, 0.05 mmol), dry and degassed benzotrifluoride (6 µl, 0.05 mmol, 1 equiv) and dry chloroform-d_1 (0.6 mL). The NMR tube was sealed under argon and studied by ^{1}H and ^{19}F NMR spectroscopy over 7 days. δ_H ($CDCl_3$, 400 MHz) 1.49 (12H, s, CH_3, **10**), 1.62 (6H, s, CH_3, **9**), 1.65 (6H, s, CH_3, **9**), 1.67 (12H, s, CH_3, **6**), 7.63 (2H, m, ArH, $PhCF_3$). $\delta_F\{^1H\}$ ($CDCl_3$, 376 MHz) -21.8 (2F, s, IF_2, **6**), -62.7 (3F, s, CF_3, $PhCF_3$).

General procedure for monitoring the stability of difluoroiodane 6 in chloroform-d_1 under air

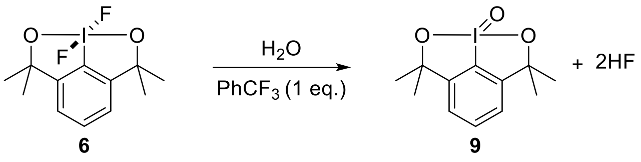

Samples for solution stability studies were prepared in the glovebox under an argon atmosphere. To a J Young's NMR tube was charged difluoroiodane **6** (18.8 mg, 0.05 mmol), dry and degassed benzotrifluoride (6 µl, 0.05 mmol, 1 equiv) and dry chloroform-d_1 (0.6 mL). The NMR tube was sealed under argon and ^{1}H and ^{19}F NMR spectra were collected immediately. Hereafter, the J Youngs NMR tube was opened to air for ca. 5 minutes, then resealed under an air atmosphere and the solution was studied by ^{1}H and ^{19}F NMR spectroscopy over 2 days, after which complete decomposition was seen. δ_H ($CDCl_3$, 400 MHz) 1.62 (6H, s, CH_3, **9**), 1.65 (6H, s, CH_3, **9**), 1.67 (12H, s, CH_3, **6**), 7.63 (2H, m, ArH, $PhCF_3$). $\delta_F\{^1H\}$ ($CDCl_3$, 376 MHz) -21.8 (2F, s, IF_2, **6**), -62.7 (3F, s, CF_3, $PhCF_3$).

General procedure for monitoring the stability of difluoroiodane 6 in chloroform-*d*1 and pyridine under air

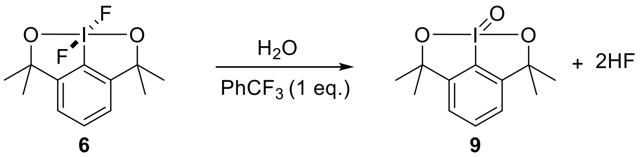

Samples for solution stability studies were prepared in the glovebox under an argon atmosphere. To a J Young's NMR tube was charged difluoroiodane **6** (18.0 mg, 0.05 mmol), dry and degassed benzotrifluoride (6 µl, 0.5 mmol, 1 equiv), dry pyridine (10 µl, 0.12 mmol, 2.4 equiv) and dry chloroform-d_1 (0.6 mL). The NMR tube was sealed under argon and ^{1}H and ^{19}F NMR spectra were collected immediately. Hereafter, the J Youngs NMR tube was opened to air for *ca.* 5 minutes, then resealed under an air atmosphere and the solution was studied by ^{1}H and ^{19}F NMR spectroscopy over 7 days. δ_H ($CDCl_3$, 400 MHz) 1.62 (6H, s, CH_3, **9**), 1.65 (6H, s, CH_3, **9**), 1.67 (12H, s, CH_3, **6**), 7.63 (2H, m, ArH, $PhCF_3$). $\delta_F\{^1H\}$ ($CDCl_3$, 376 MHz) -21.8 (2F, s, IF_2, **6**), -62.7 (3F, s, CF_3, $PhCF_3$).

General procedure for hydrolysing hypervalent iodine(V) fluorides 6, 20, 21 and 22 with water in acetonitrile-*d*3

Hypervalent iodine(V) fluoride (0.2 mmol) was added to a dry J Young's NMR tube, followed by dry d_3-acetonitrile (0.6 mL) and dry benzotrifluoride (20 µl, 0.2 mmol). The NMR tube was sealed under argon and ^{1}H and ^{19}F NMR spectra were collected. Hereafter, the solution was exposed to air and water (18 µl, 1.0 mmol, 5 equiv) was added, then $^{19}F\{^1H\}$ NMR spectra were collected immediately after and at 1-minute intervals over 20 minutes. The rate of hydrolysis of hypervalent iodine(V) fluoride was calculated from the rate of change of hypervalent iodine(V) fluorides integral relative to benzotrifluoride ($\delta_F\{^1H\}$ -63.23 (3F, s, $ArCF_3$)).

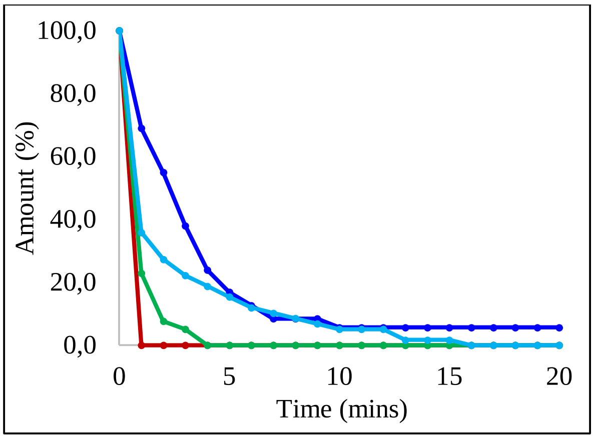

Figure S5: Hydrolysis of hypervalent iodine(V) fluorides **6** (dark blue line), **22** (light blue line), **21** (green line) and **20** (red line).

Structure solution and refinement

Table S4 summarises the crystallographic data for hypervalent iodine(V) compounds **6** and **9**. The data for the compounds were collected on a Bruker Apex 2000 CCD diffractometer using graphite monochromated Mo-Kα radiation (λ = 0.71073 Å). The data were corrected for Lorentz and polarisation effects, and empirical absorption corrections were applied. The structures were solved by direct methods and refined by full-matrix least squares cycles on F^2 for all data, using SHELXTL version 6.10 [50]. All hydrogen atoms were included in calculated positions (C–H = 0.95–0.99 Å) riding on the bonded atom with isotropic displacement parameters set to 1.5 Ueq (O) for hydroxyl H atoms, 1.5 Ueq (C) for methyl H atoms and 1.2 Ueq (C) for all other H atoms. All non-hydrogen atoms were refined with anisotropic displacement parameters. Data was collected on a Bruker D8 Quest Photon III diffractometer. The crystal was kept at 150.0 K during data collection. Using Olex2 [51], the structure was solved with the SHELXT [52] structure solution program using Intrinsic Phasing and refined with the SHELXL [53] refinement package using Least Squares minimisation.

Crystallographic data (excluding structure factors) for the structures reported in this paper have been deposited with the Cambridge Crystallographic Data Centre and allocated the deposition numbers CCDC: 2351949-2351950. Copies of the data can be obtained free of charge from the Cambridge Crystallographic Data Centre via www.ccdc.cam.ac.uk/data_request/cif.

Table S4: Crystal data and structure refinement for difluoroiodane 6 and iodosyl 9

	**6**		**9**	
Empirical formula	$C_{12}H_{15}F_2IO_2$		$C_{44}H_{52}Cl_2I_3N_7O_{15}$	
Formula weight	356.14		1370.53	
Temperature	150(2) K		150(2) K	
Wavelength	0.71073 Å		0.71073 Å	
Crystal system	Orthorhombic		Monoclinic	
Space group	Pbca		P2(1)/n	
Unit cell dimensions	a = 12.691(5) Å	α= 90°	a = 14.533(12) Å	α = 90°
	b = 15.804(6) Å	β= 90°	b = 22.613(19) Å	β= 95.754(19)°
	c = 25.354(10) Å	γ = 90°	c = 16.155(13) Å	γ = 90°
Volume	5085(3) $Å^3$		5282(8) $Å^3$	
Z	16		4	
Density (calculated)	1.861 Mg/m^3		1.723 Mg/m^3	
Absorption coefficient	2.531 mm^{-1}		1.947 mm^{-1}	
F(000)	2784		2712	
Crystal size	0.55 × 0.48 × 0.33 mm^3		0.23 × 0.16 × 0.08 mm^3	
Data collection range	1.61 to 26.00°.		1.55 to 26.00°.	
Index ranges	-15<=h<=15, -19<=k<=19, -30<=l<=31		-17<=h<=17, -27<=k<=27, -19<=l<=19	
Reflections collected	37140		41022	
Independent reflections	4987 [R(int) = 0.1084]		10380 [R(int) = 0.1377]	
Completeness to theta	100.0 %		100.0 %	
Absorption correction	Empirical		Empirical	
Max. and min. transmission	0.901 and 0.346		0.856 and 0.548	
Refinement method	Full-matrix least-squares on F^2		Full-matrix least-squares on F^2	
Data / restraints / parameters	4987 / 0 / 315		10380 / 2 / 653	
Goodness-of-fit on F^2	1.183		0.907	
Final R indices [I>2sigma(I)]	R1 = 0.0540, wR^2 = 0.1091		R1 = 0.0645, wR^2 = 0.1232	
R indices (all data)	R1 = 0.0649, wR^2 = 0.1131		R1 = 0.1150, wR^2 = 0.1386	
Largest diff. peak and hole	1.529 and -1.640 e.$Å^{-3}$		3.055 and -1.050 e.$Å^{-3}$	

References

33. Zhang, C.; Liu, S. S.; Sun, B.; Tian, J. *Org. Lett.*, **2015**, *17*, 4106–4109.

34. Perry, G. J. P.; Quibell, J. M.; Panigrahi, A.; Larrosa, I. *J. Am. Chem. Soc.*, **2017**, *139*, 11527–11536.

35. Wakelin, L. P. G.; Bu, X.; Eleftheriou, A.; Parmar, A.; Hayek, C.; Stewart, B. W. *J. Med. Chem.*, **2003**, *46*, 5790–5802.

36. Agosta, W. C. *Tetrahedon. Lett.*, **1965**, *6*, 2681–2685.

37. Kato, S.; Matsuoka, T.; Suzuki, S.; Asano, M. S.; Yoshihara, T.; Tobita, S.; Matsumoto, T.; Kitamura, C. *Org. Lett.*, **2020**, *22*, 734–738.

38. Lee, J. J.; Kraus, G. A. *Green Chem*, **2014**, *16*, 2111–2116.

39. Johnson, R. A.; Hall, C. M.; Krueger, W. C.; Murray, H. C. *Bioorganic Chem.*, **1973**, *2*, 99–110.

40. Yoshimura, A.; Shea, M. T.; Makitalo, C. L.; Jarvi, M. E.; Rohde, G. T.; Saito, A.; Yusubov, M. S.; Zhdankin, V. V. *Beilstein J. Org. Chem.*, **2018**, *14*, 1016–1020.

41. Yasui, T.; Kikuchi, T.; Yamamoto, Y.; *Chem. Commun.*, **2020**, *56*, 12865–12868.

42. Boelke, A.; Kuczmera, T. J.; Caspers, L. D.; Lork, E.; Nachtsheim, B. J. *Org. Lett.*, **2020**, *22*, 7261–7266.

43. Dolenc, D.; Plesničar, B. *J. Org. Chem.*, **2006**, *71*, 8028–8036.

44. Fujihira, Y.; Liang, Y.; Ono, M.; Hirano, K.; Kagawa, T.; Shibata, N. *Beilstein J. Org. Chem.*, **2021**, *17*, 431–438.

45. Pisella, G.; Gagnebin, A.; Waser, J. *Chem. - Eur. J.*, **2020**, *26*, 10199–10204.

46. Chai, J.; Ding, W.; Wu, J.; Yoshikai, N. *Chem. – Asian J.*, **2020**, *15*, 2166–2169.

47. Blake, A. J.; Novak, A.; Davies, M.; Robinson, R. I.; Woodward, S. *Synth. Commun.*, **2009**, *39*, 1065–1075.

48. Ding, W.; Wang, C.; Tan, J. R.; Ho, C. C.; León, F.; García, F.; Yoshikai, N. *Chem. Sci.*, **2020**, *11*, 7356–7361.

49. He, H.; Zhu, X. *Org. Lett.*, **2014**, *16*, 3102-3105.

50. Bruker, Bruker Inc., Madison, Wisconsin, USA, Version 6.10 edn., 1998-2000.

51. Dolomanov, O. V.; Bourhis, L. J.; Gildea, R. J.; Howard, J. A. K.; Puschmann, H. *J. Appl. Crystallogr.*, **2009**, *42*, 339–341.

52. Sheldrick, G. M. *Acta Crystallogr. Sect. Found. Adv.*, **2015**, *71*, 3–8.

53. Sheldrick, G. M. *Acta Crystallogr. Sect. C Struct. Chem.*, **2015**, *71*, 3–8.

^{1}H, ^{13}C and ^{19}F NMR spectra

^{1}H NMR spectrum of 2,6-dimethyliodobenzene **S2** – $CDCl_3$

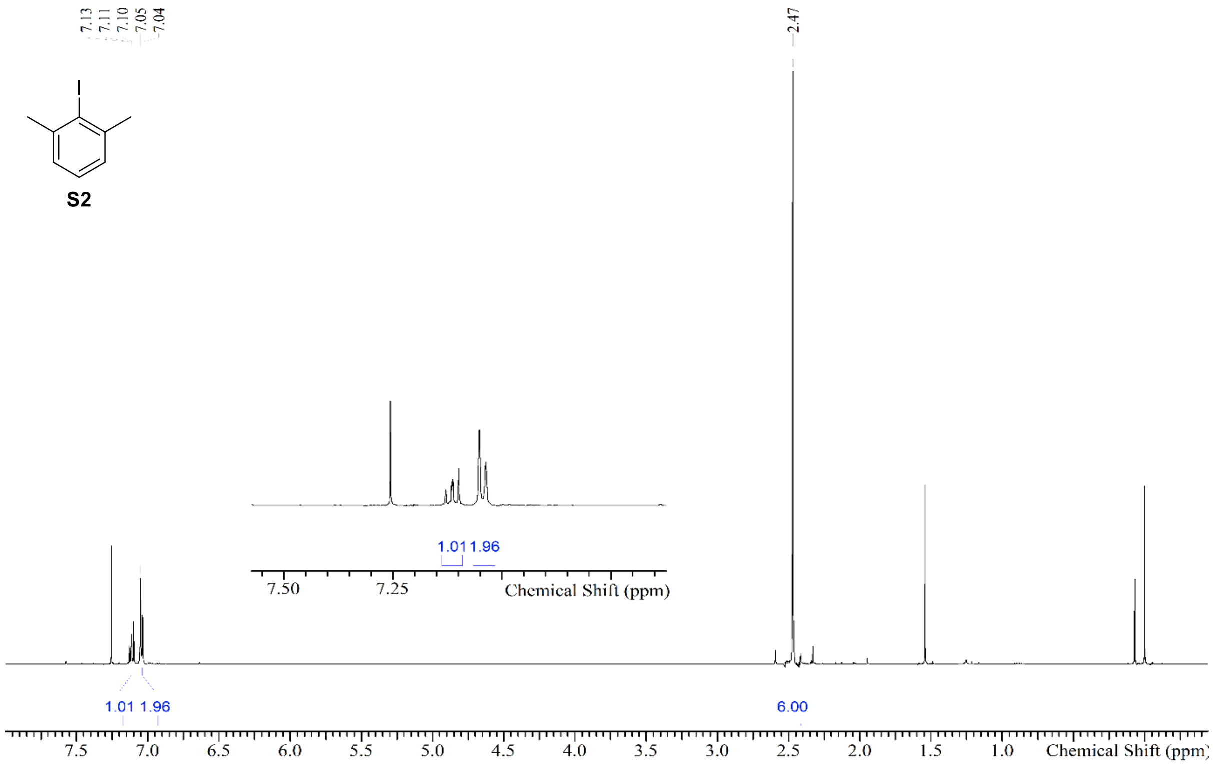

^{13}C NMR spectrum for 2,6-dimethyliodobenzene **S2** - $CDCl_3$

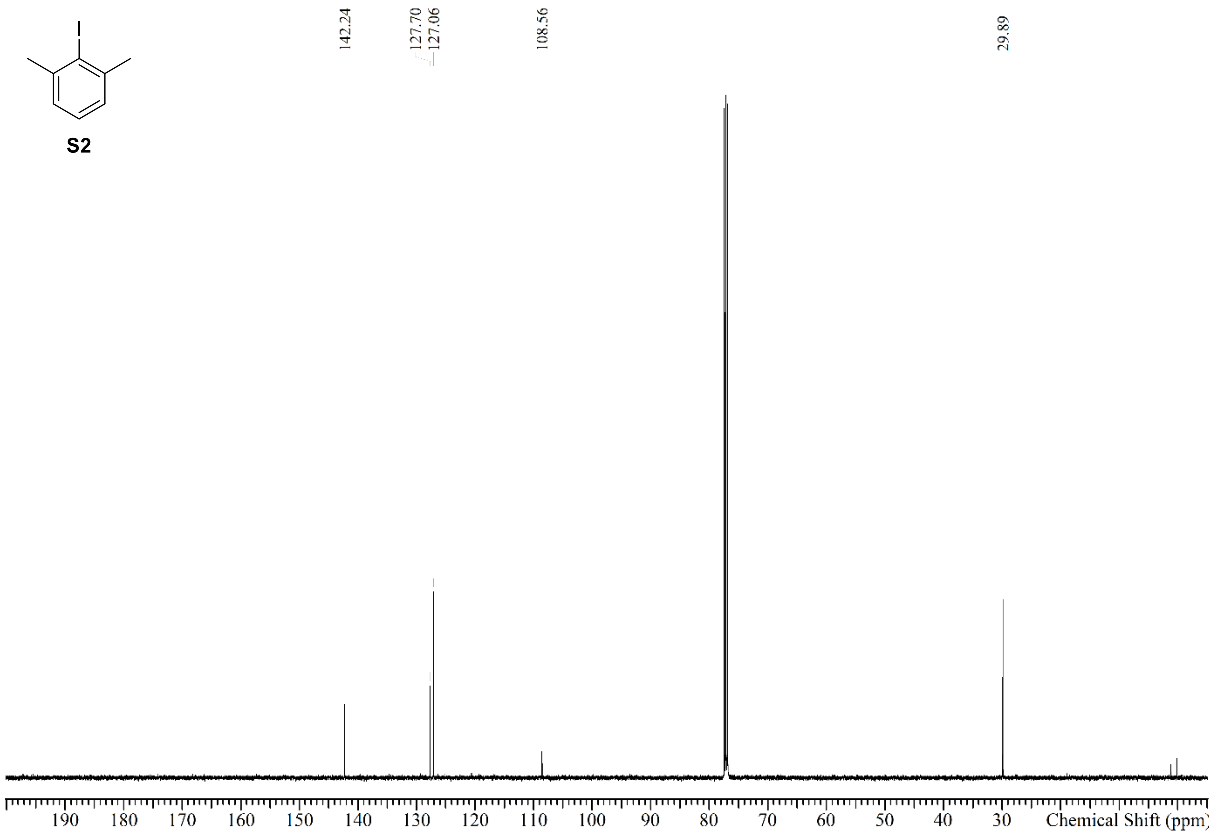

^{1}H NMR spectrum of 2-iodoisophthalic acid **S3** – d_6-DMSO

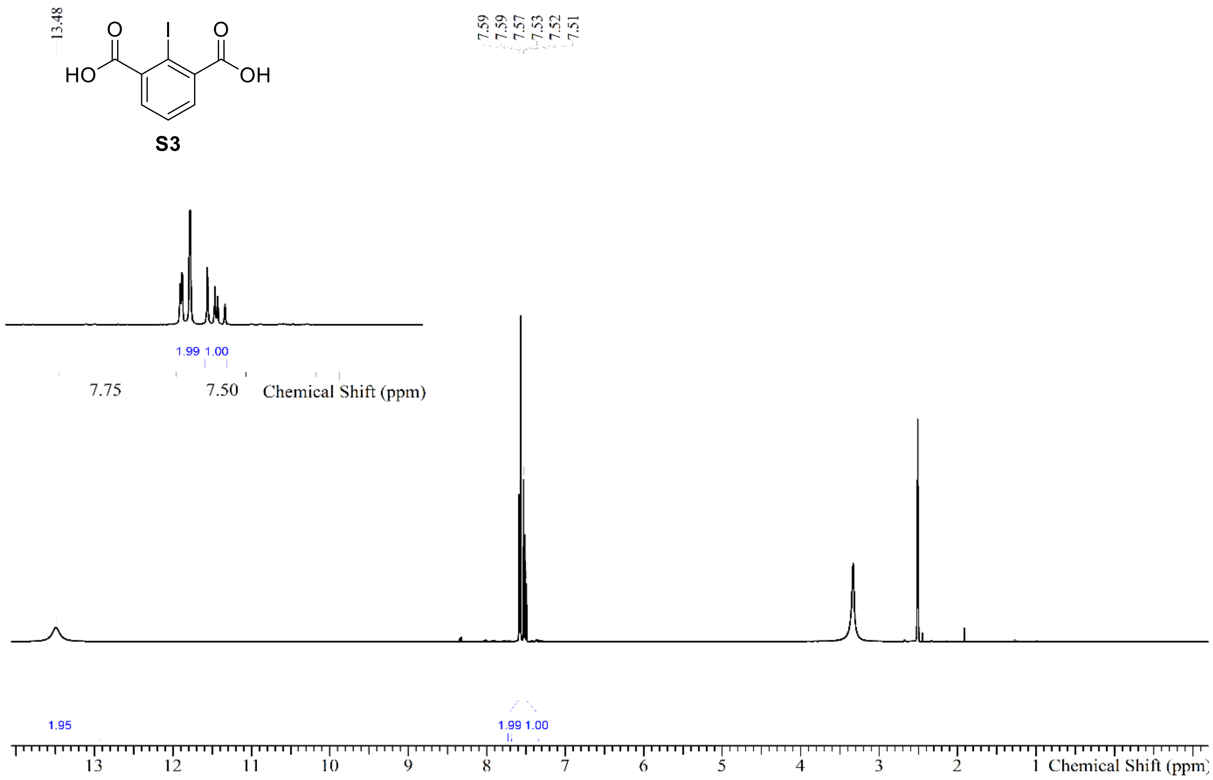

^{13}C NMR spectrum of 2-iodoisophthalic acid **S3** – d_6-DMSO

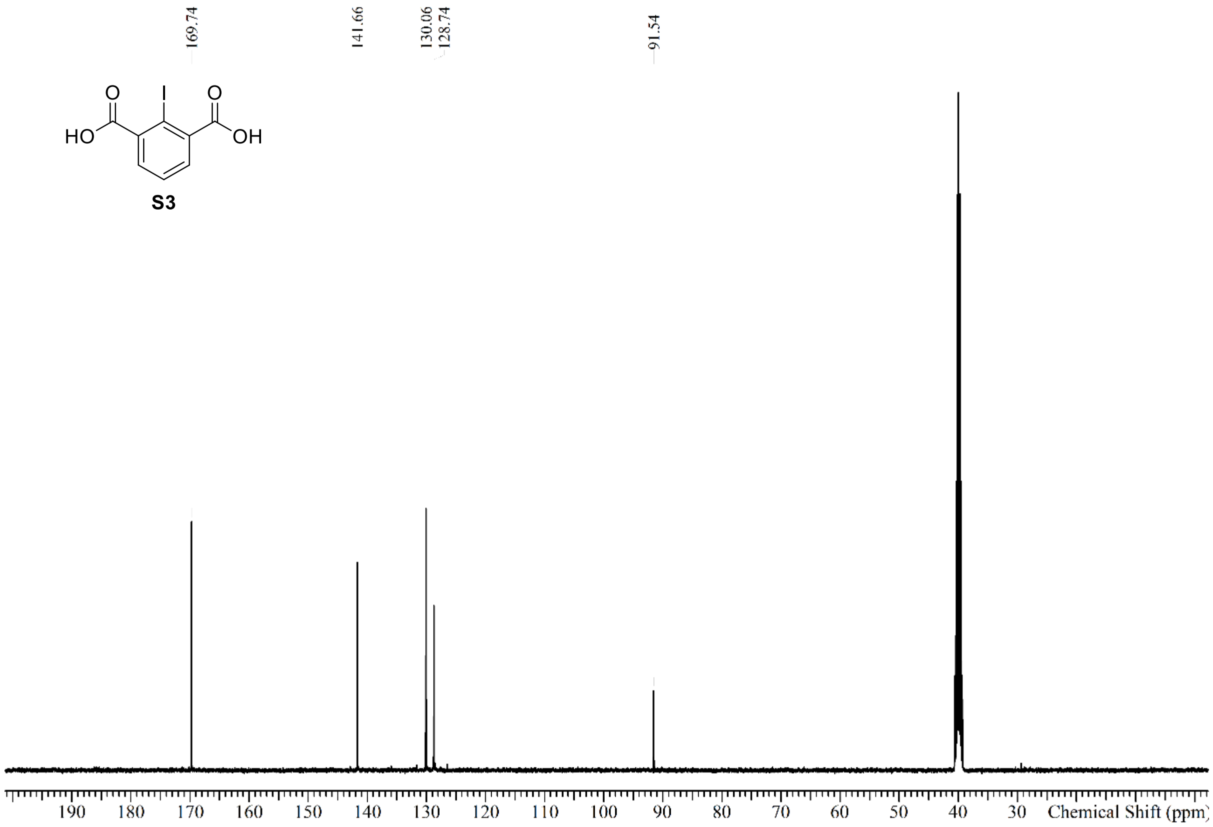

^{1}H NMR spectrum of dimethyl 2-iodoisophthalate **S4** – $CDCl_3$

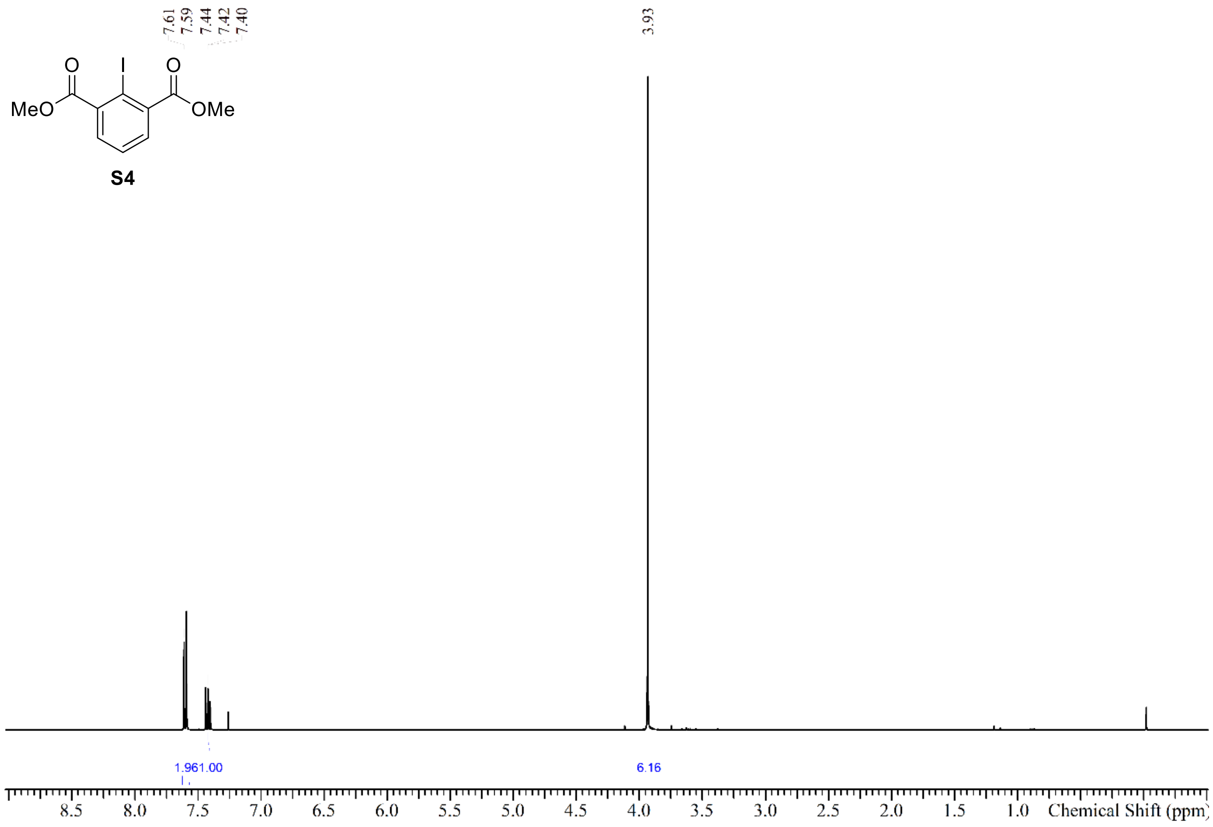

^{13}C NMR spectrum of dimethyl 2-iodoisophthalate **S4** – $CDCl_3$

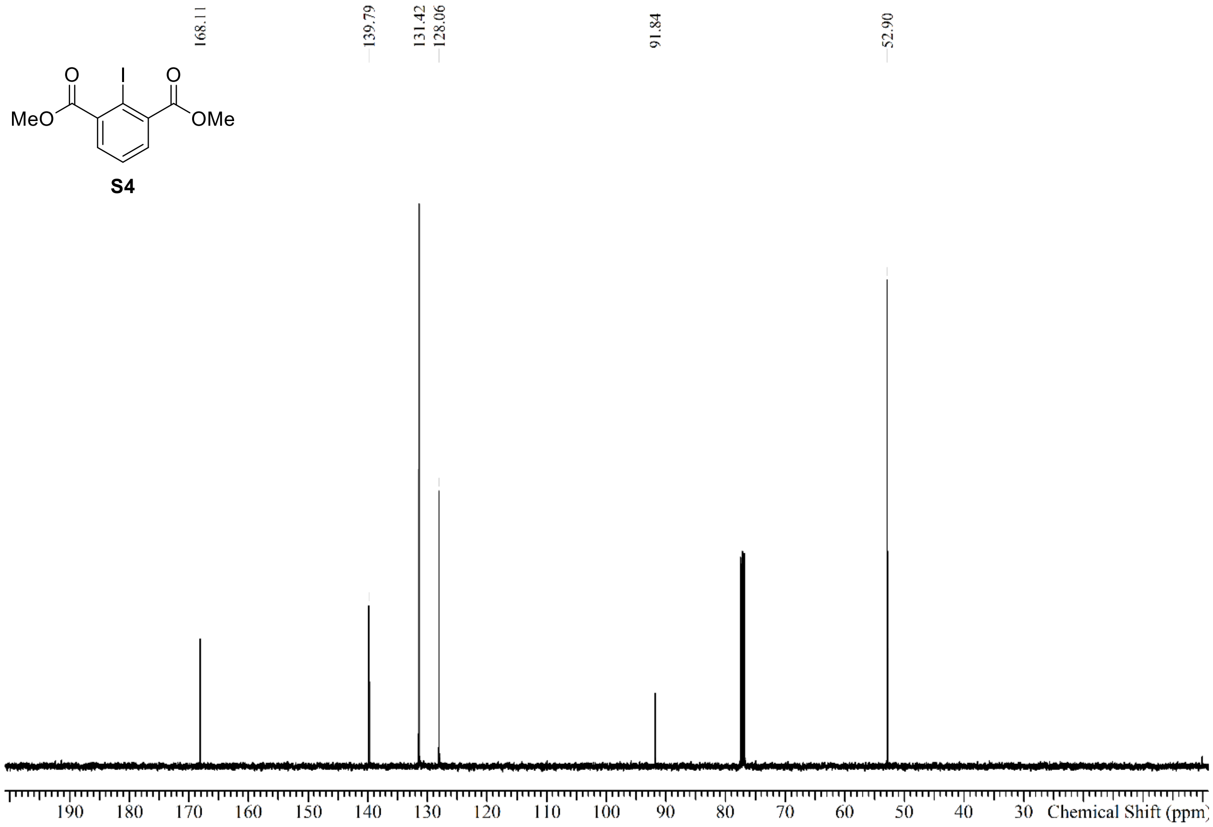

^{1}H NMR spectrum of iodo-diol **8** – $CDCl_3$

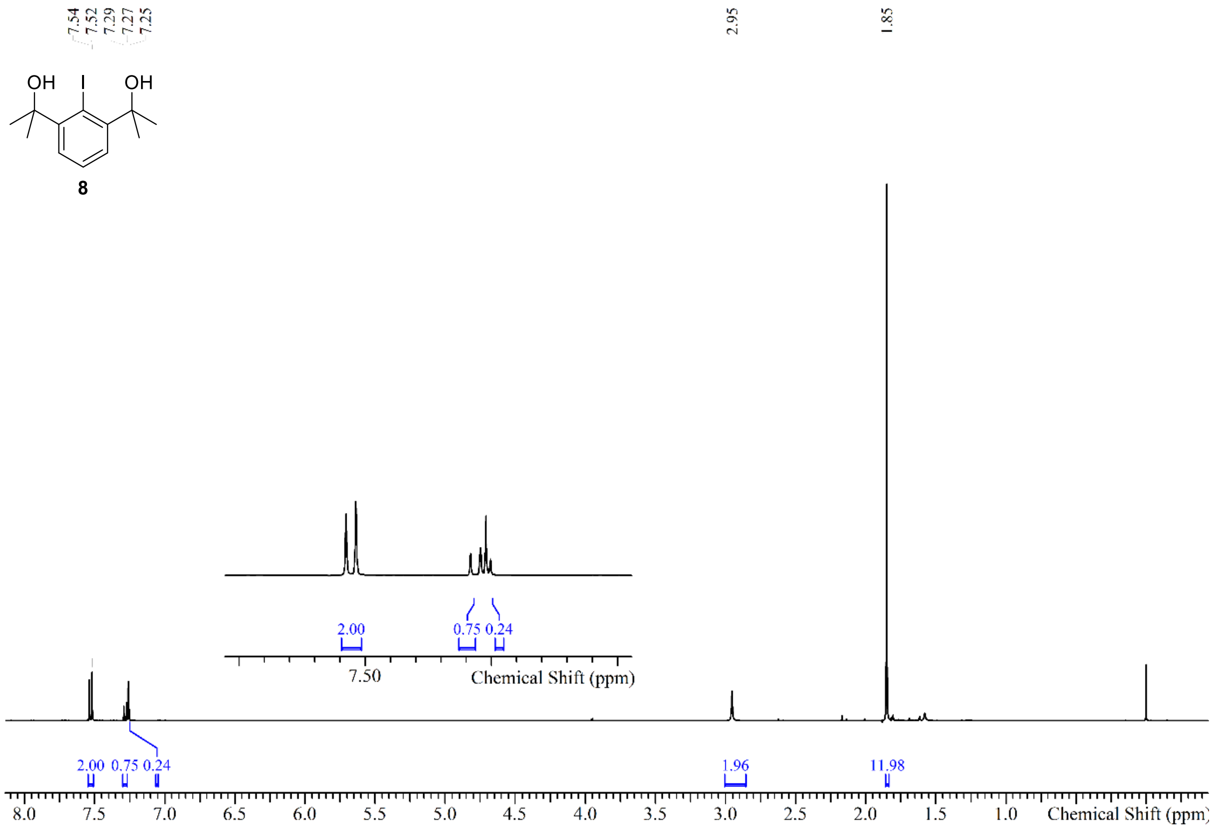

^{13}C NMR spectrum of iodo-diol **8** – $CDCl_3$

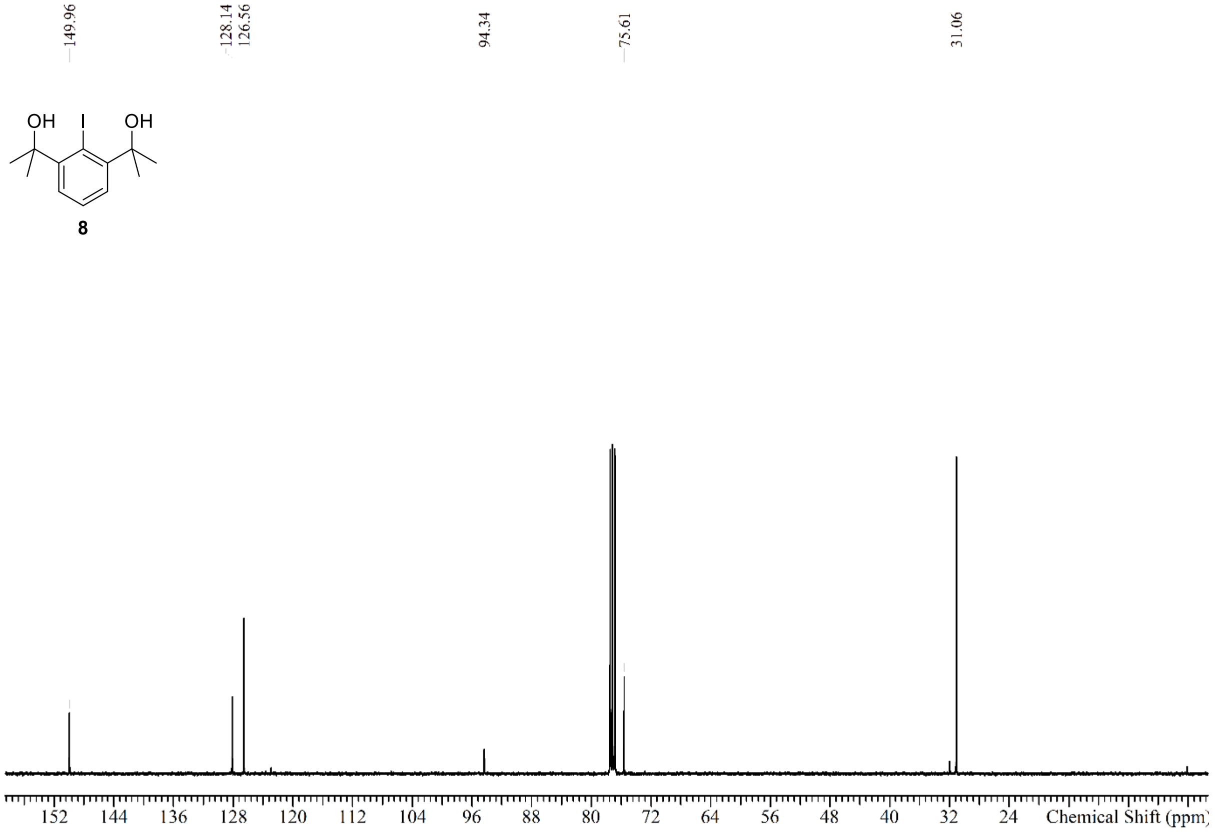

^{1}H NMR spectrum of dioxoiodane **10** – $CDCl_3$

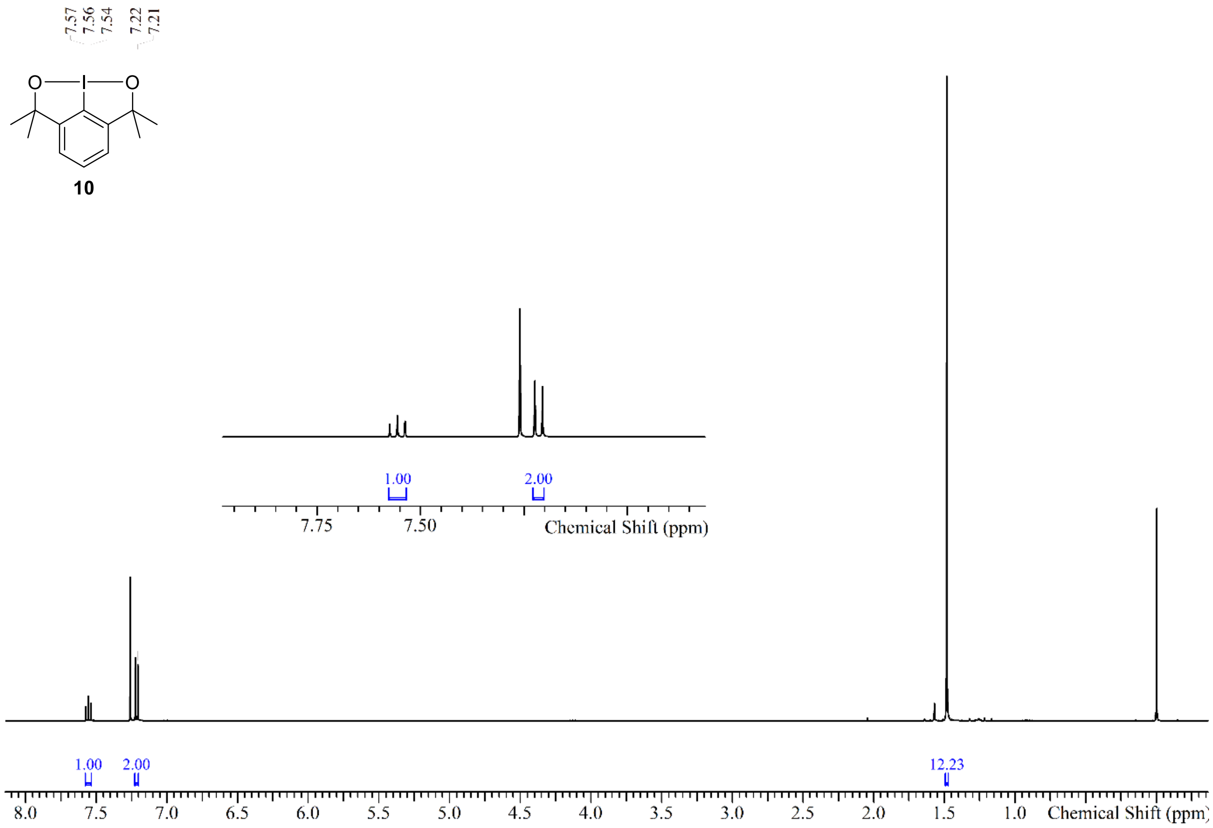

^{13}C NMR spectrum of dioxoiodane **10** – $CDCl_3$

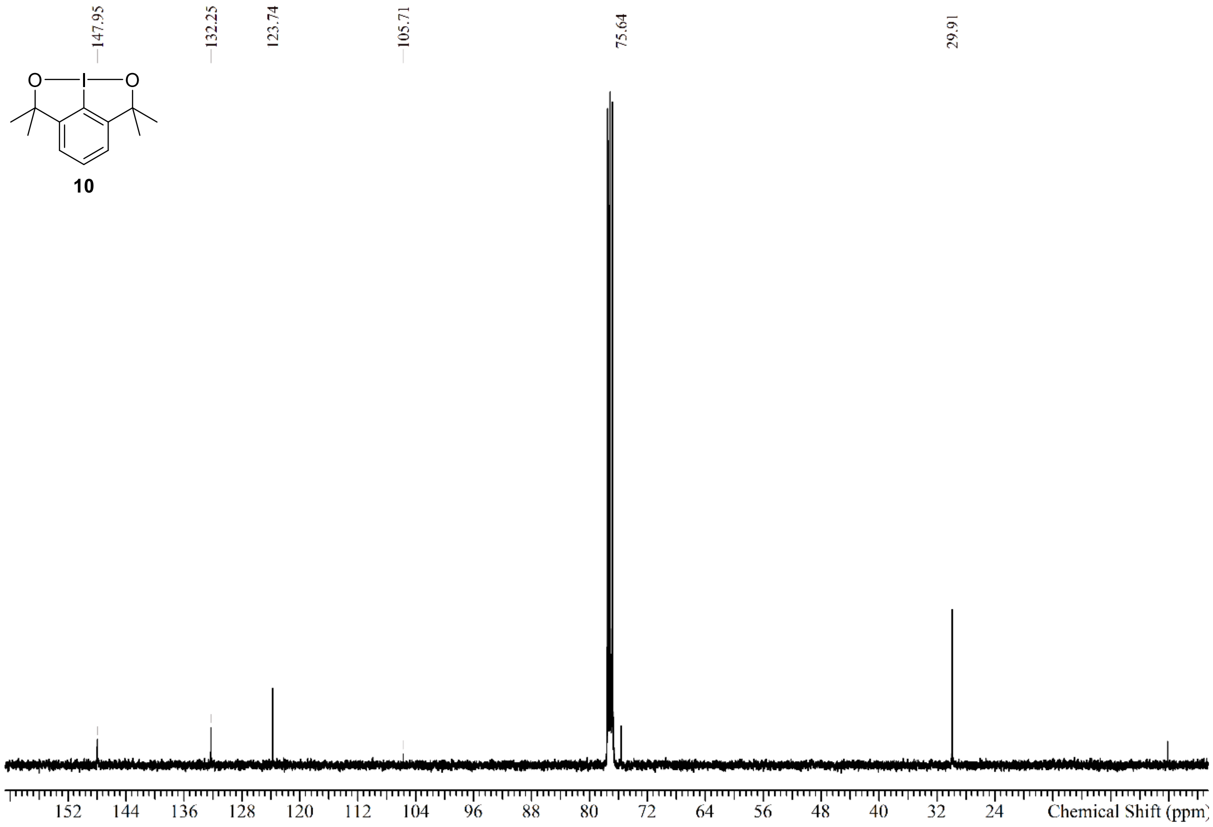

^{1}H NMR spectrum of dioxoiodane **10** - CD_3CN

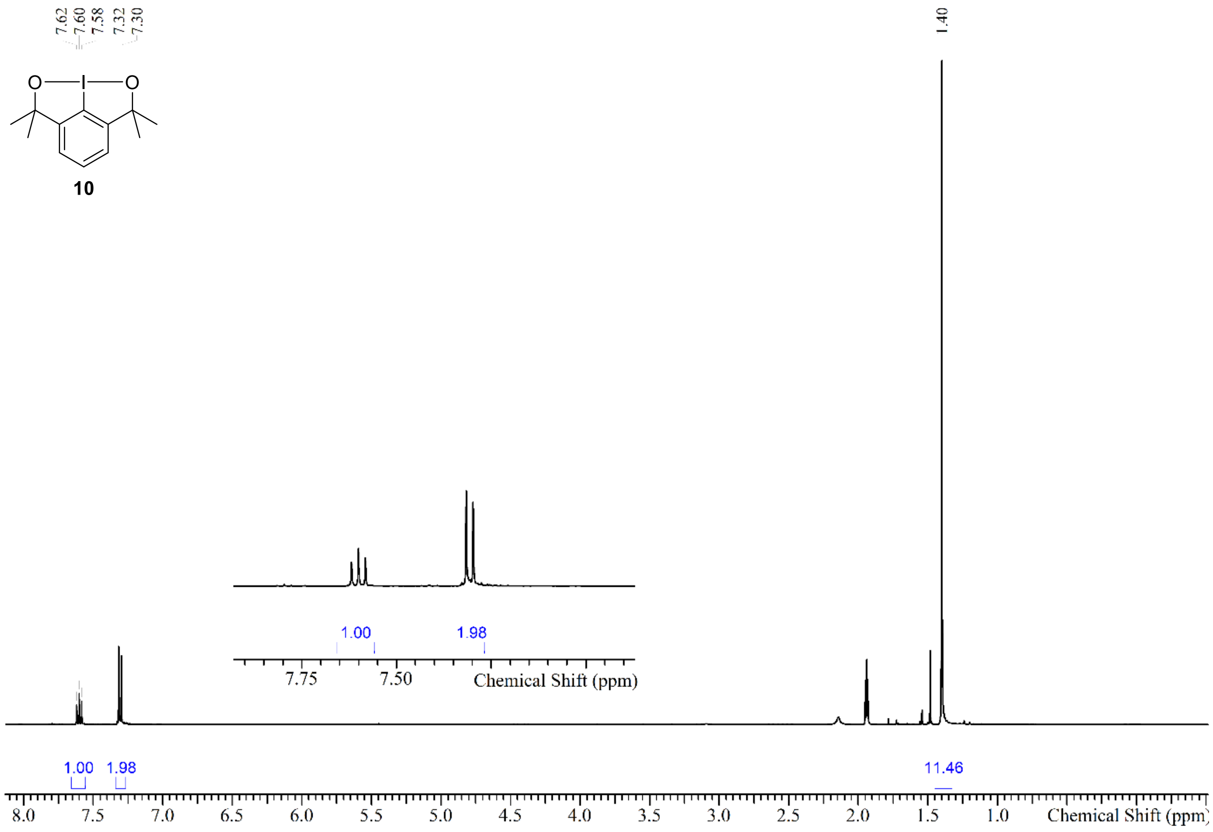

^{13}C NMR spectrum of dioxoiodane **10** - CD_3CN

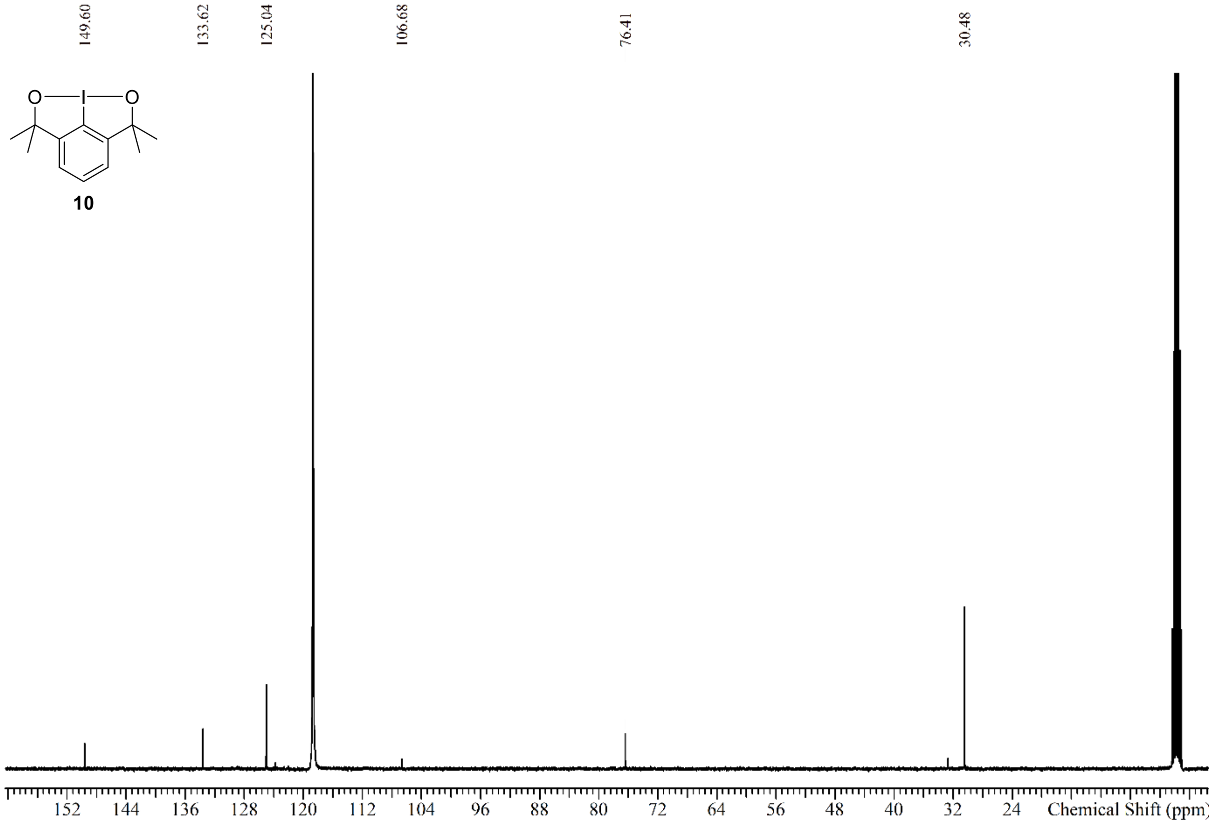

^{1}H NMR spectrum of methyl 3-(2-hydroxypropan-2-yl)benzoate **S6** – $CDCl_3$

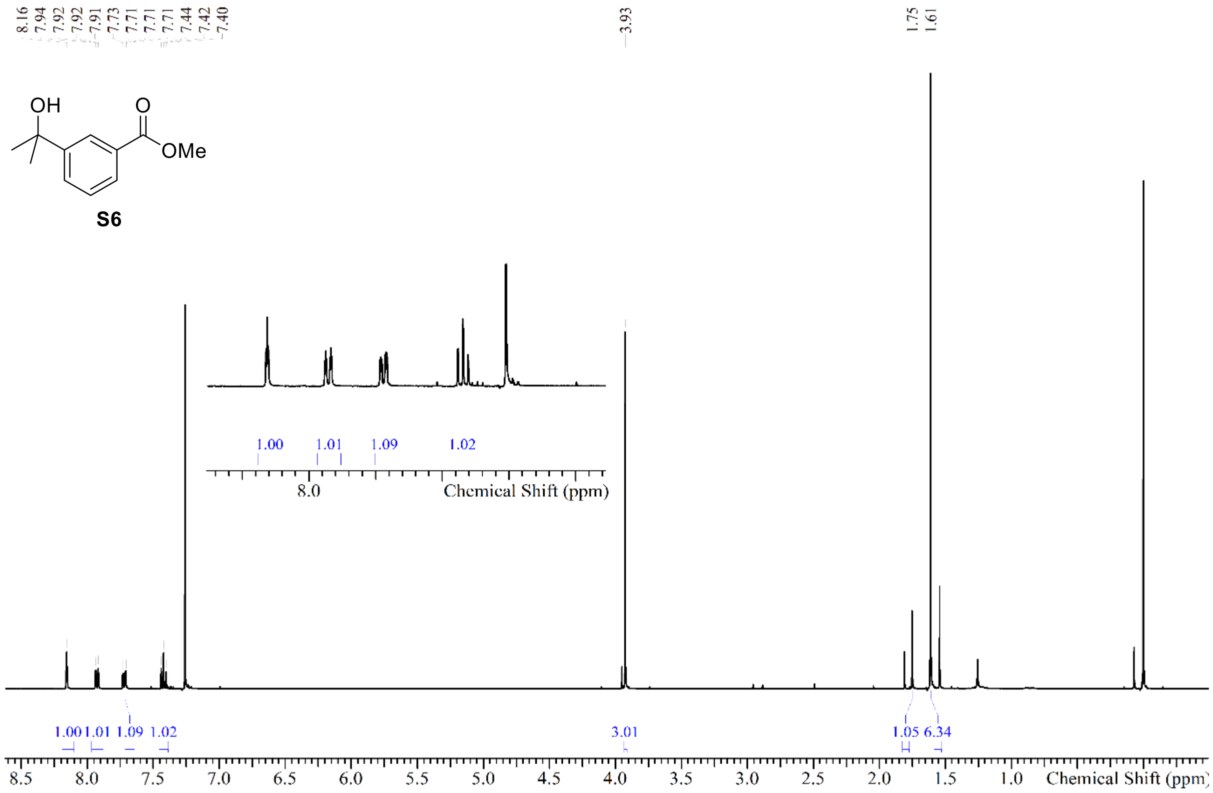

^{13}C NMR spectrum of methyl 3-(2-hydroxypropan-2-yl)benzoate **S6** – $CDCl_3$

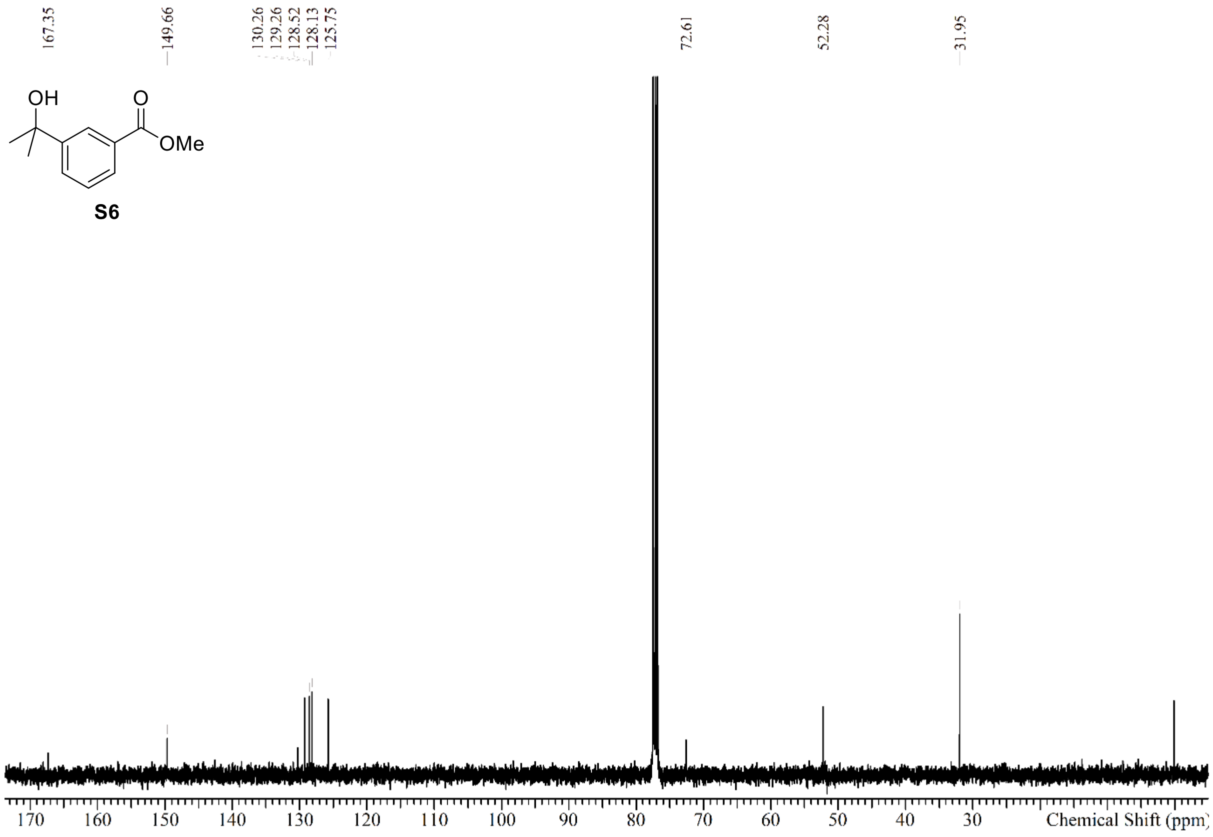

^{1}H NMR spectrum of dimethyl isophthalate **S7** – $CDCl_3$

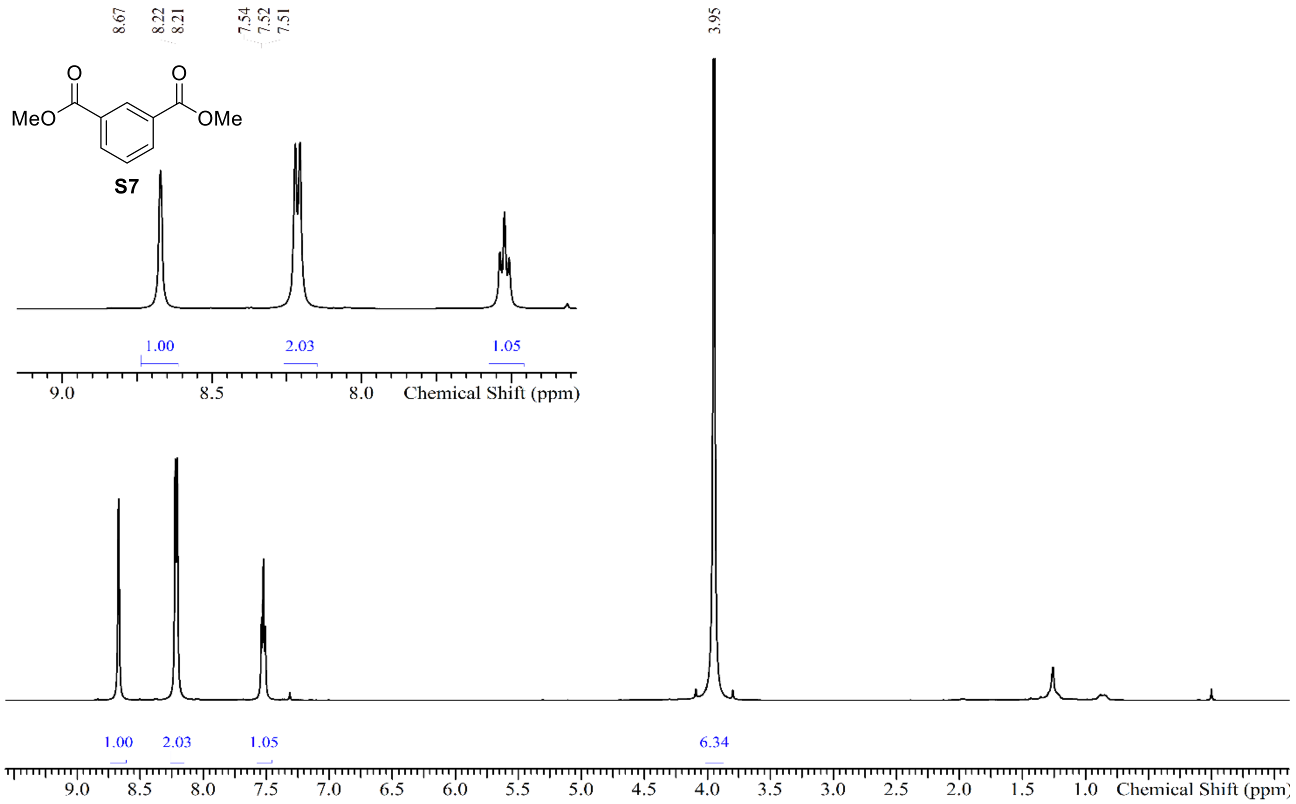

^{13}C NMR spectrum of dimethyl isophthalate **S7** – $CDCl_3$

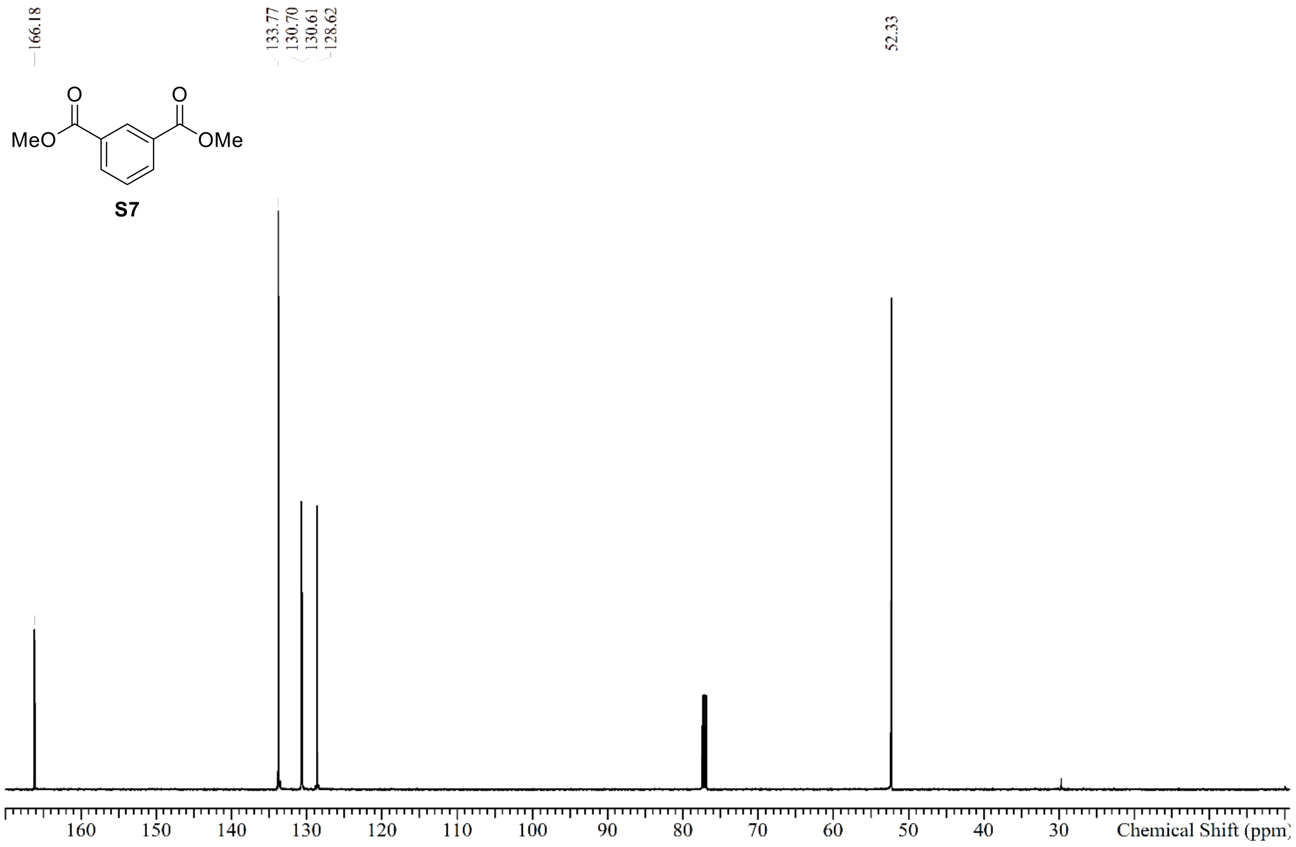

^{1}H NMR spectrum of methyl 3-(2-hydroxypropan-2-yl)-2-iodobenzoate **S5** – $CDCl_3$

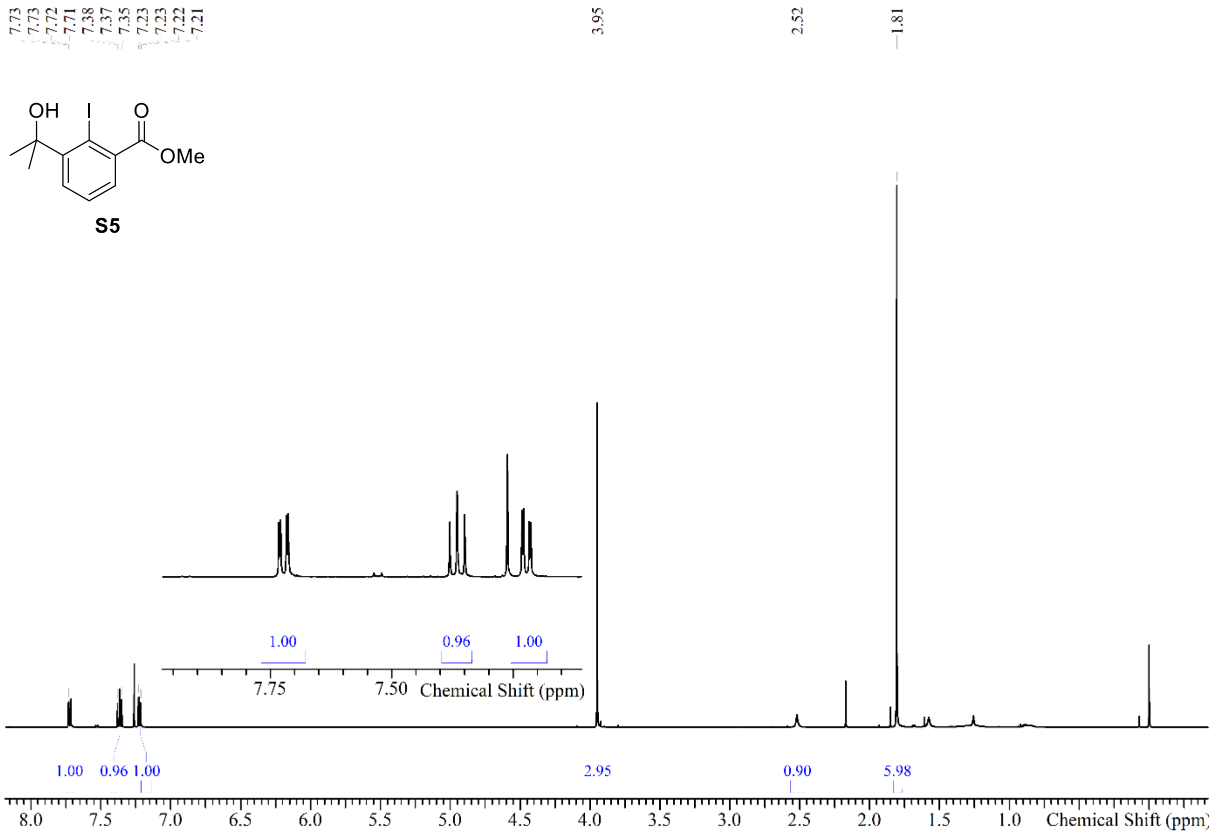

^{13}C NMR spectrum of methyl 3-(2-hydroxypropan-2-yl)-2-iodobenzoate **S5** – $CDCl_3$

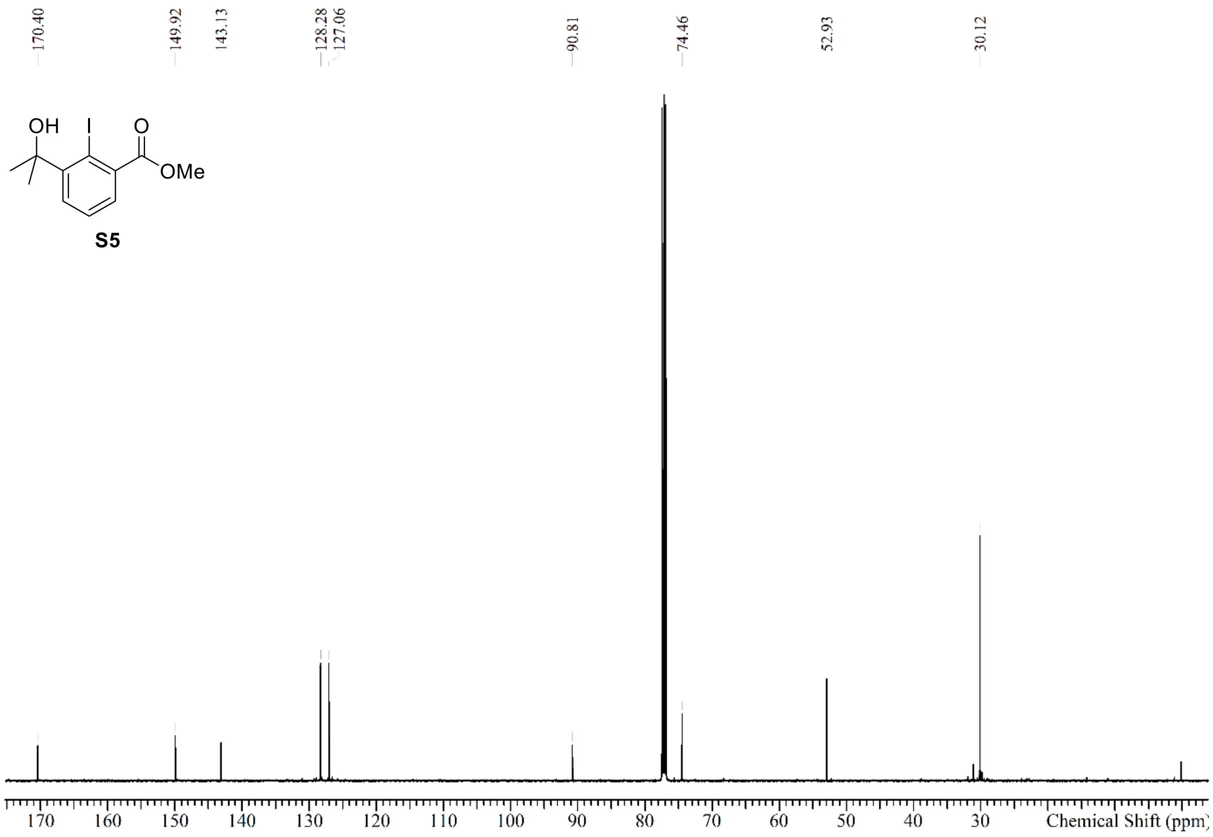

^{1}H NMR spectrum of 2,2'-(2-iodo-1,3-phenylene)bis(propan-2-ol) **S8** – $CDCl_3$

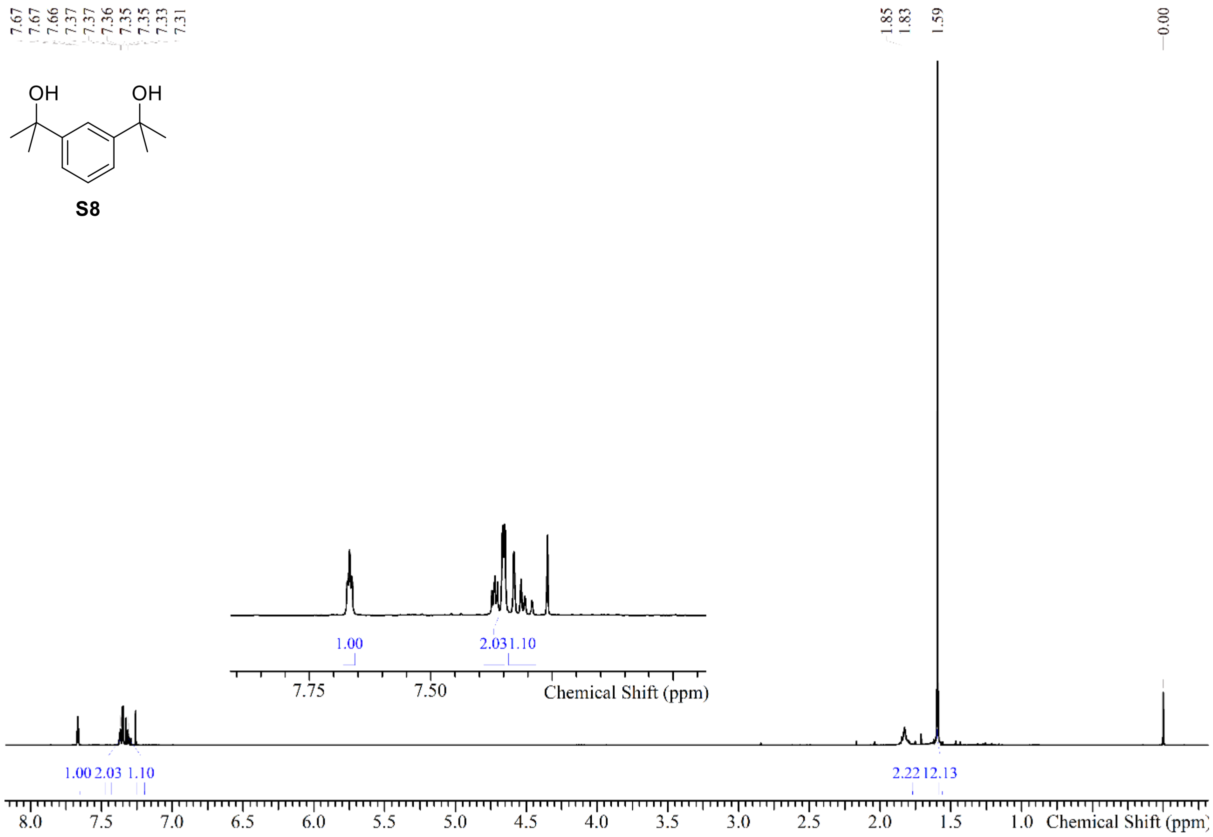

^{13}C NMR spectrum of 2,2'-(2-iodo-1,3-phenylene)bis(propan-2-ol) **S8** – $CDCl_3$

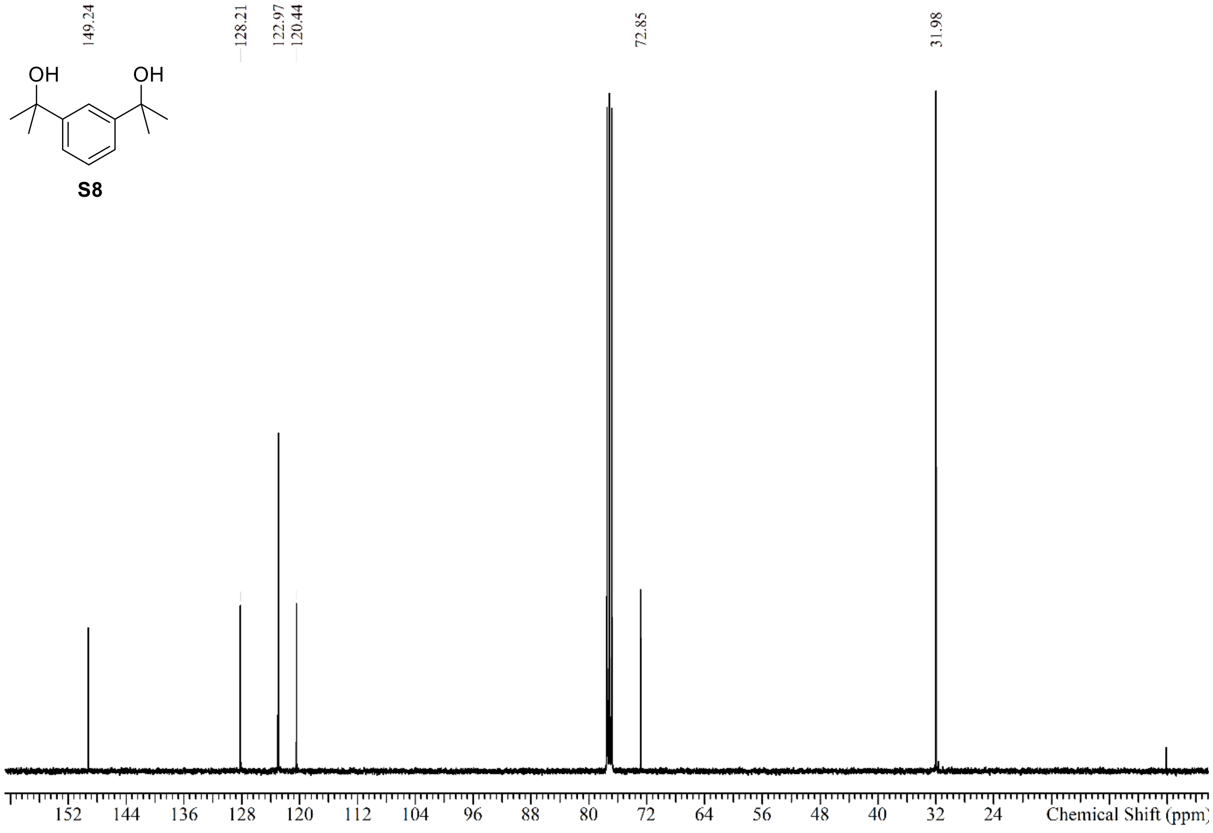

^{1}H NMR spectrum of difluoroiodane **6** – CD_3CN

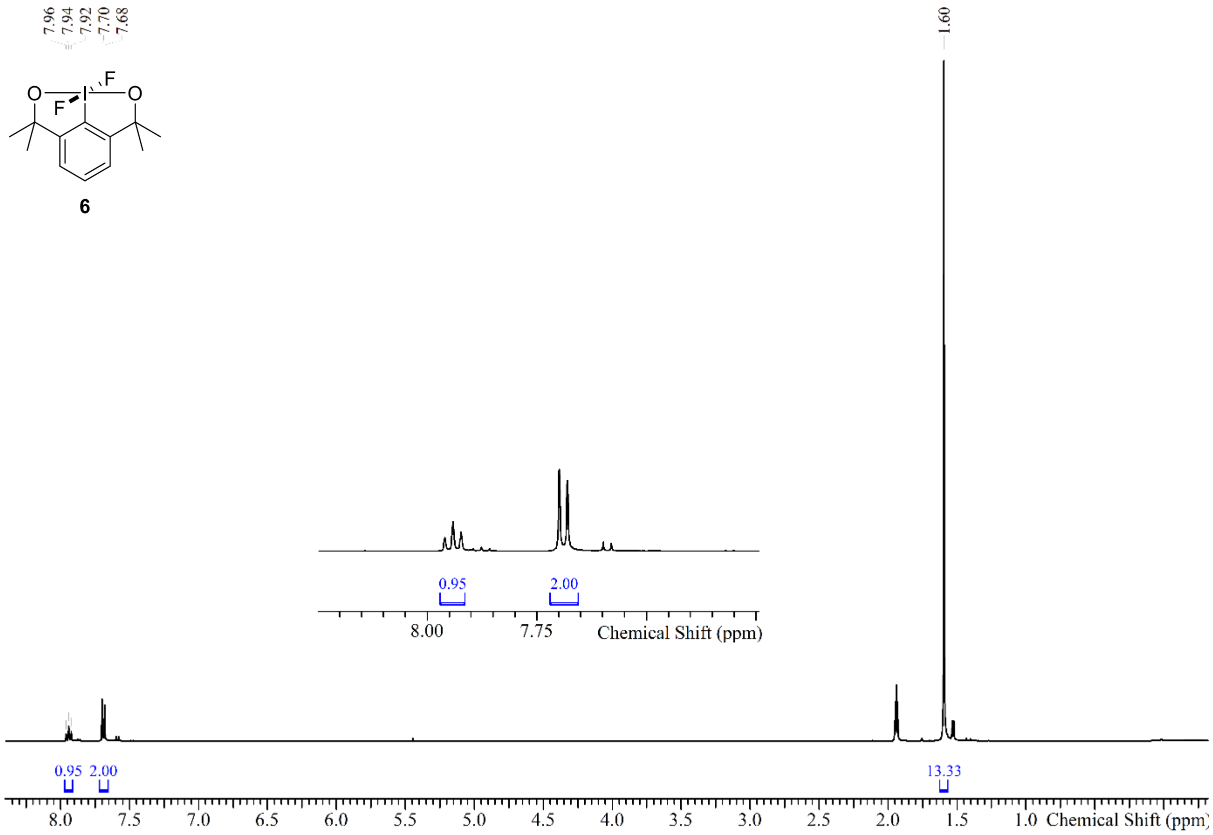

^{13}C NMR spectrum of difluoroiodane **6** – CD_3CN

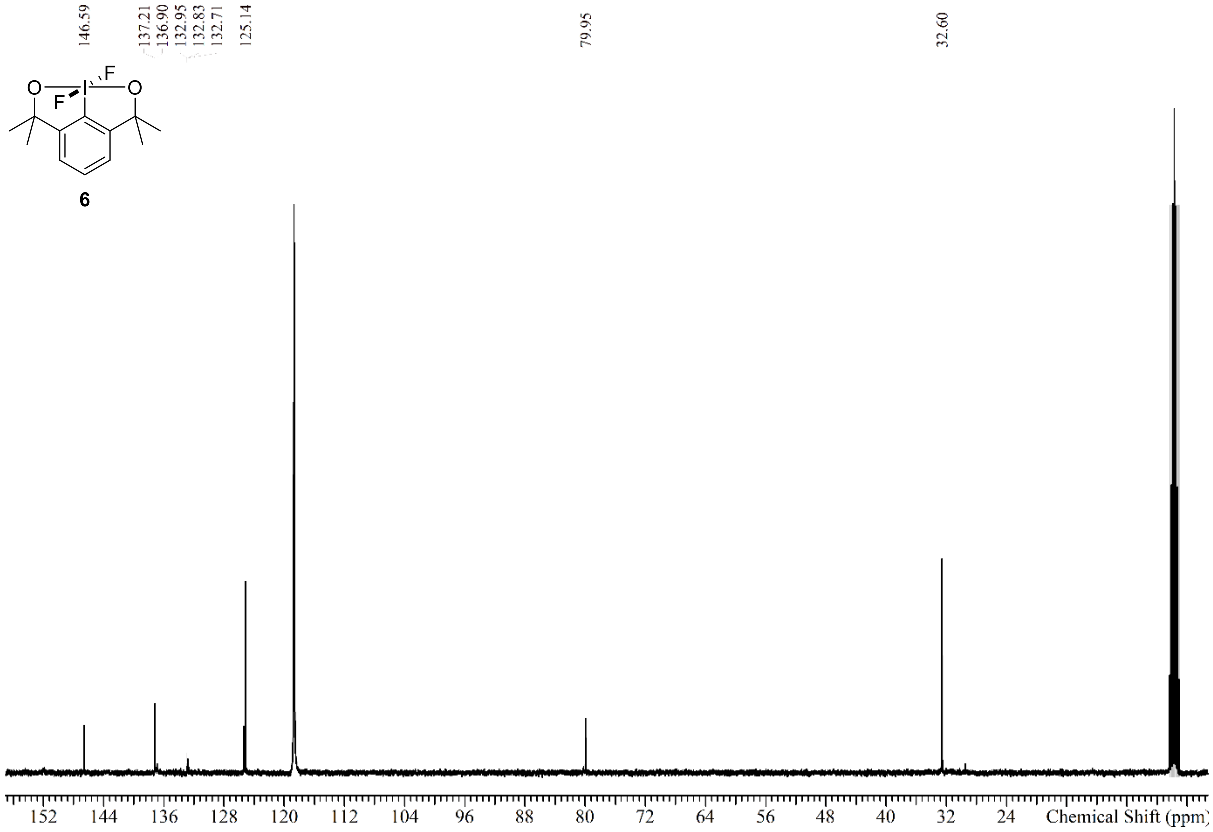

^{19}F NMR spectrum of difluoroiodane **6** – CD_3CN

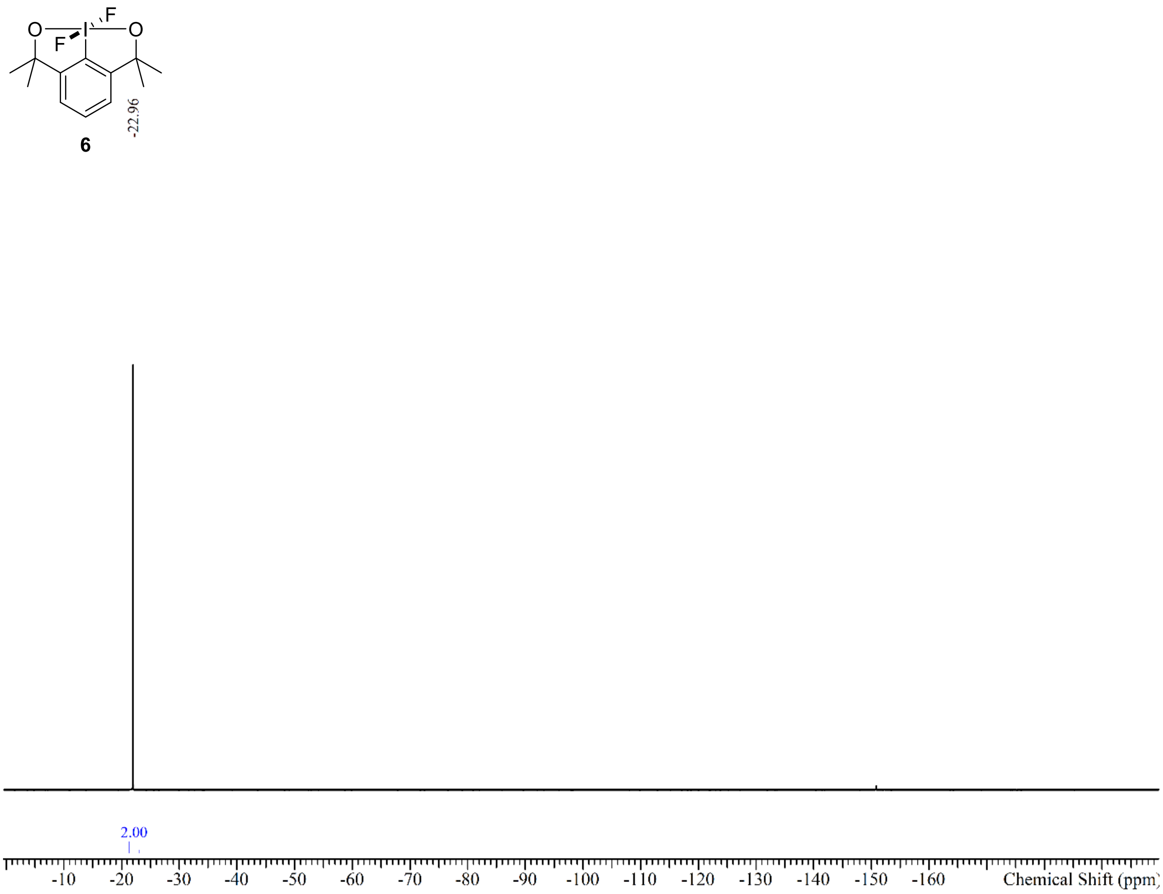

^{1}H NMR spectrum of iodosyl **9** – CD_3CN

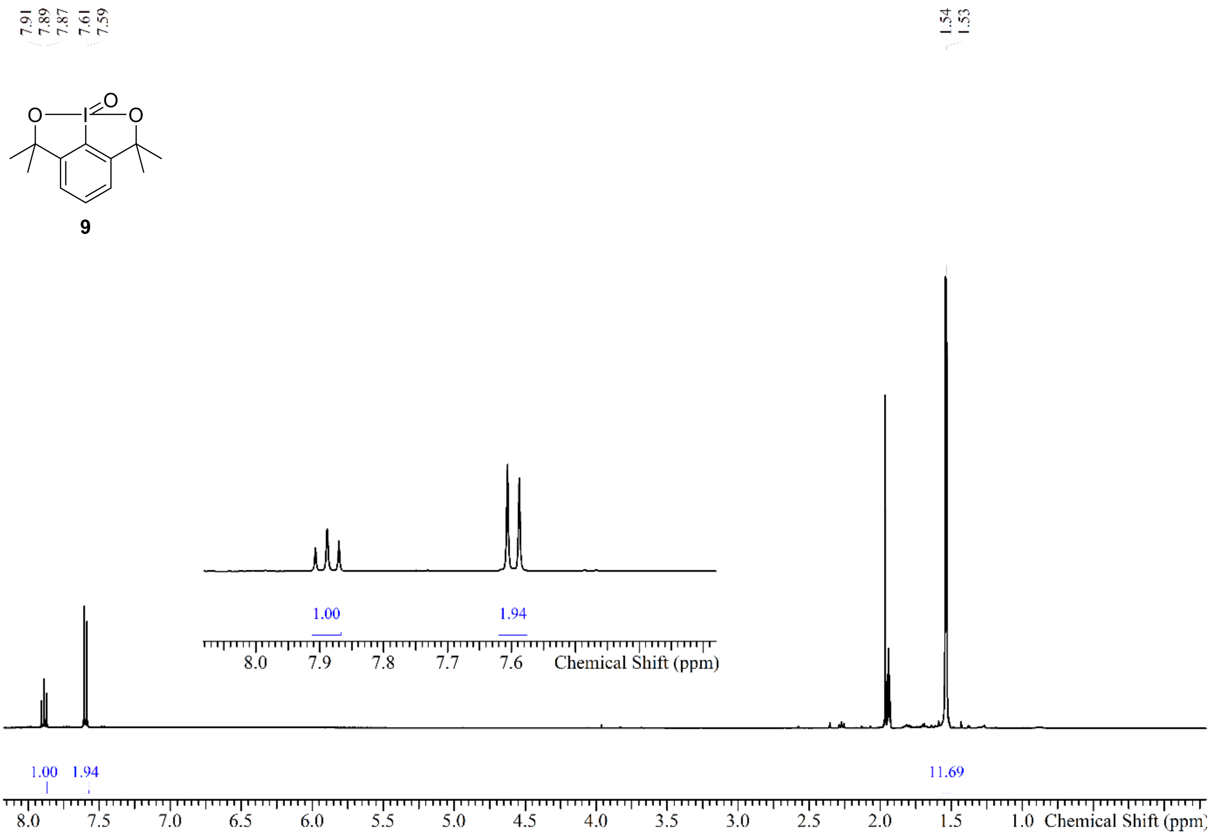

^{13}C NMR spectrum of iodosyl **9** – CD_3CN

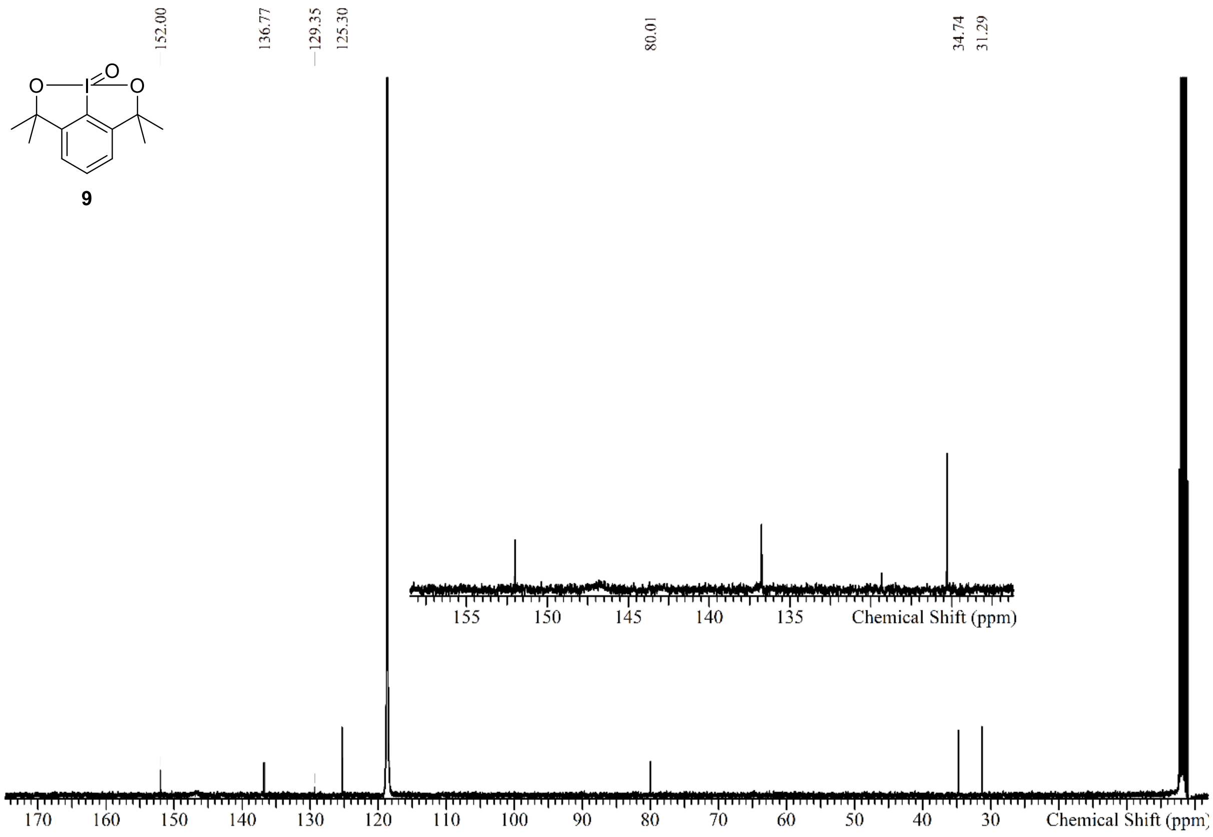

^{1}H NMR spectrum of dibenzylamide **S9a** – d_6-DMSO

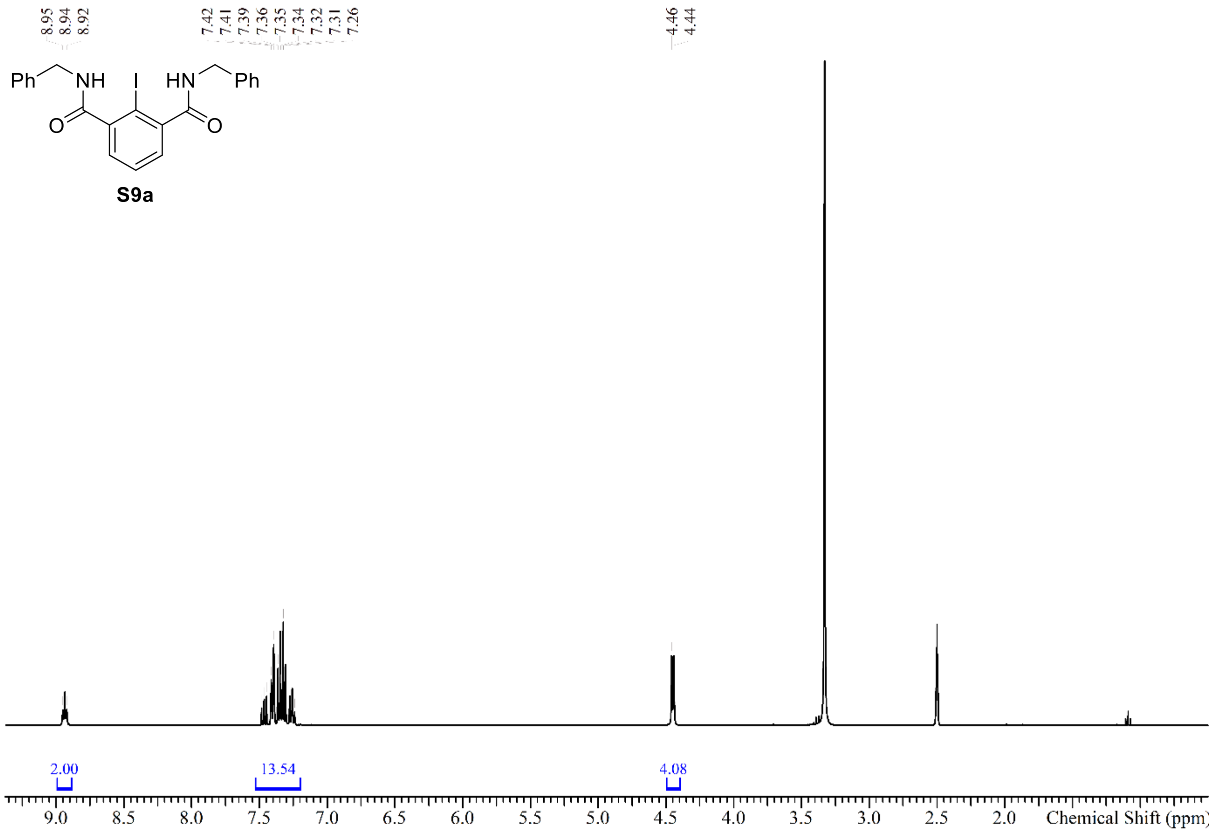

^{13}C NMR spectrum of dibenzylamide **S9a** – d$_6$-DMSO

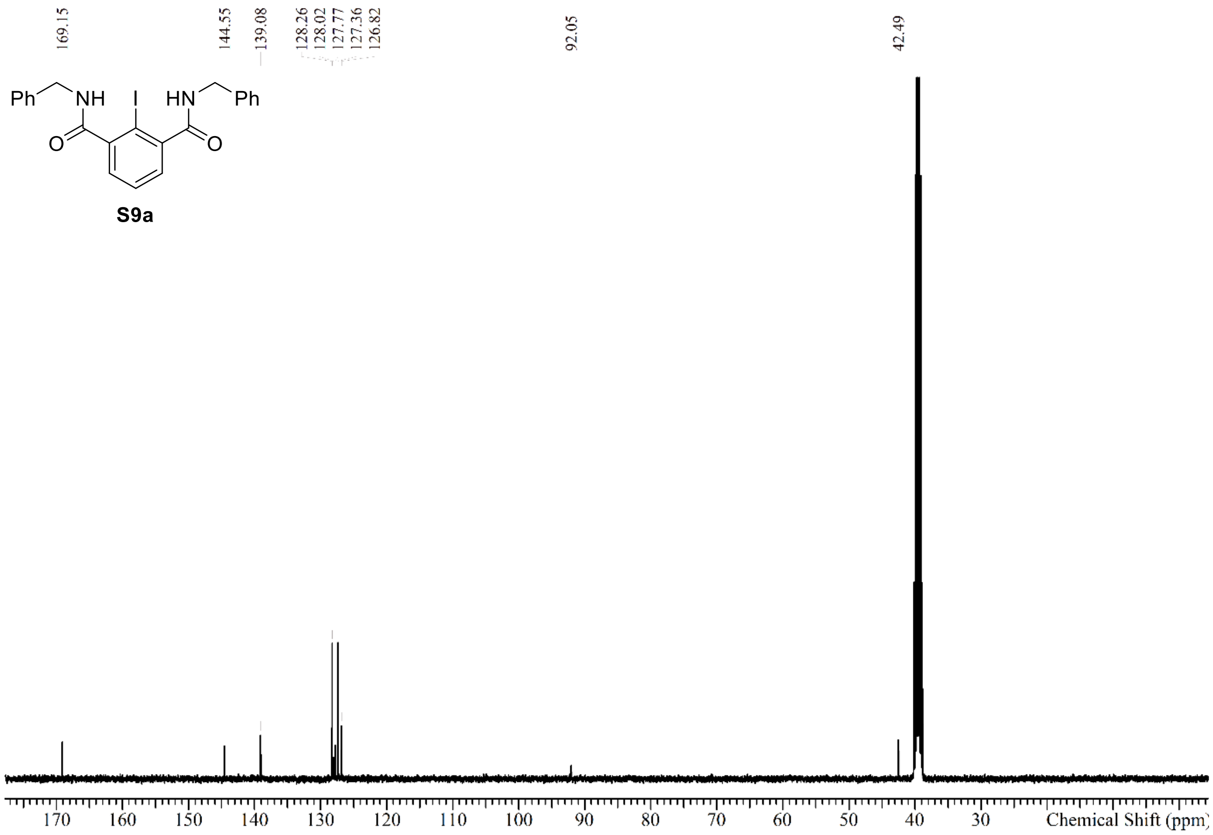

^{1}H NMR spectrum of bis(isopropyl)amidoiodobenzene **S9b** – CDCl$_3$

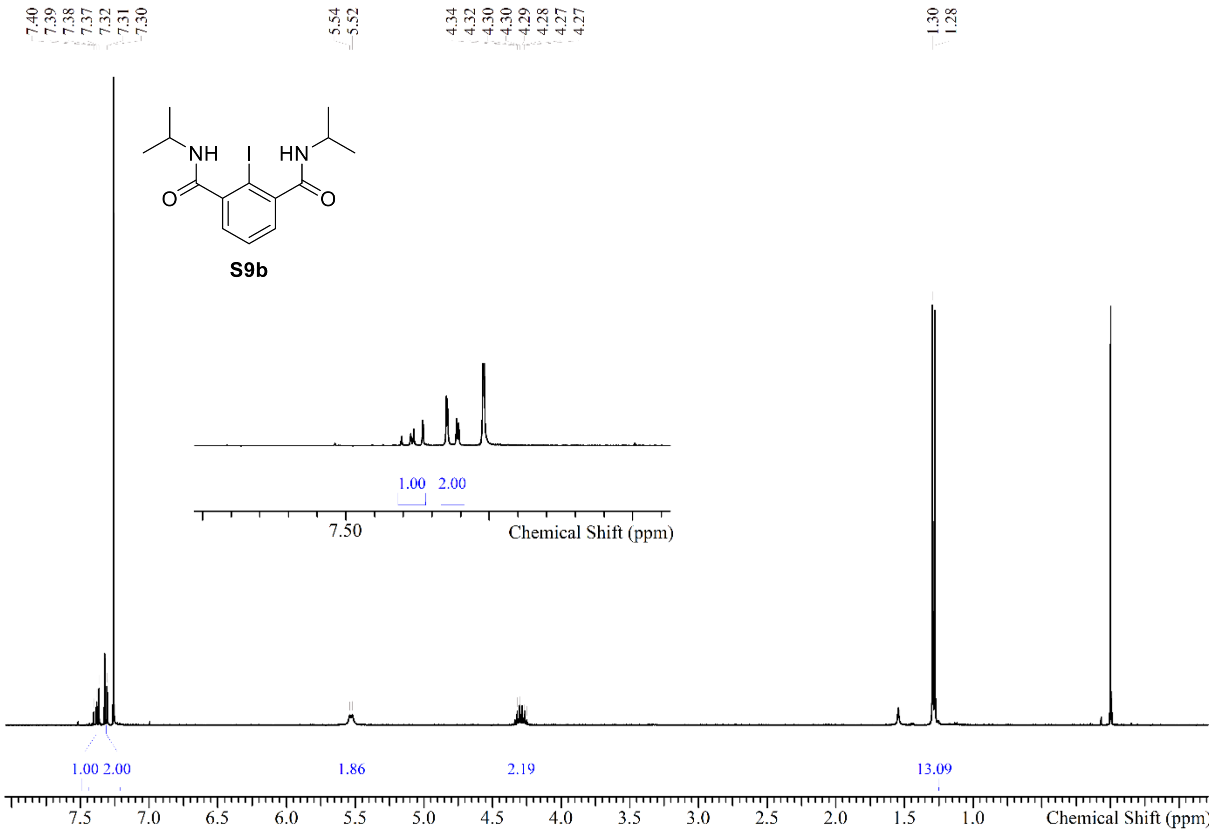

^{13}C NMR spectrum of bis(isopropyl)amidoiodobenzene **S9b** – $CDCl_3$

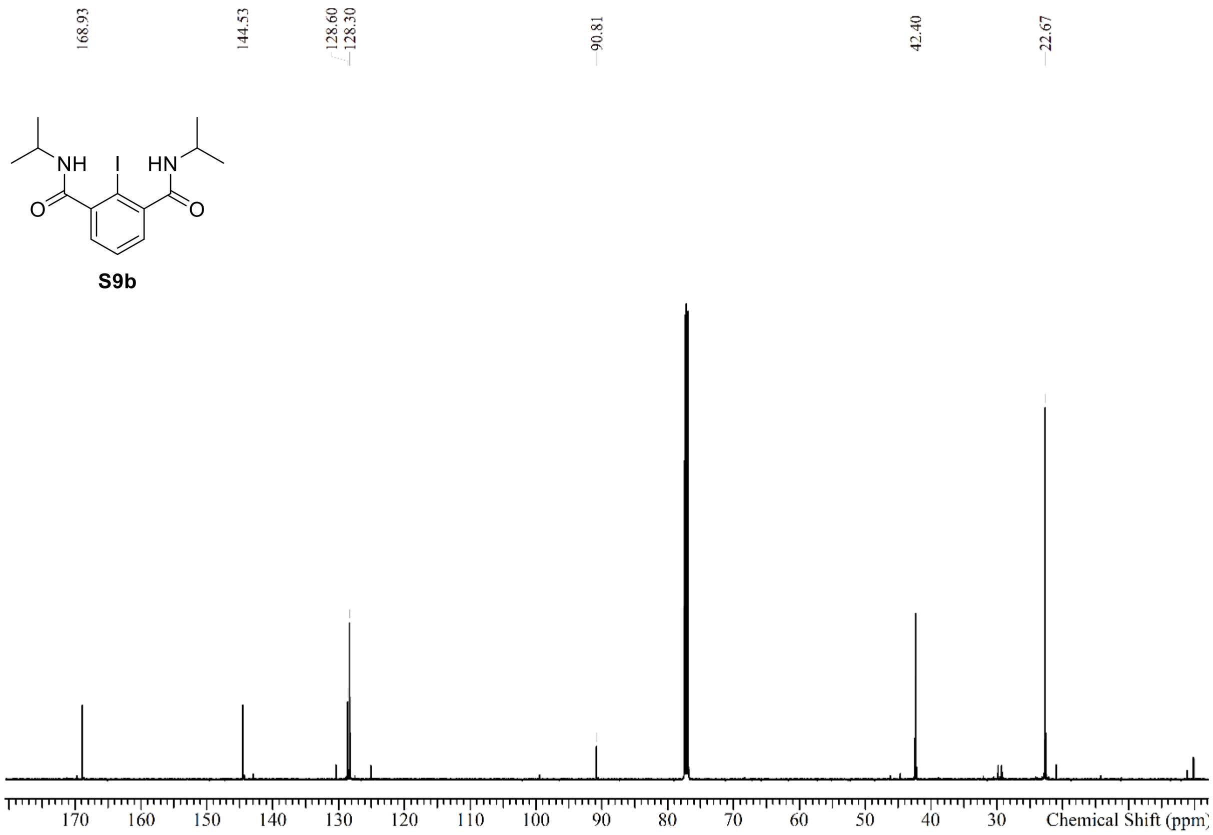

^{1}H NMR spectrum of dibenzyliodane **11a** – CD_3CN

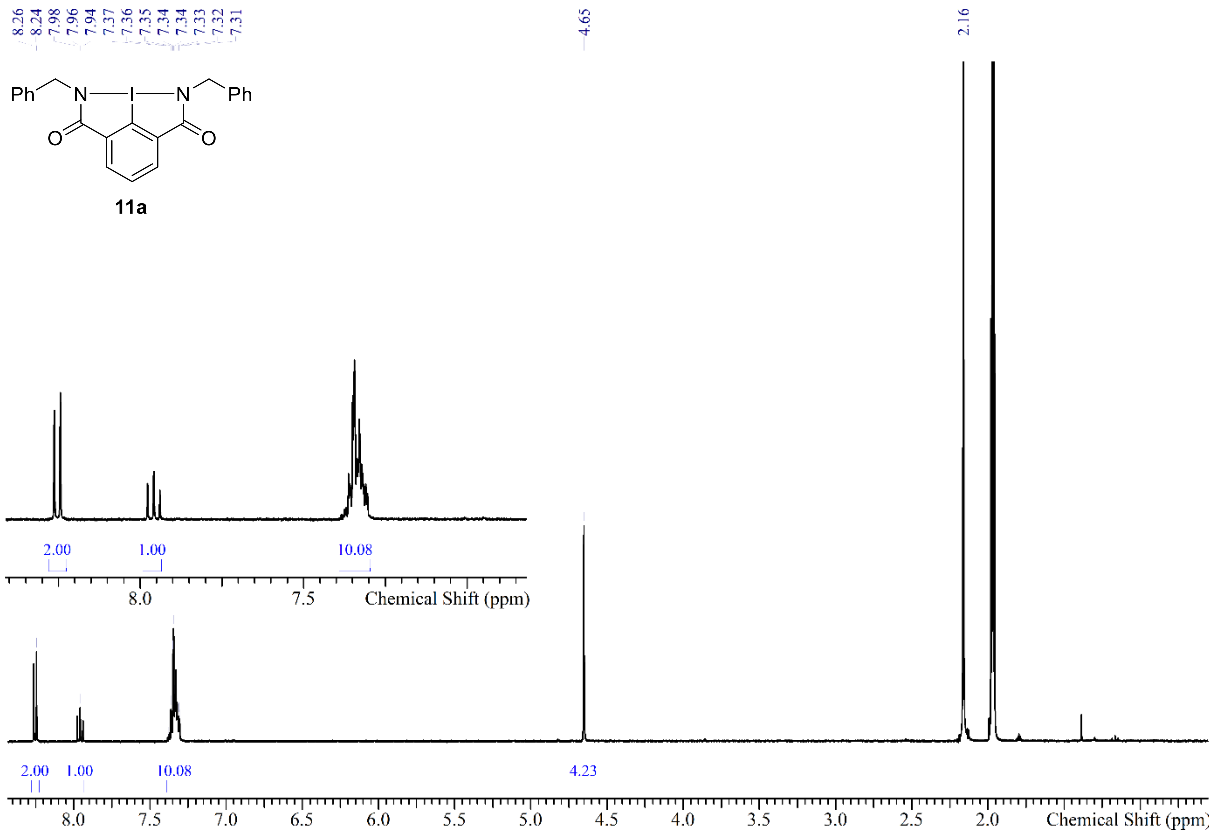

^{13}C NMR spectrum of dibenzyliodane **11a** – CD_3CN

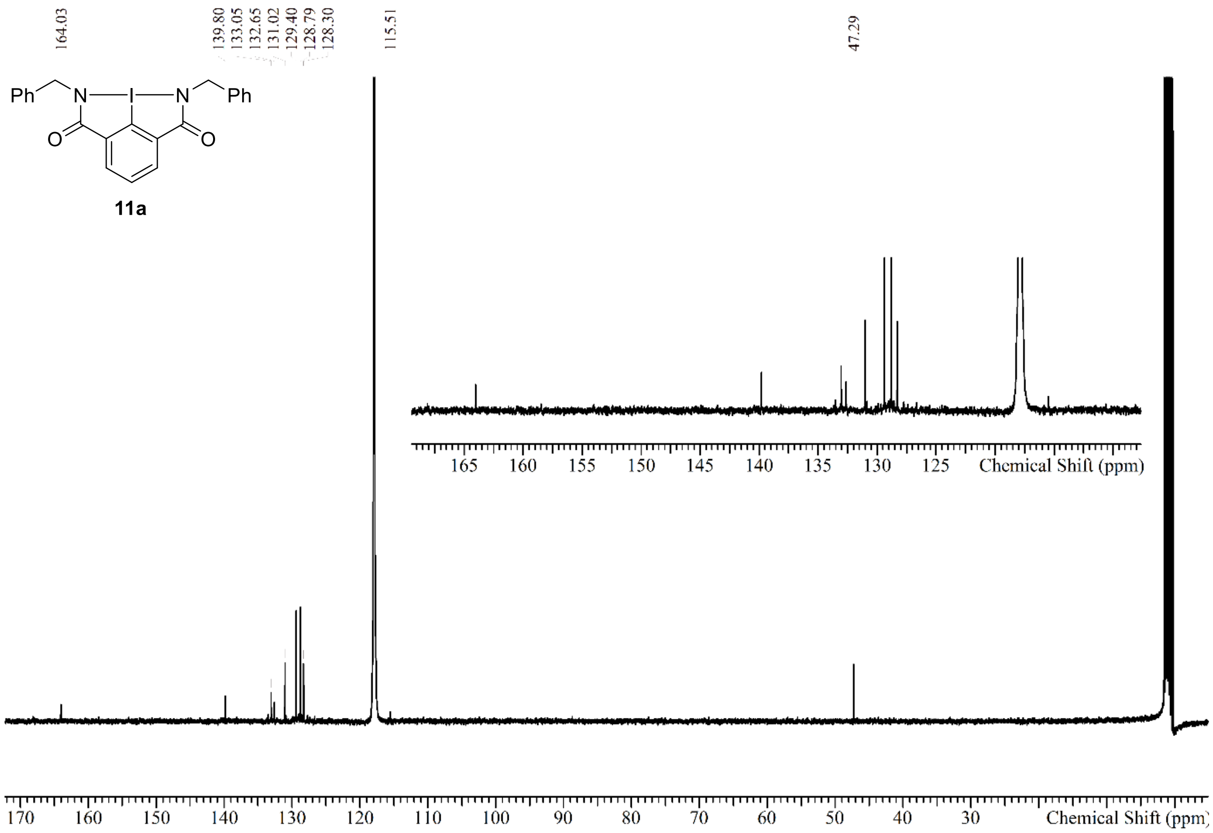

^{1}H NMR spectrum of bis(isopropyl)amidoiodane **11b** – $CDCl_3$

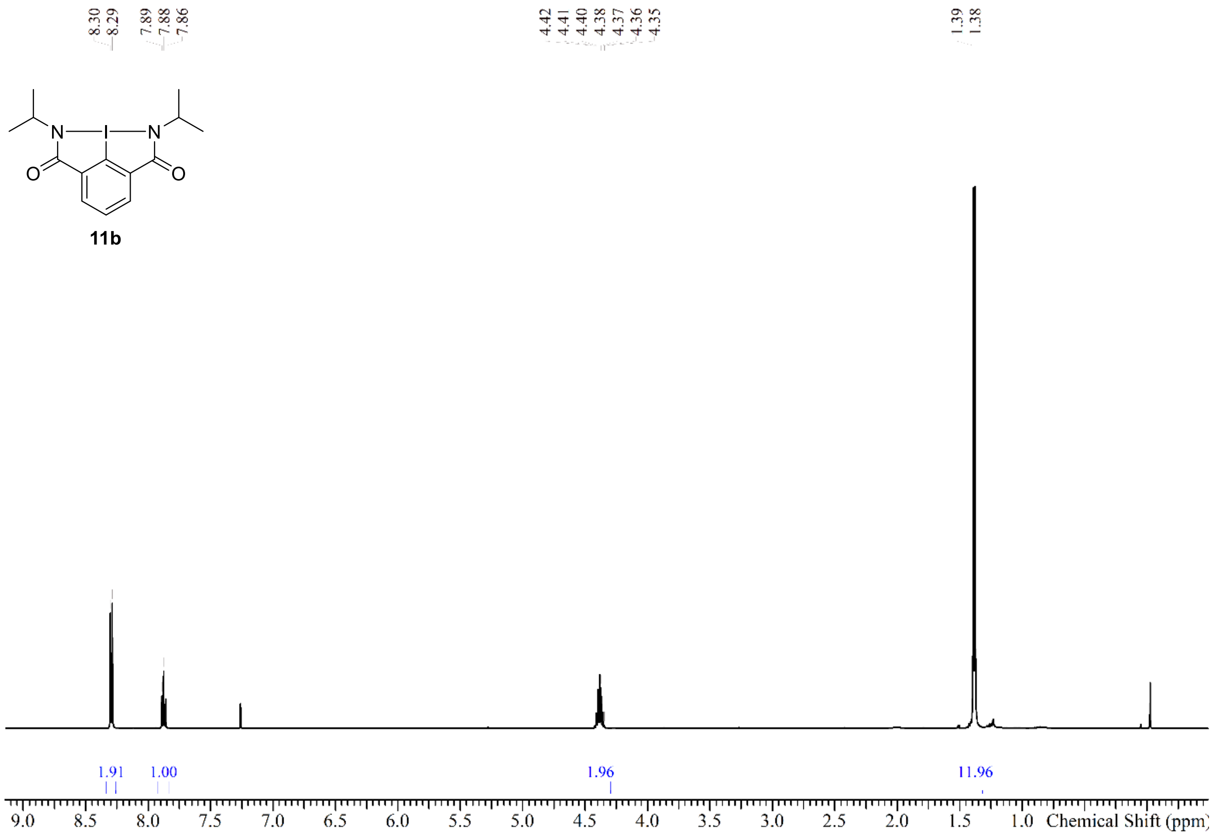

^{13}C NMR spectrum of bis(isopropyl)amidoiodane **11b** – $CDCl_3$

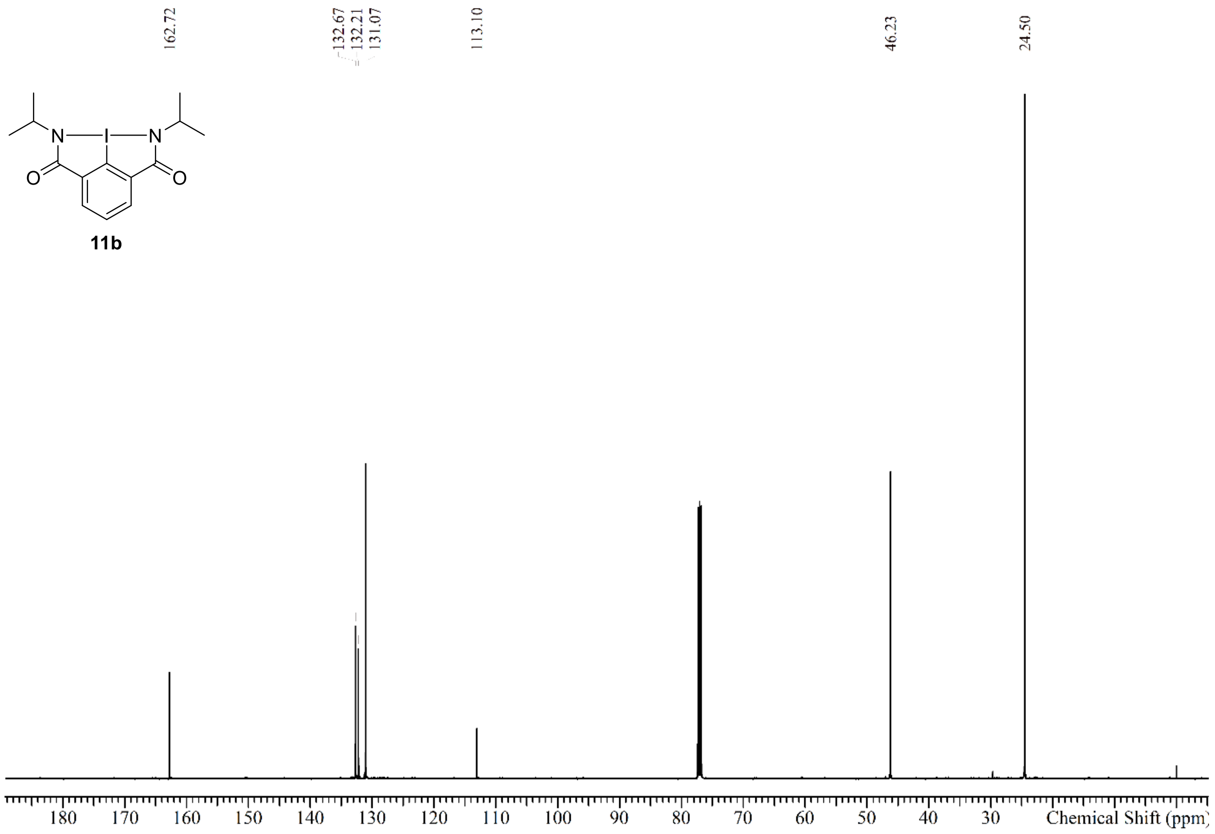

^{1}H NMR spectrum of 2’-iodoacetophenone **13** – $CDCl_3$

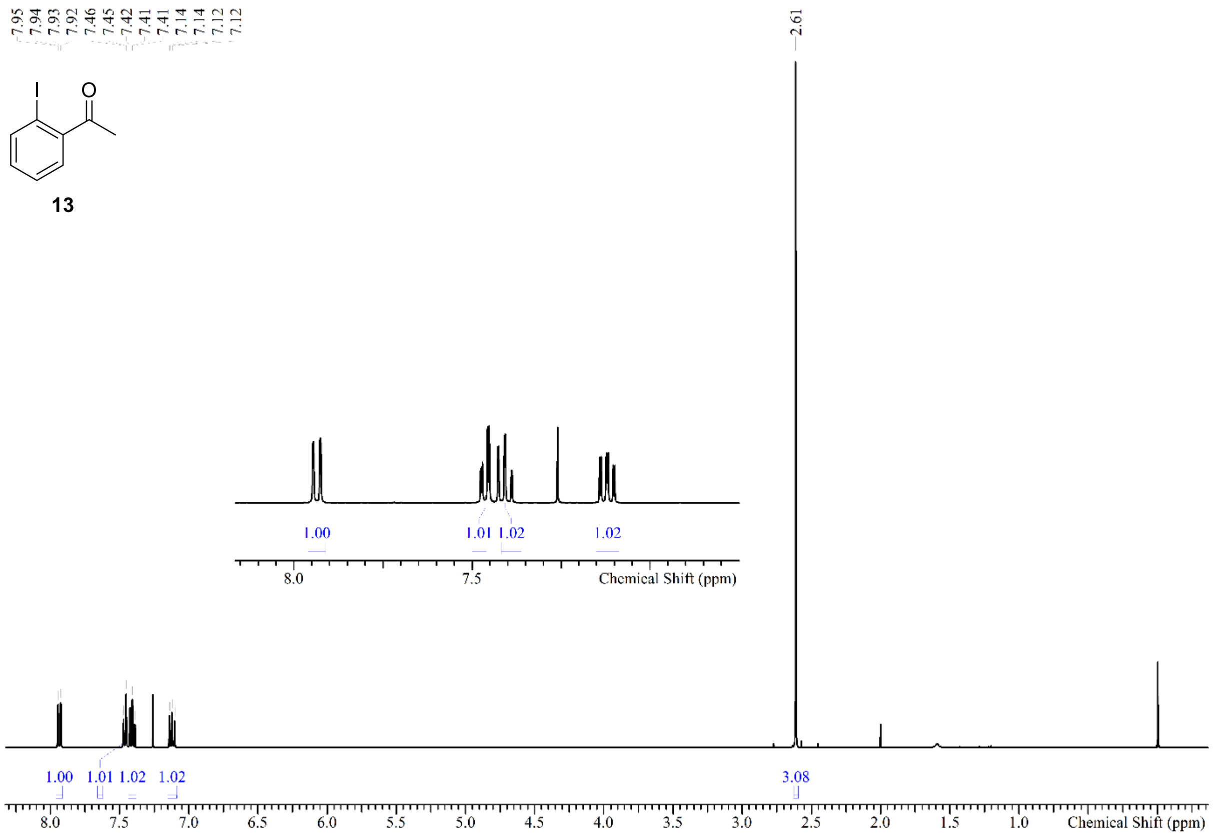

^{13}C NMR spectrum of 2'-iodoacetophenone **13** – $CDCl_3$

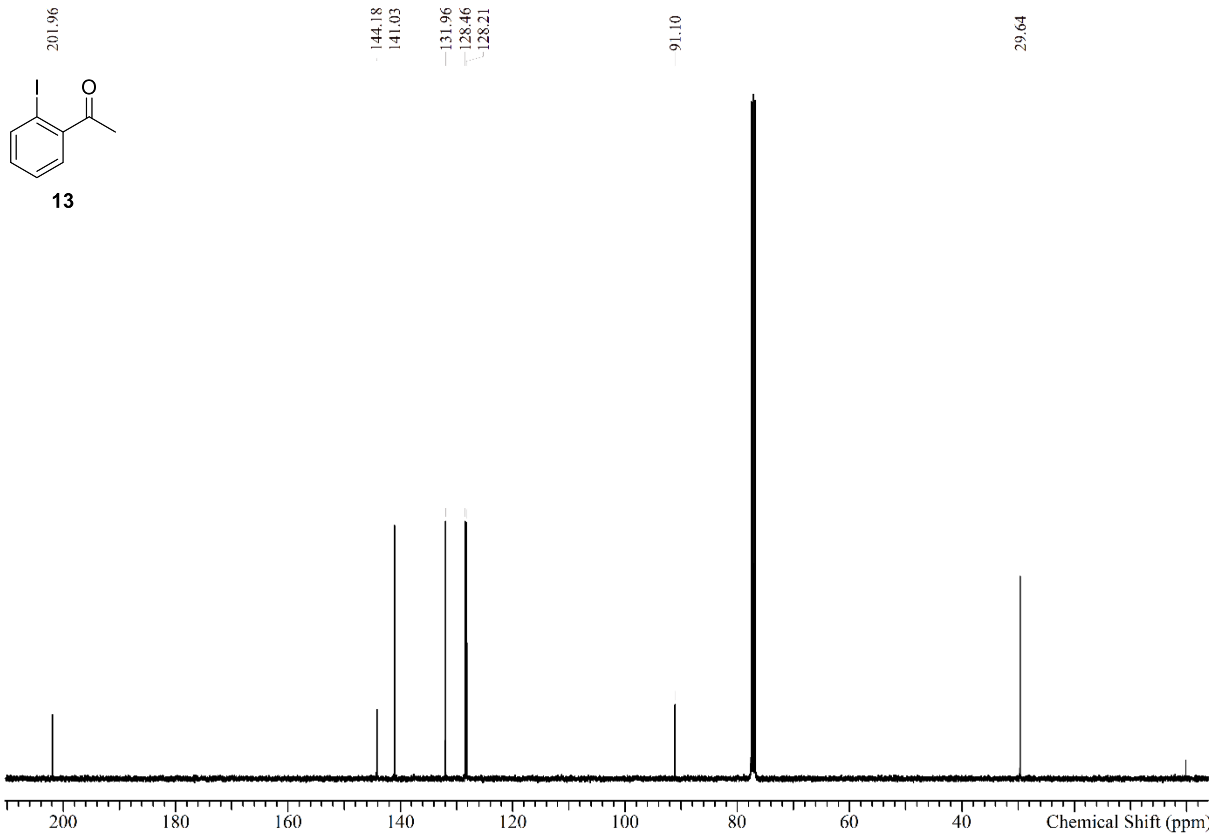

^{1}H NMR spectrum of methyl(trifluoromethyl)iodoalcohol **14** – $CDCl_3$

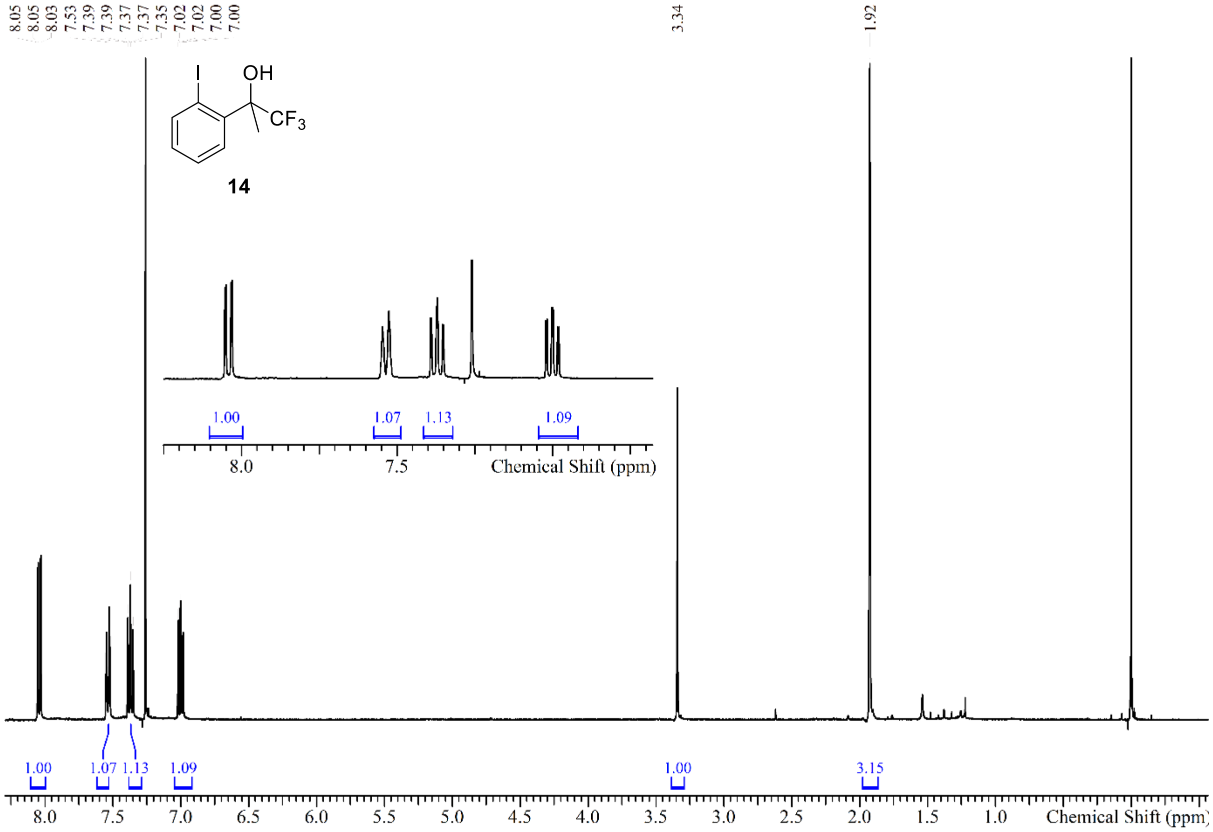

^{13}C NMR spectrum of methyl(trifluoromethyl)iodoalcohol **14** – $CDCl_3$

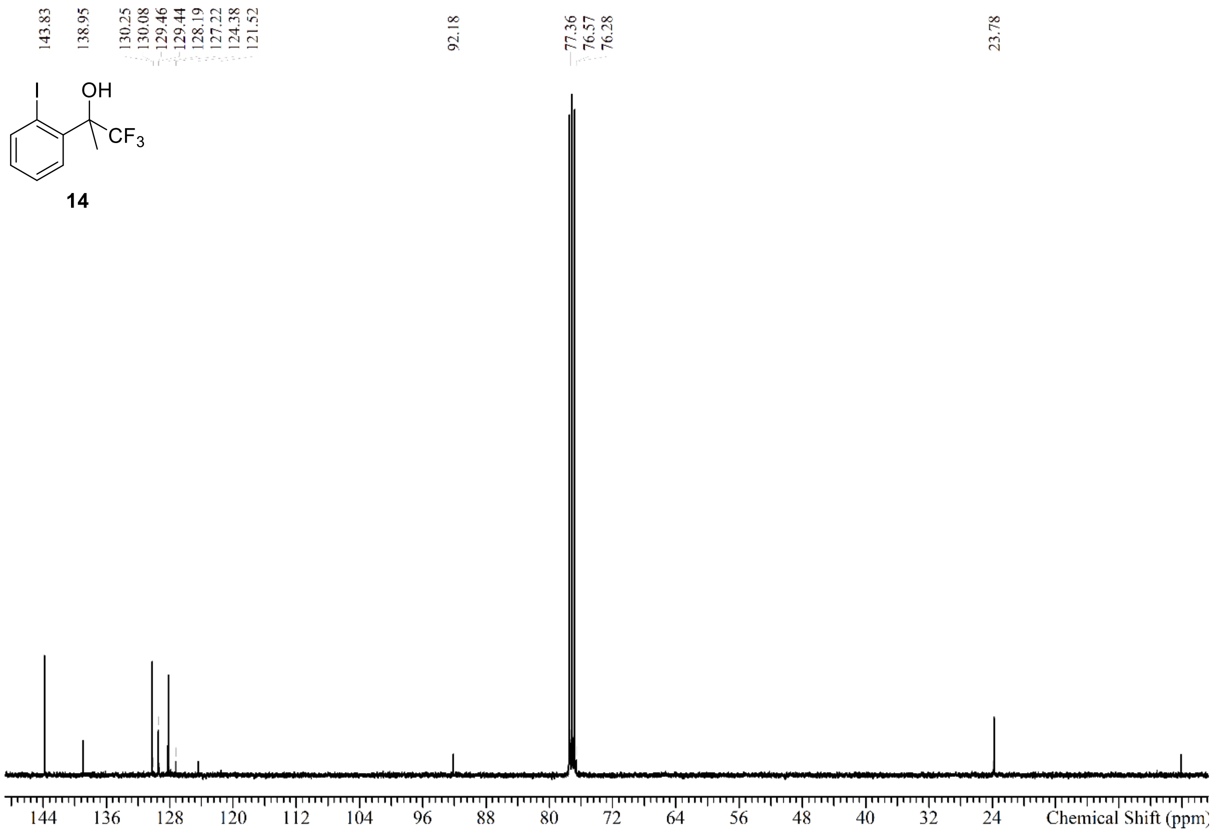

^{19}F NMR spectrum of methyl(trifluoromethyl)iodoalcohol **14** – $CDCl_3$

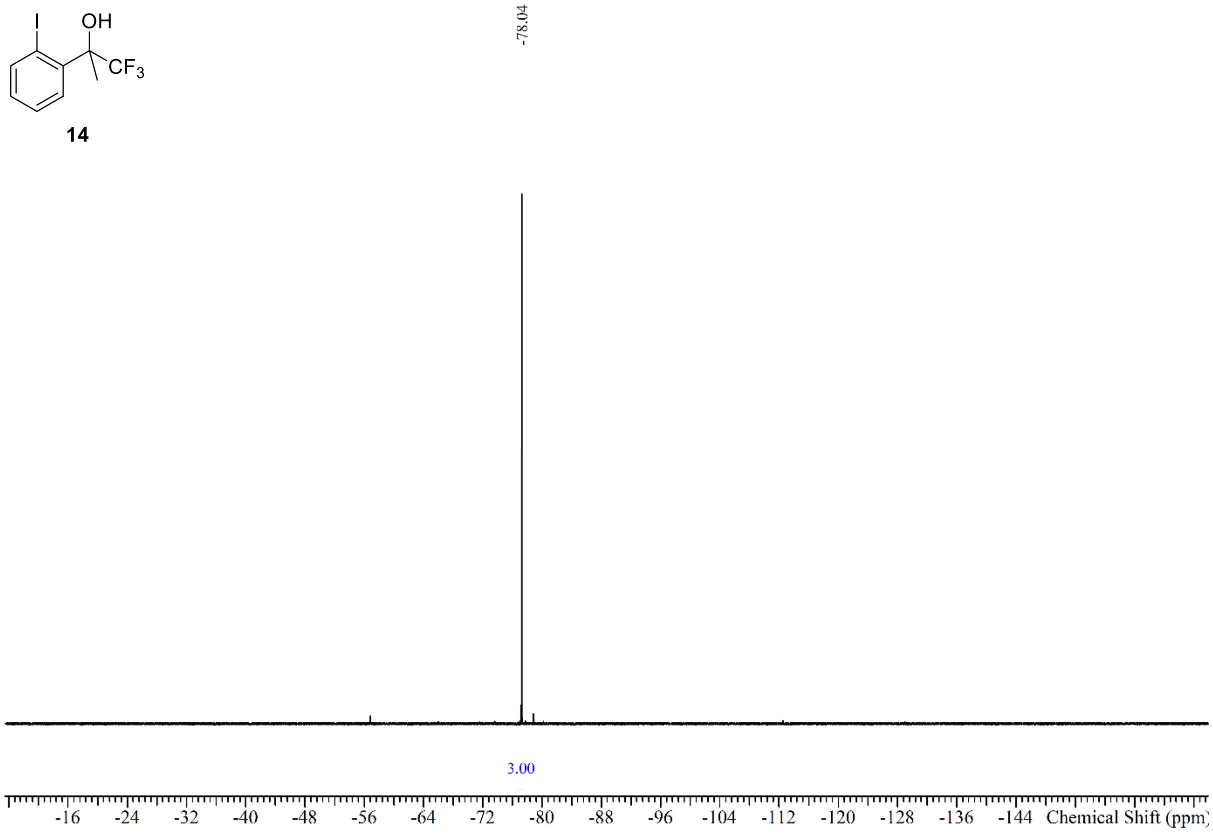

^{1}H NMR spectrum of methyl(trifluoromethyl)fluoroiodane **15** – $CDCl_3$

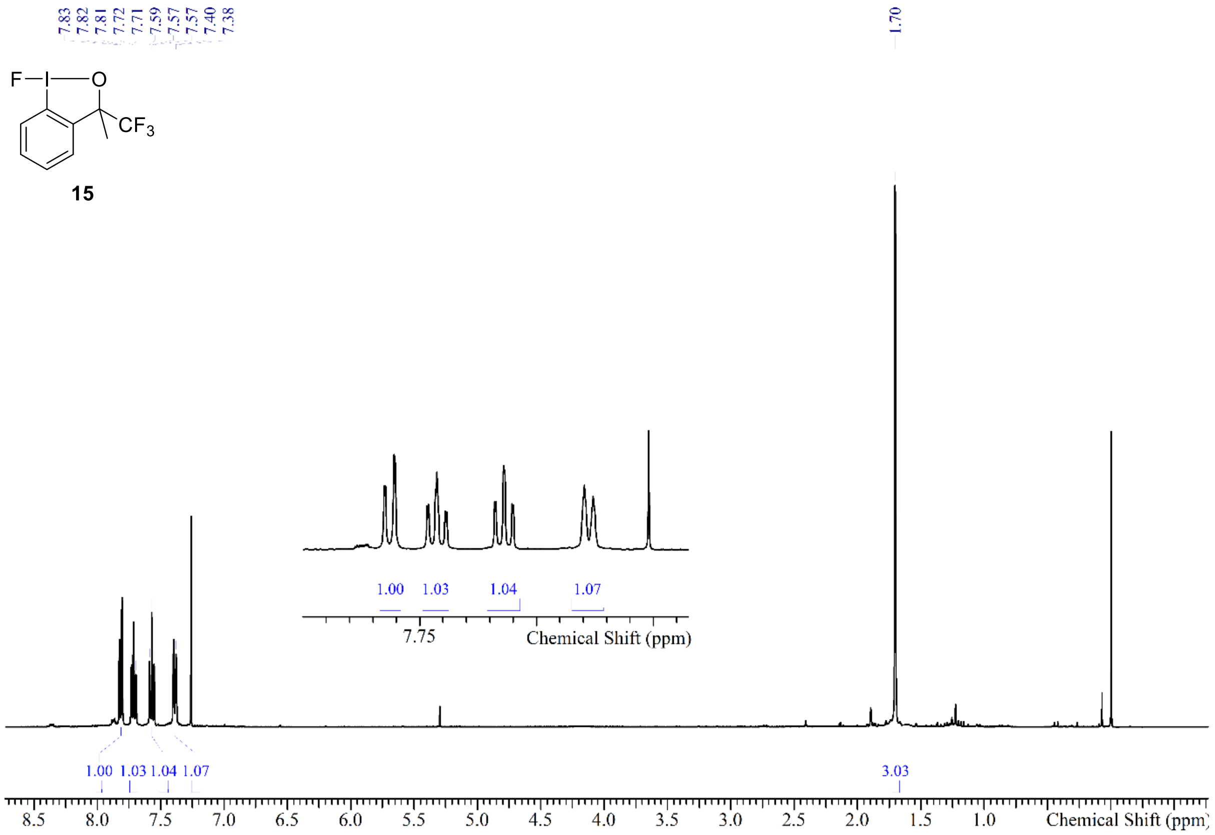

^{13}C NMR spectrum of methyl(trifluoromethyl)fluoroiodane **15** – $CDCl_3$

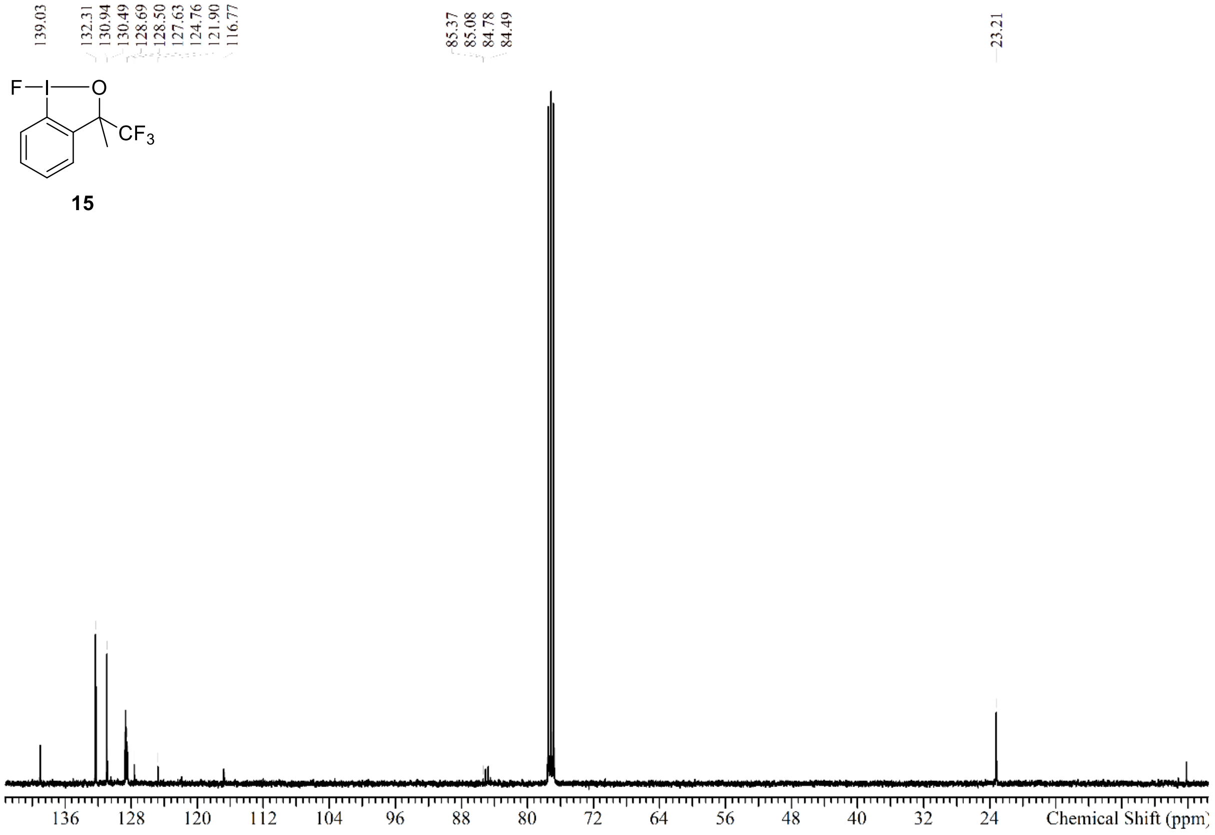

^{19}F NMR spectrum of methyl(trifluoromethyl)fluoroiodane **15** – $CDCl_3$

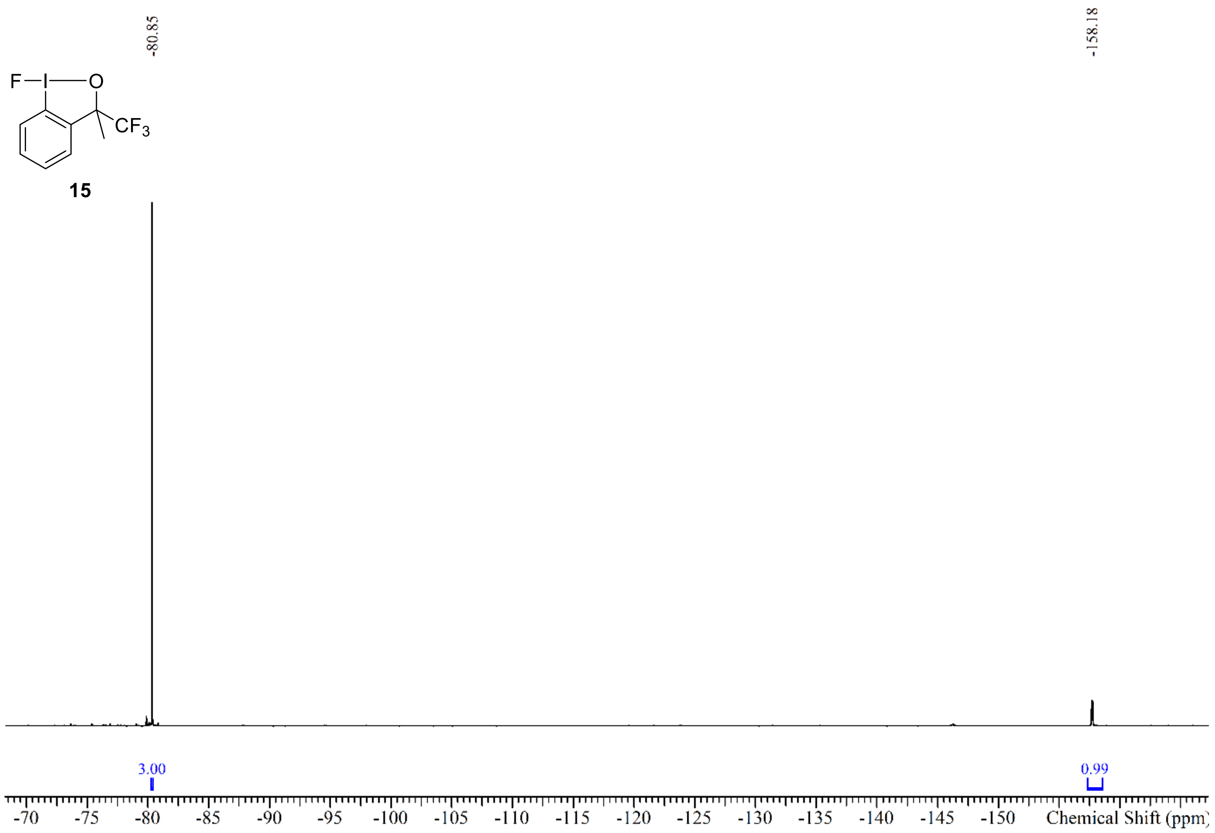

^{1}H NMR spectrum of methyl(trifluoromethyl)iodosyl fluoride **S10** – CD_3CN

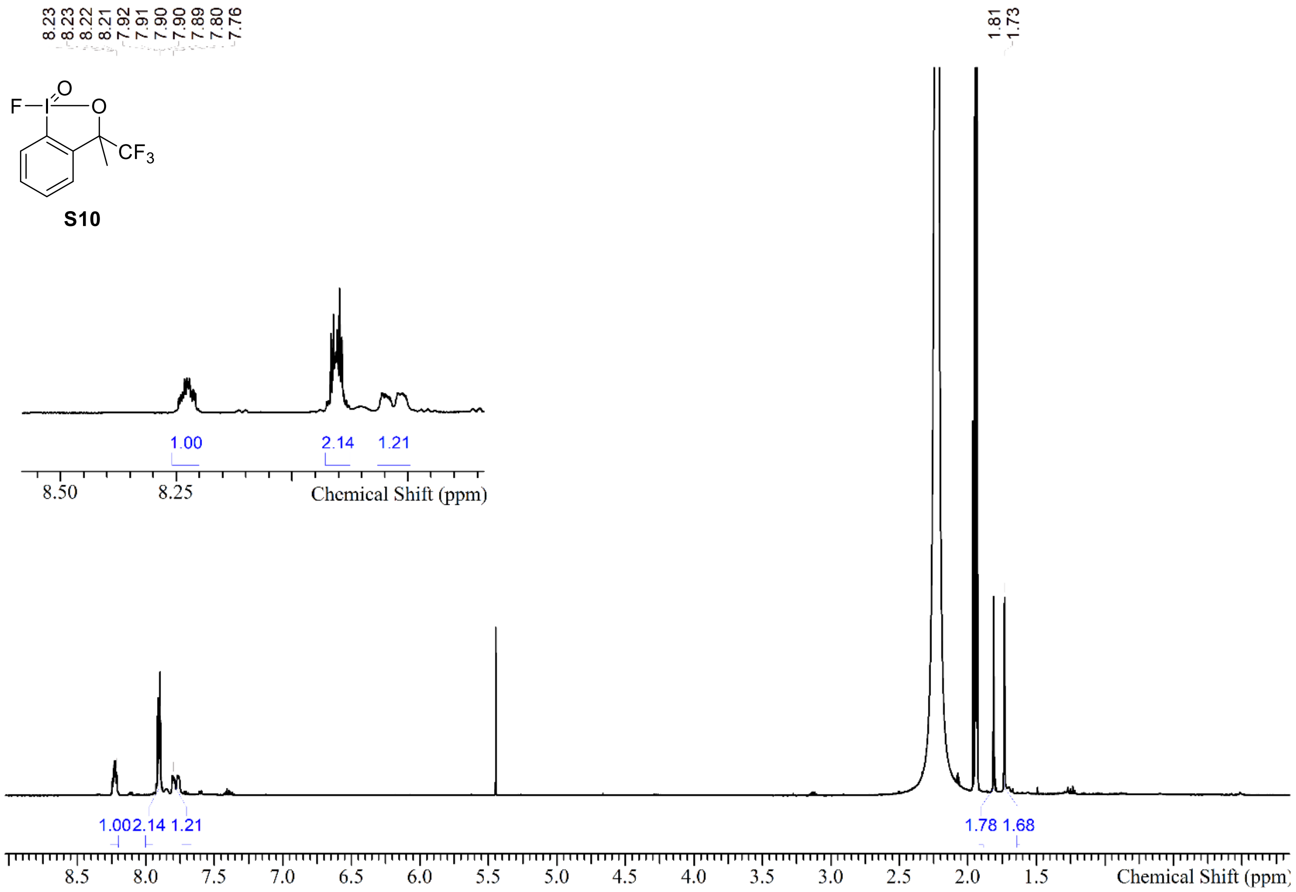

^{19}F NMR spectrum of methyl(trifluoromethyl)iodosyl fluoride **S10** – CD_3CN

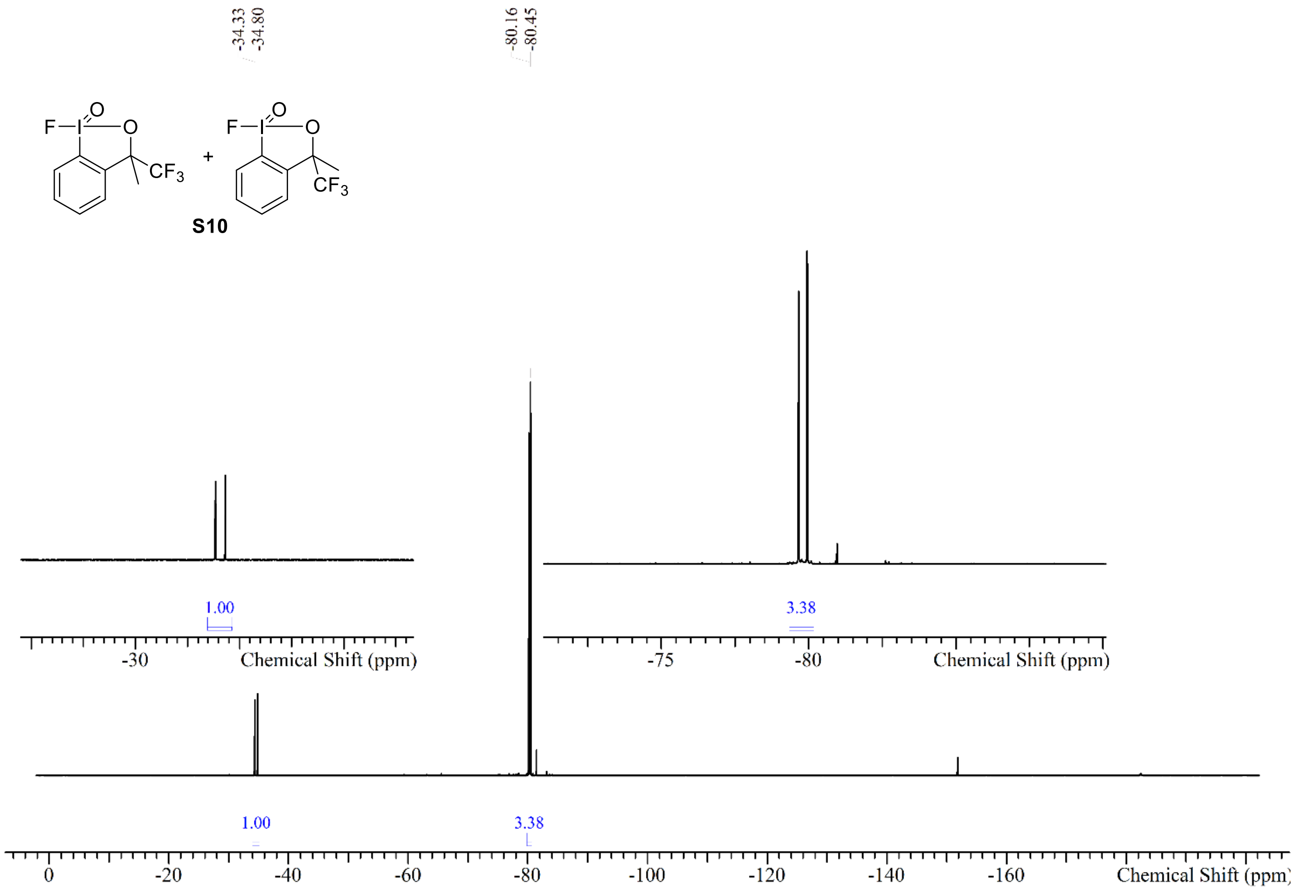

^{1}H NMR spectrum of trifluoromethylketone **17** – $CDCl_3$

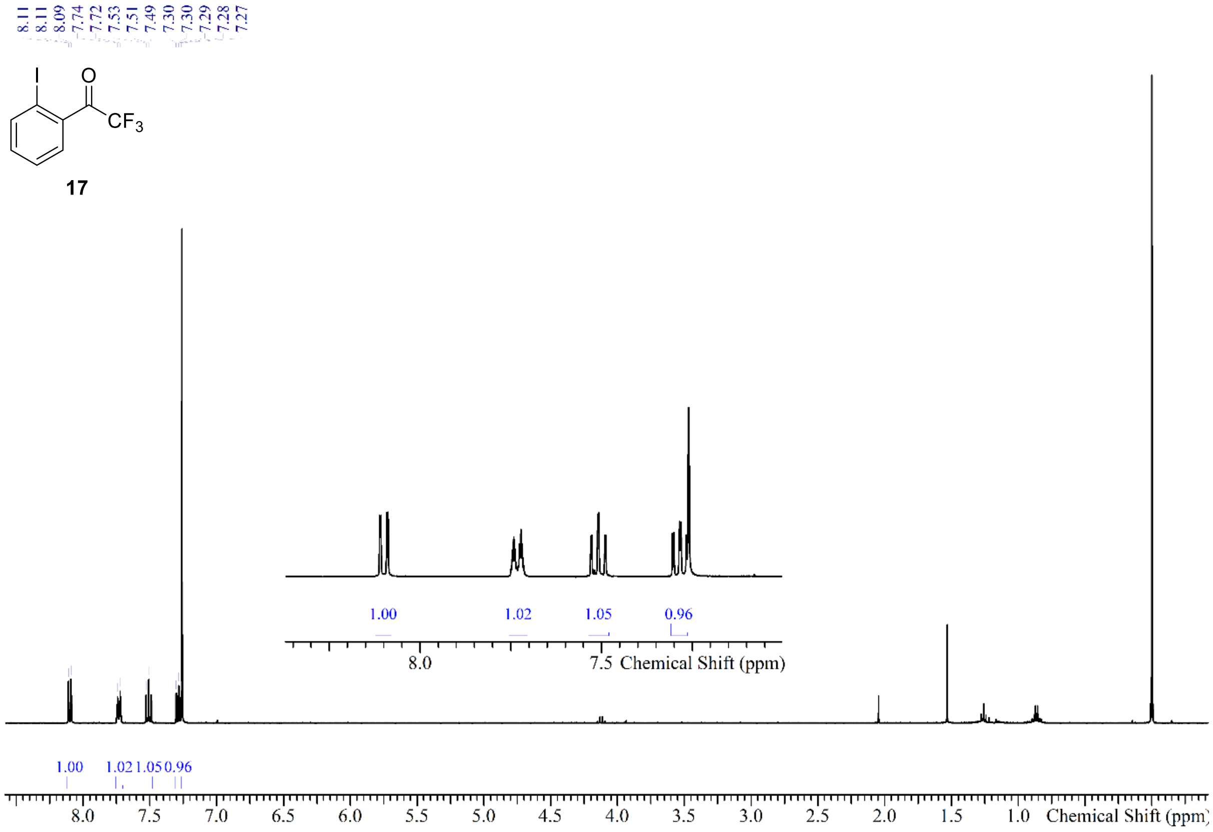

^{13}C NMR spectrum of trifluoromethylketone **17** – $CDCl_3$

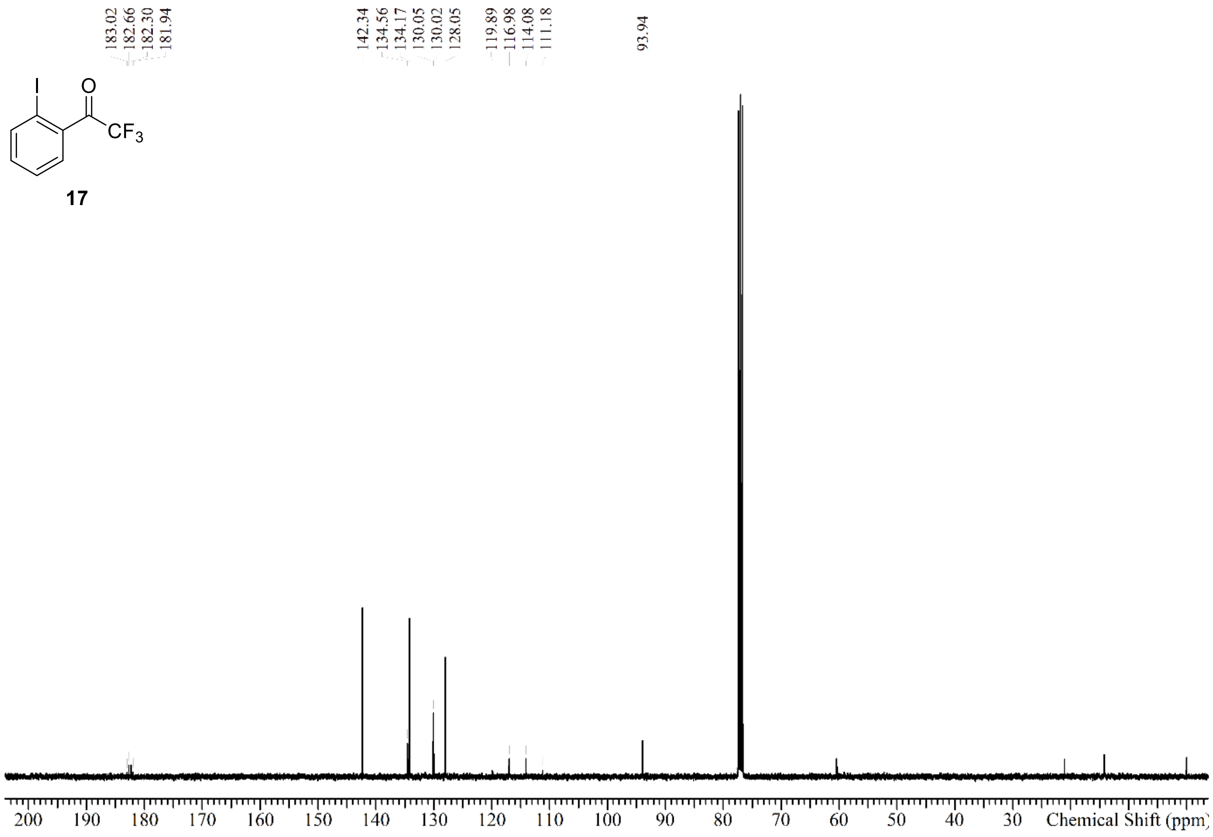

^{19}F NMR spectrum of trifluoromethylketone **17** – $CDCl_3$

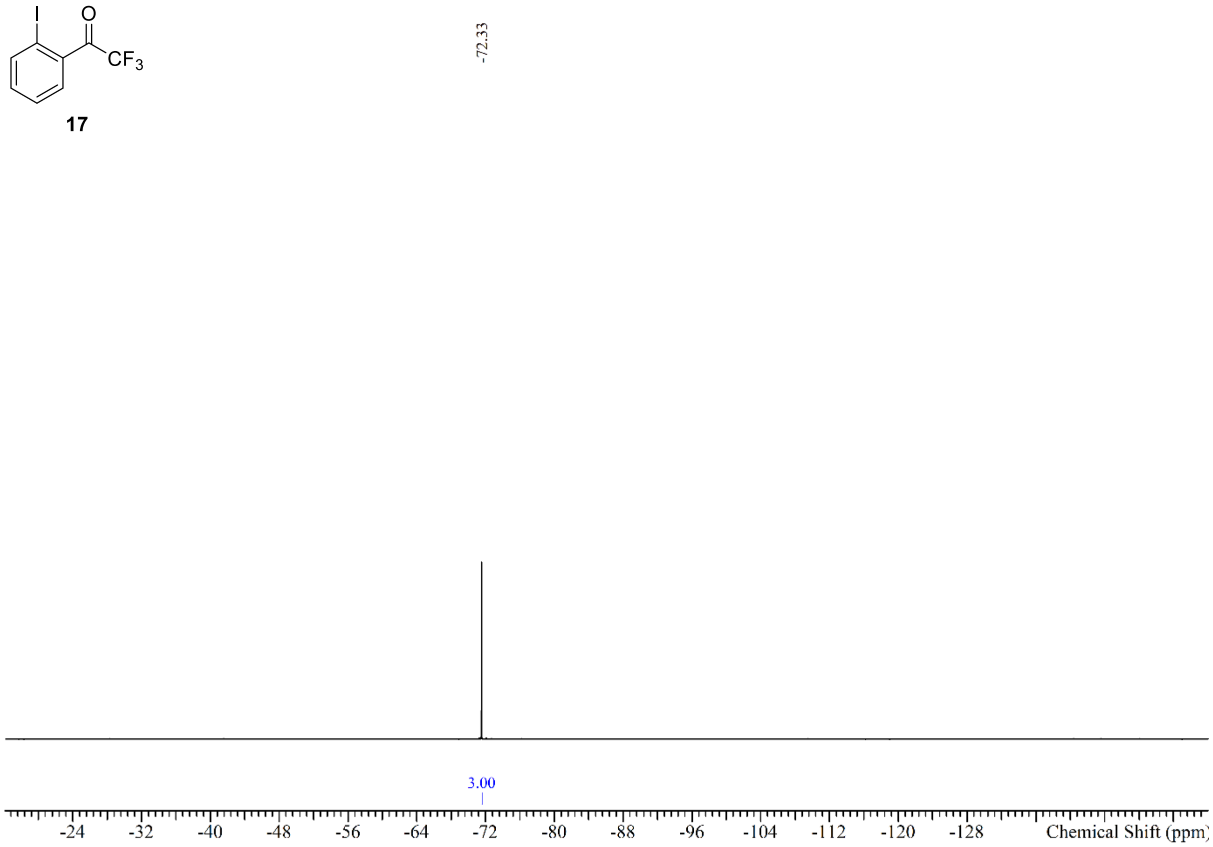

^{1}H NMR spectrum of bis(trifluoromethyl)iodoalcohol **18** – $CDCl_3$

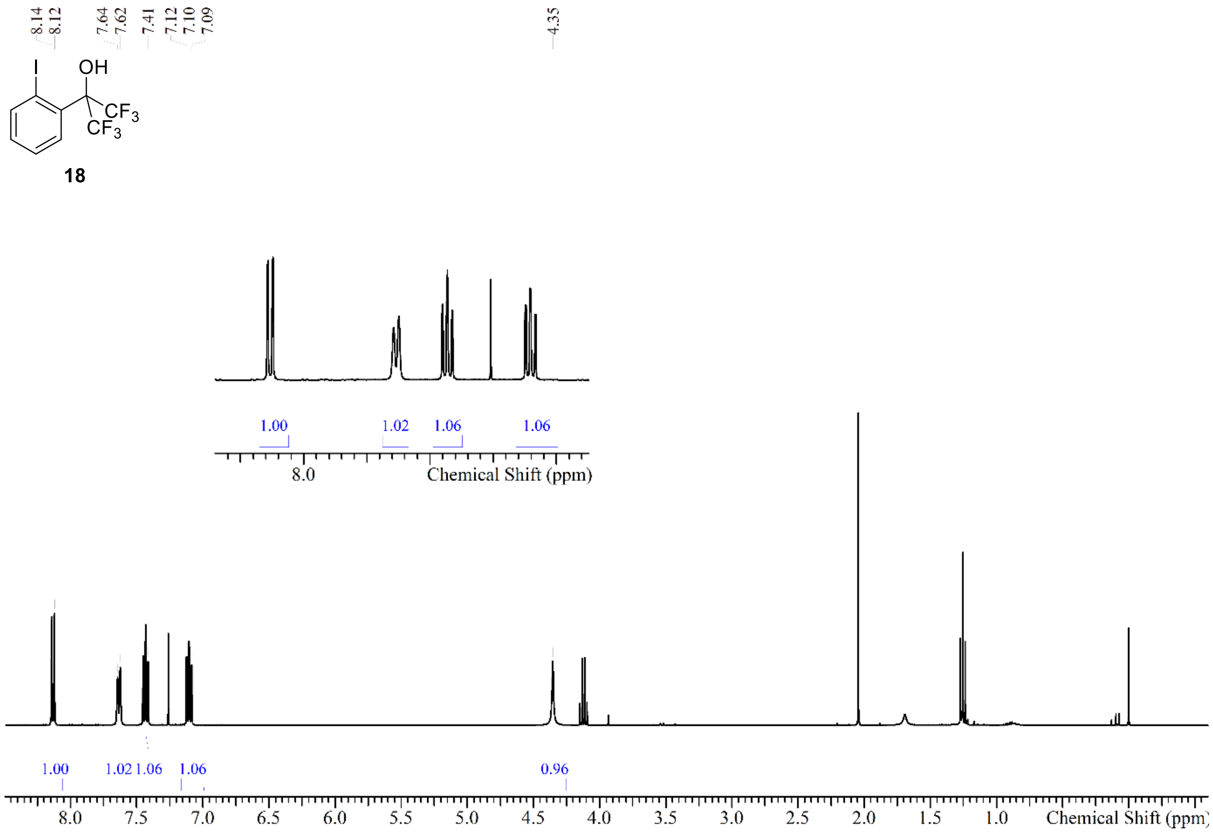

^{13}C NMR spectrum of bis(trifluoromethyl)iodoalcohol **18** – $CDCl_3$

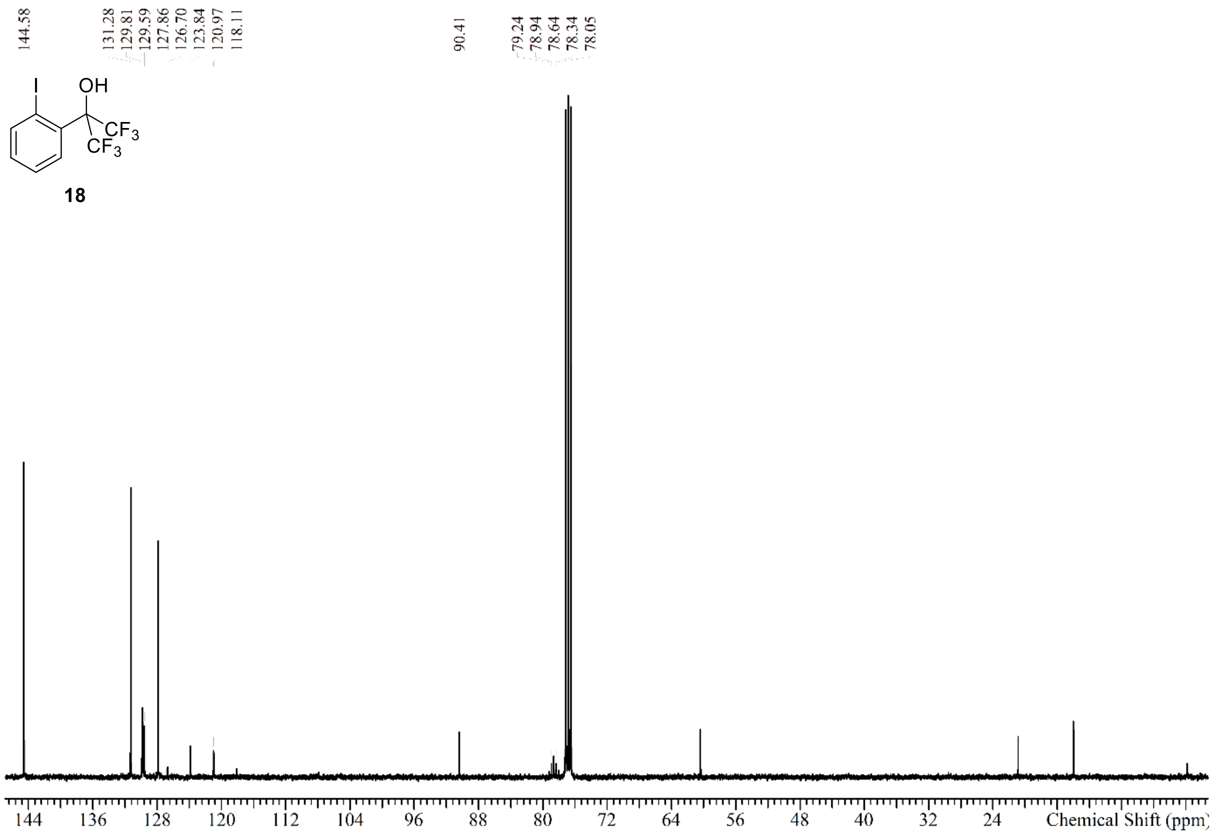

^{19}F NMR spectrum of bis(trifluoromethyl)iodoalcohol **18** – $CDCl_3$

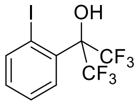

18

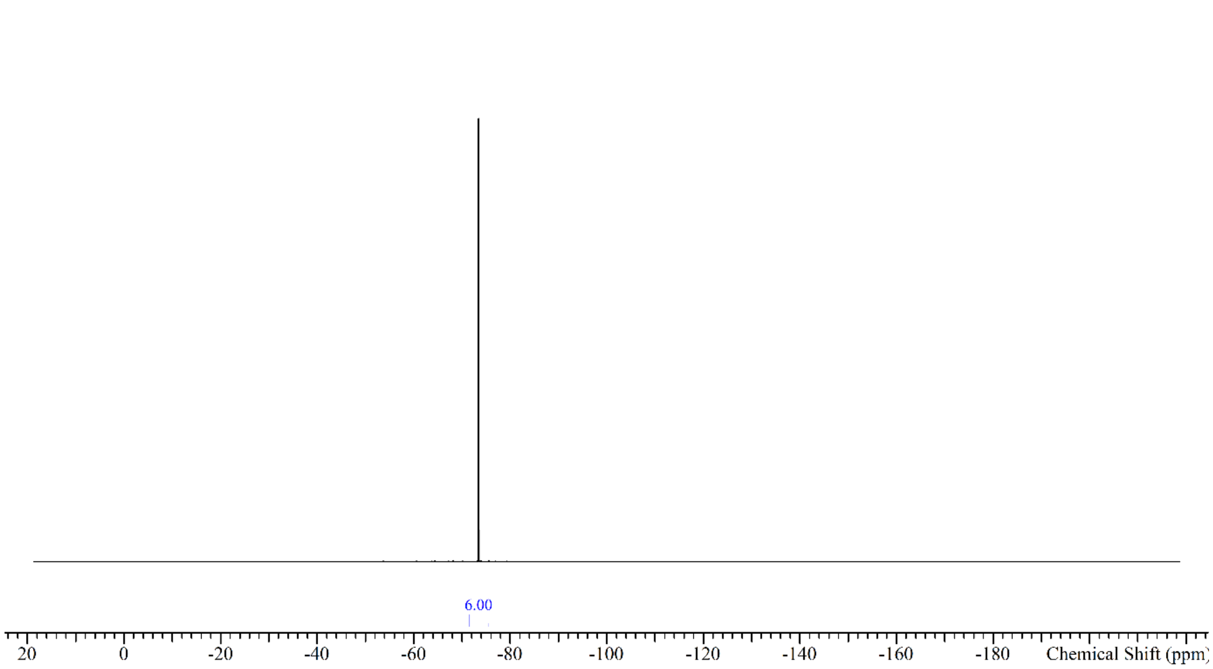

^{1}H NMR spectrum of bis(trifluoromethyl)fluoroiodane **19** – CD_3CN

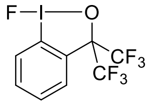

19

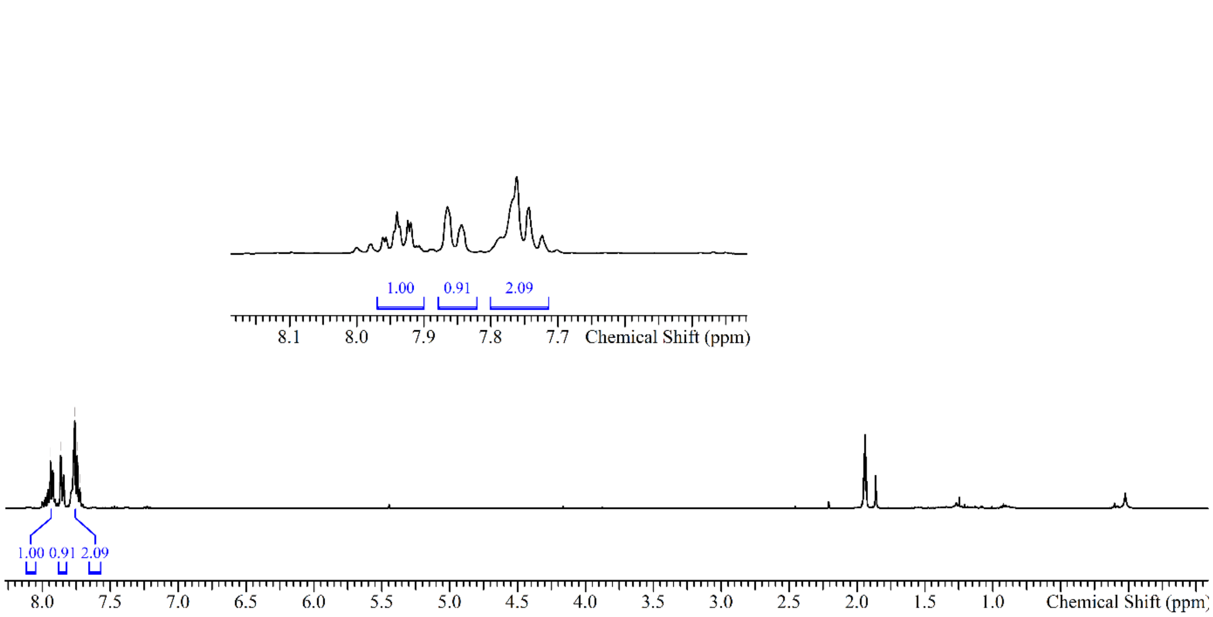

^{13}C NMR spectrum of bis(trifluoromethyl)fluoroiodane **19** – CD_3CN

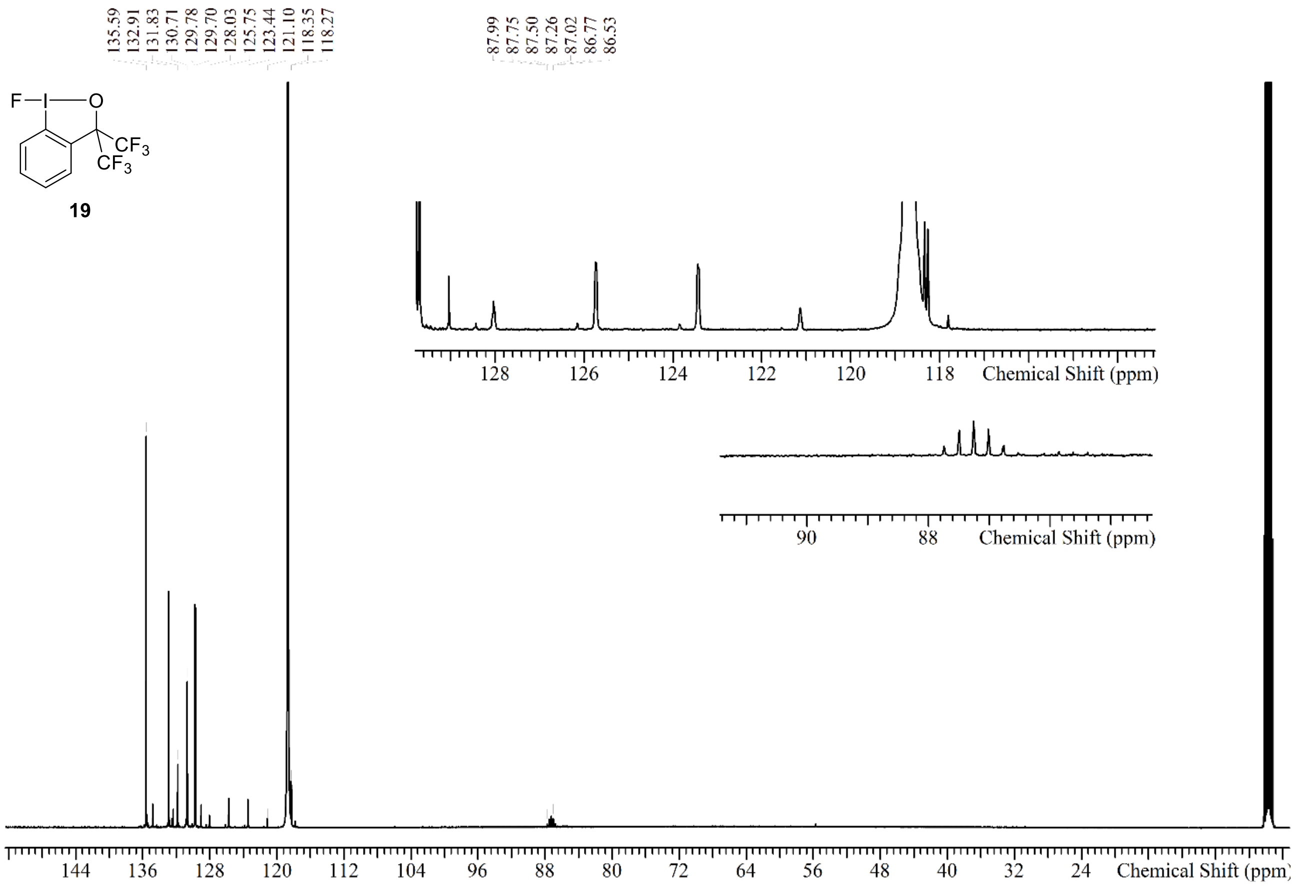

^{19}F NMR spectrum of bis(trifluoromethyl)fluoroiodane **19** – CD_3CN

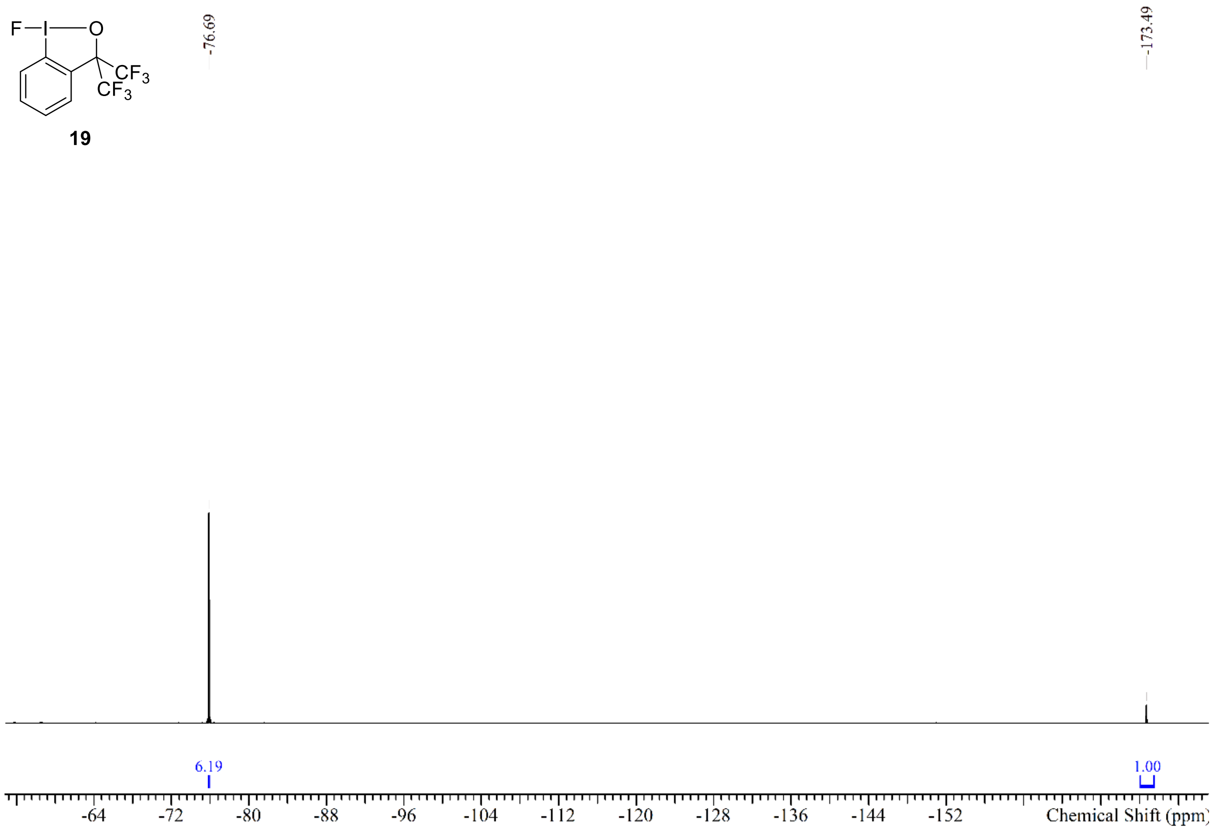

^{1}H NMR spectrum of dimethyltrifluoroiodane **20** – CD_3CN

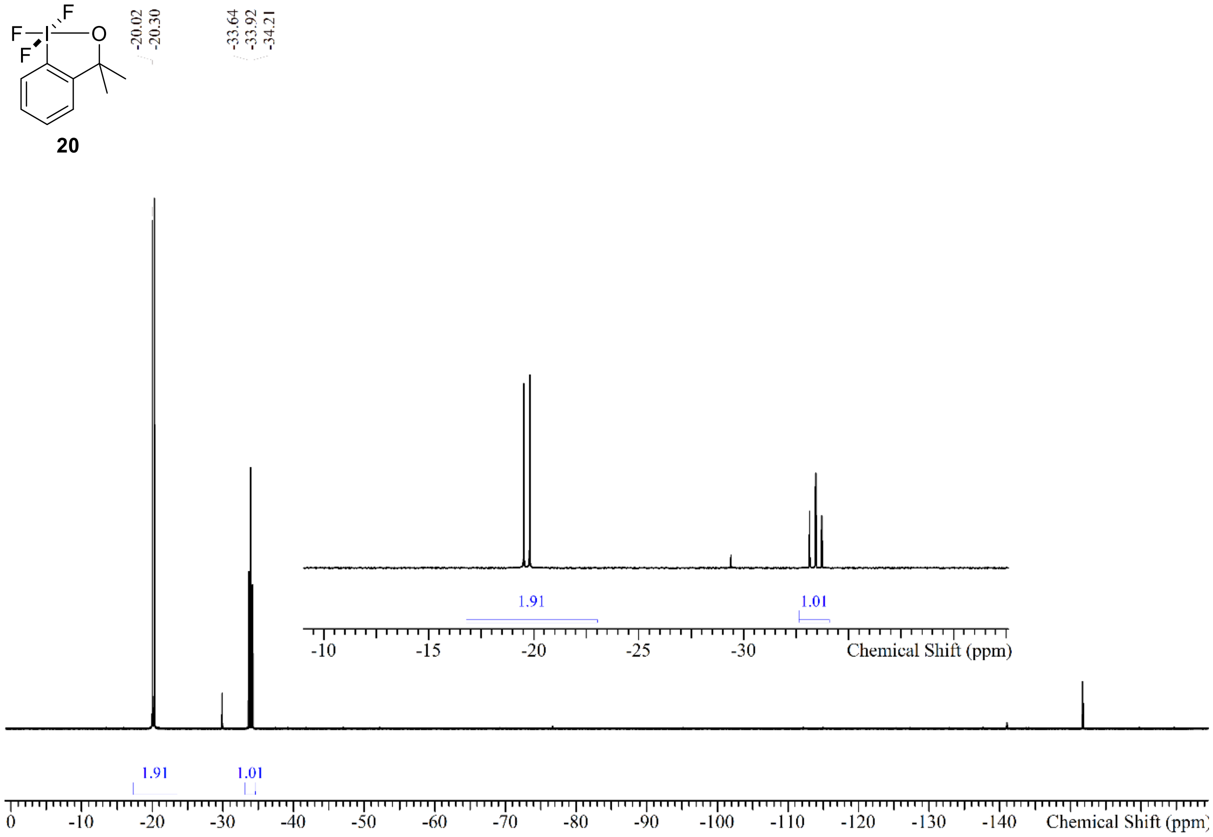

^{13}C NMR spectrum of dimethyltrifluoroiodane **20** – CD_3CN

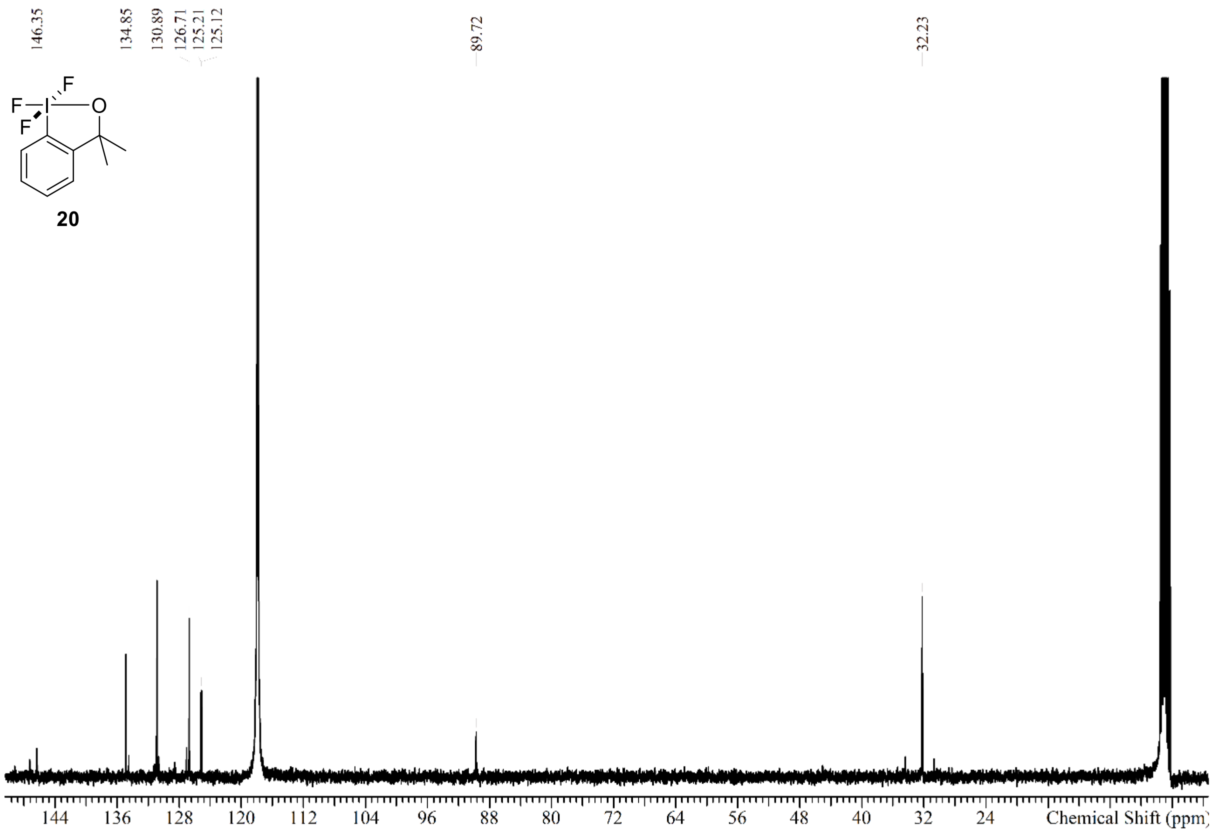

^{19}F NMR spectrum of dimethyltrifluoroiodane **20** – CD_3CN

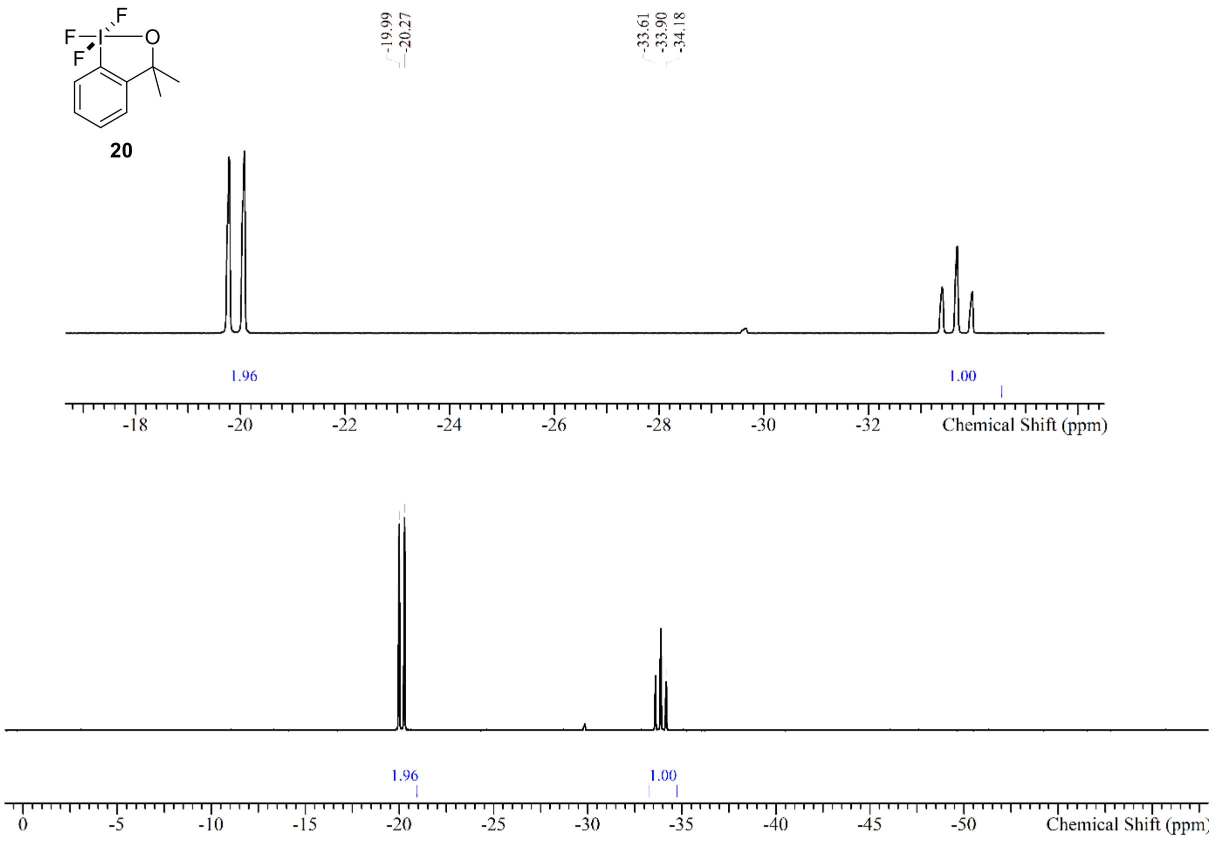

^{1}H NMR spectrum of dimethyliodosyl fluoride **S13** – CD_3CN

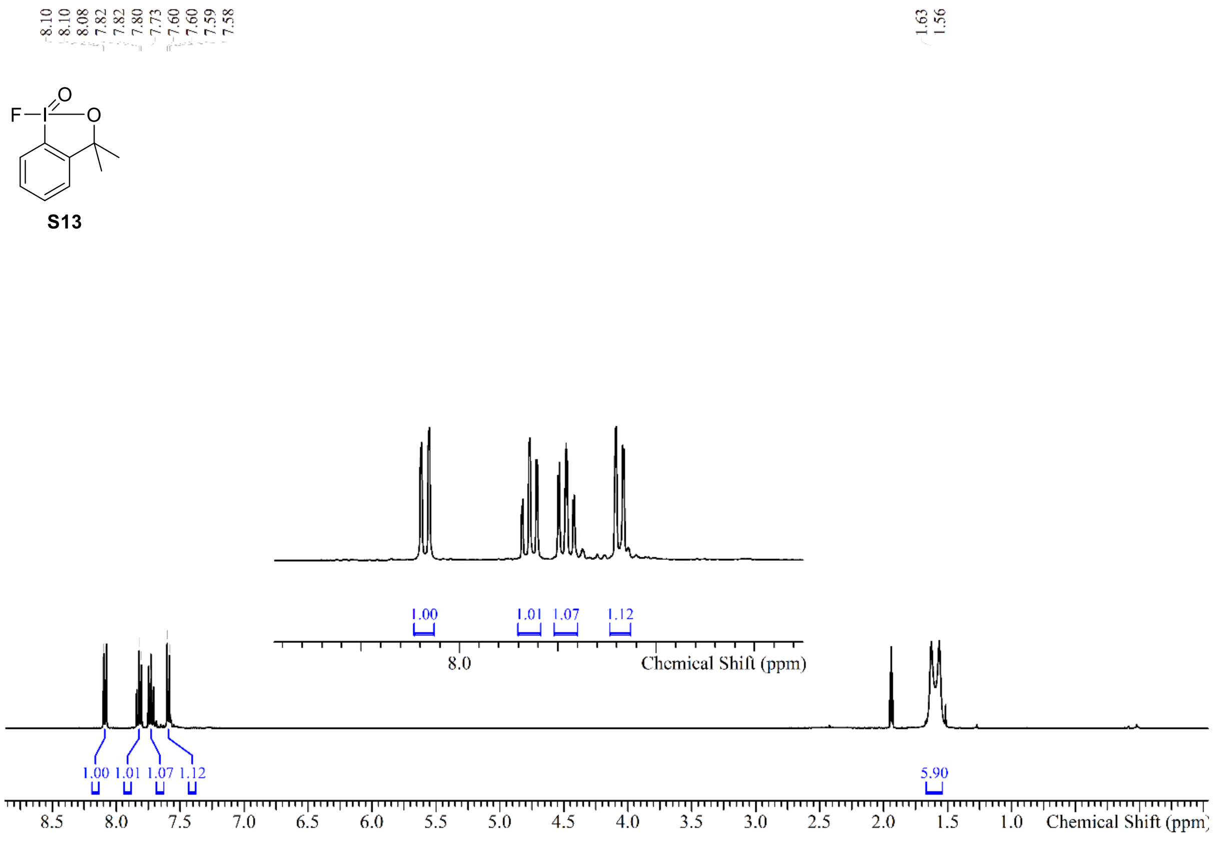

^{13}C NMR spectrum of dimethyliodosyl fluoride **S13** – CD_3CN

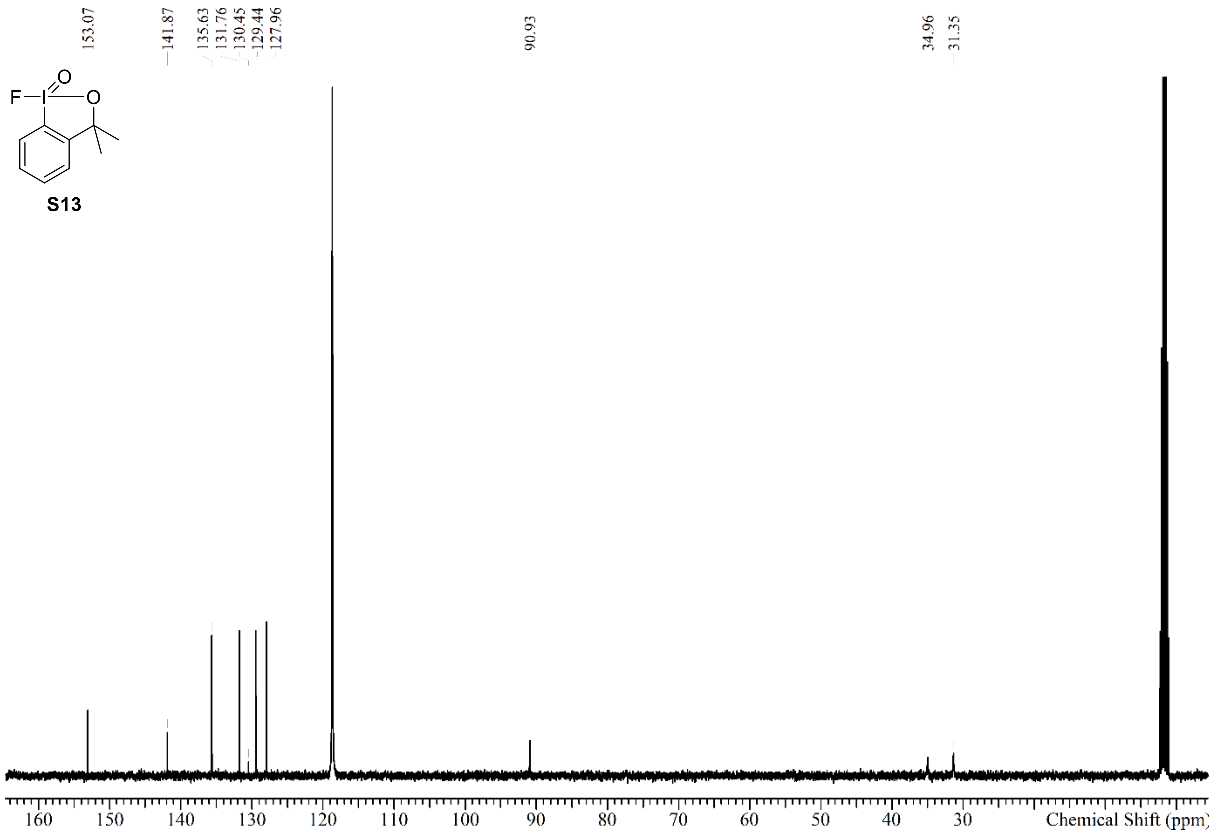

^{19}F NMR spectrum of dimethyliodosyl fluoride **S13** – CD_3CN

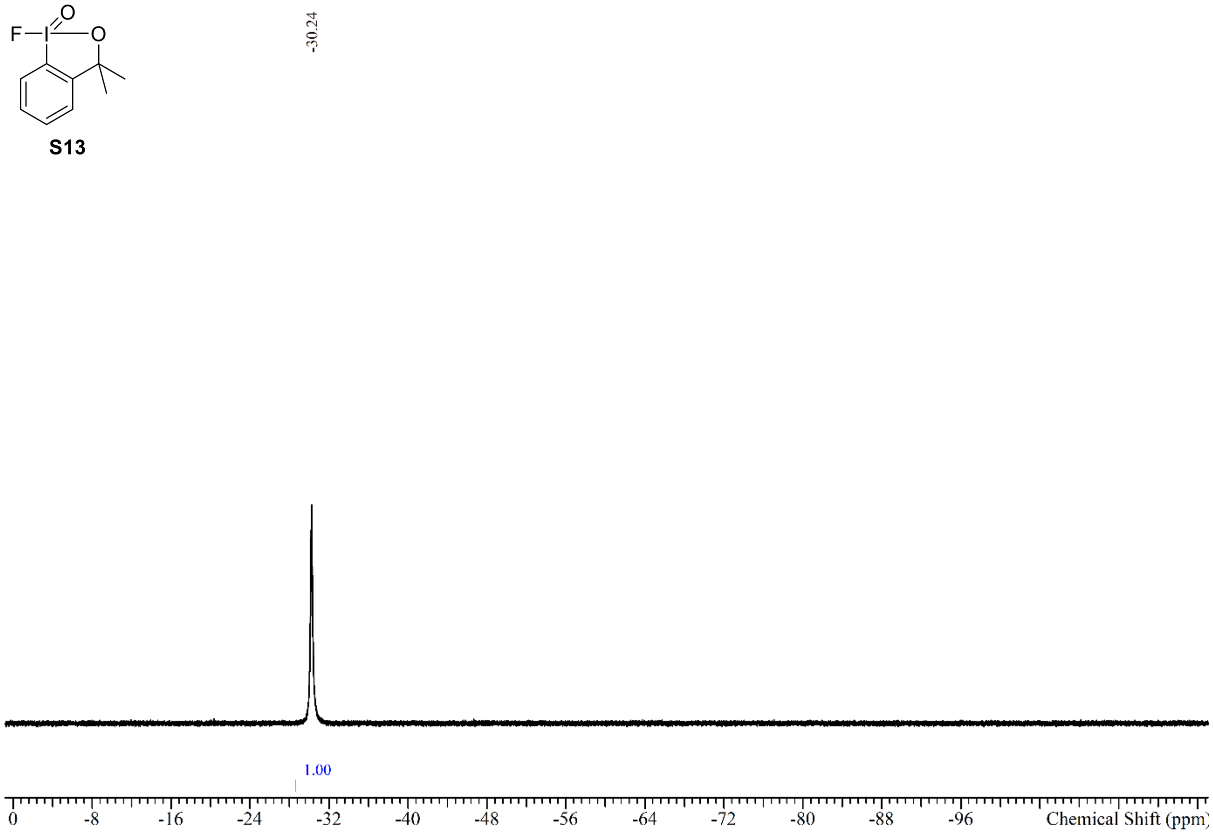

^{1}H NMR spectrum of methyl(trifluoromethyl)trifluoroiodane **21** – CD_3CN

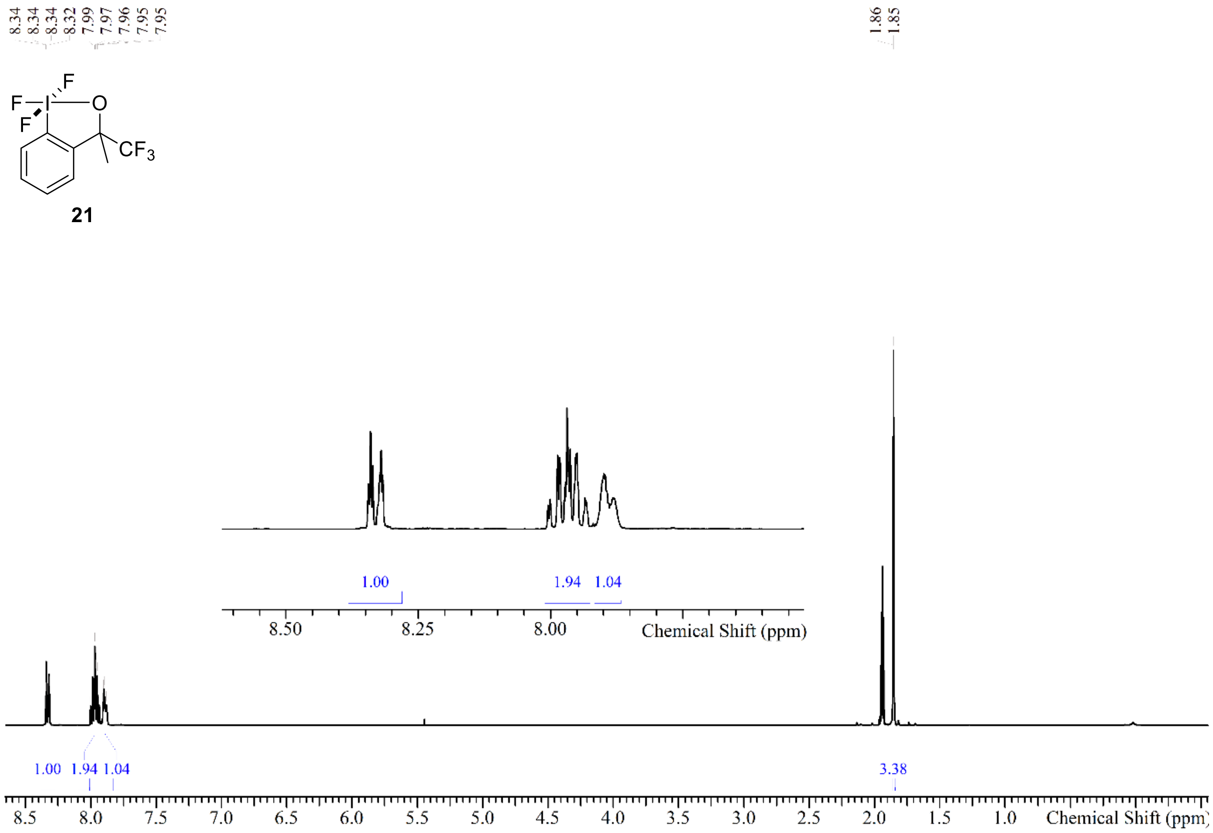

^{13}C NMR spectrum of methyl(trifluoromethyl)trifluoroiodane **21** – CD_3CN

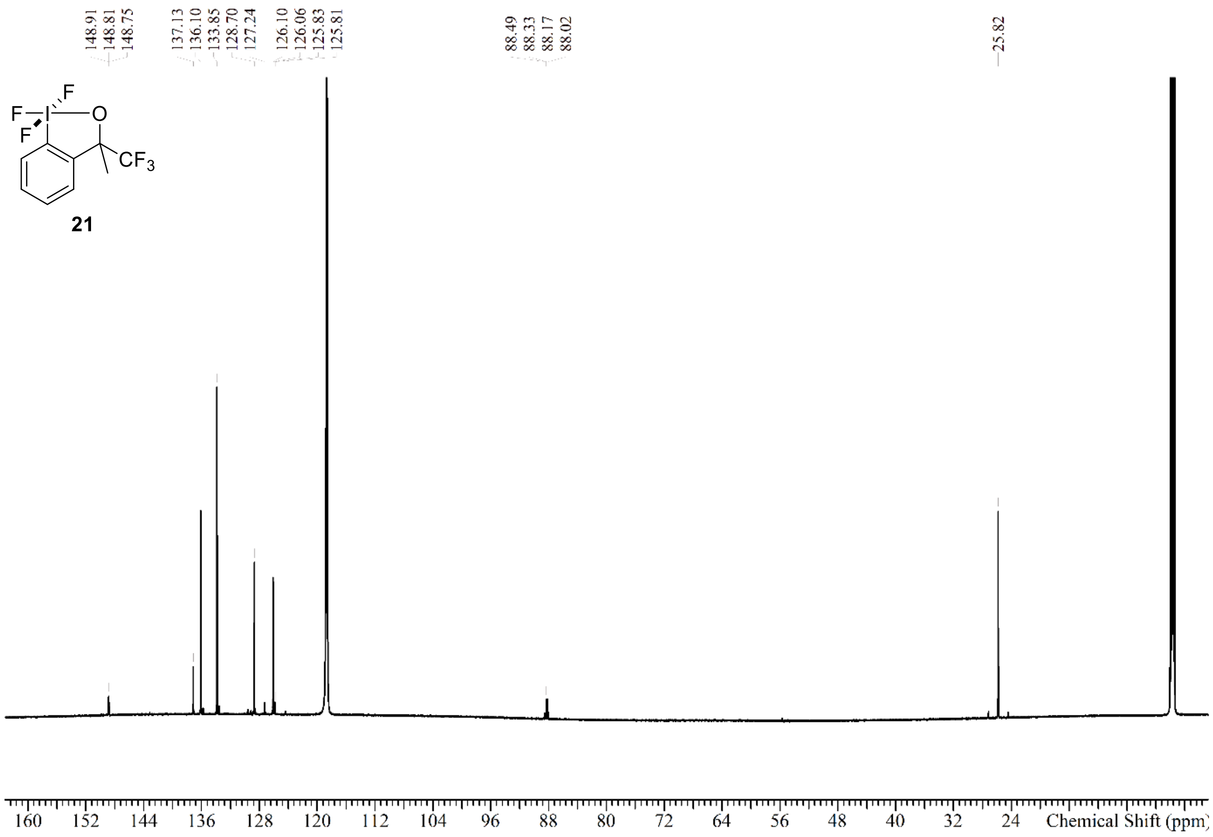

^{19}F NMR spectrum of methyl(trifluoromethyl)trifluoroiodane **21** – CD_3CN

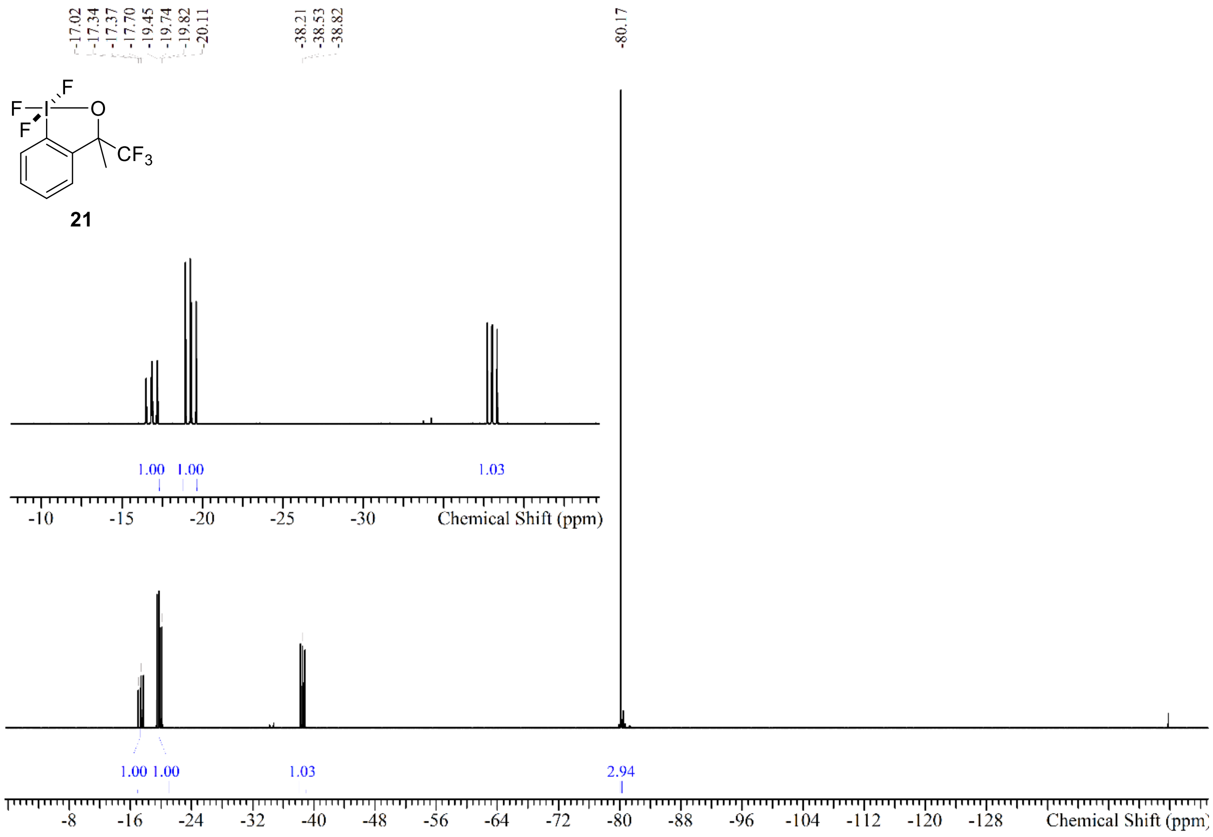

^{1}H NMR spectrum of bis(trifluoromethyl)trifluoroiodane **22** – CD_3CN

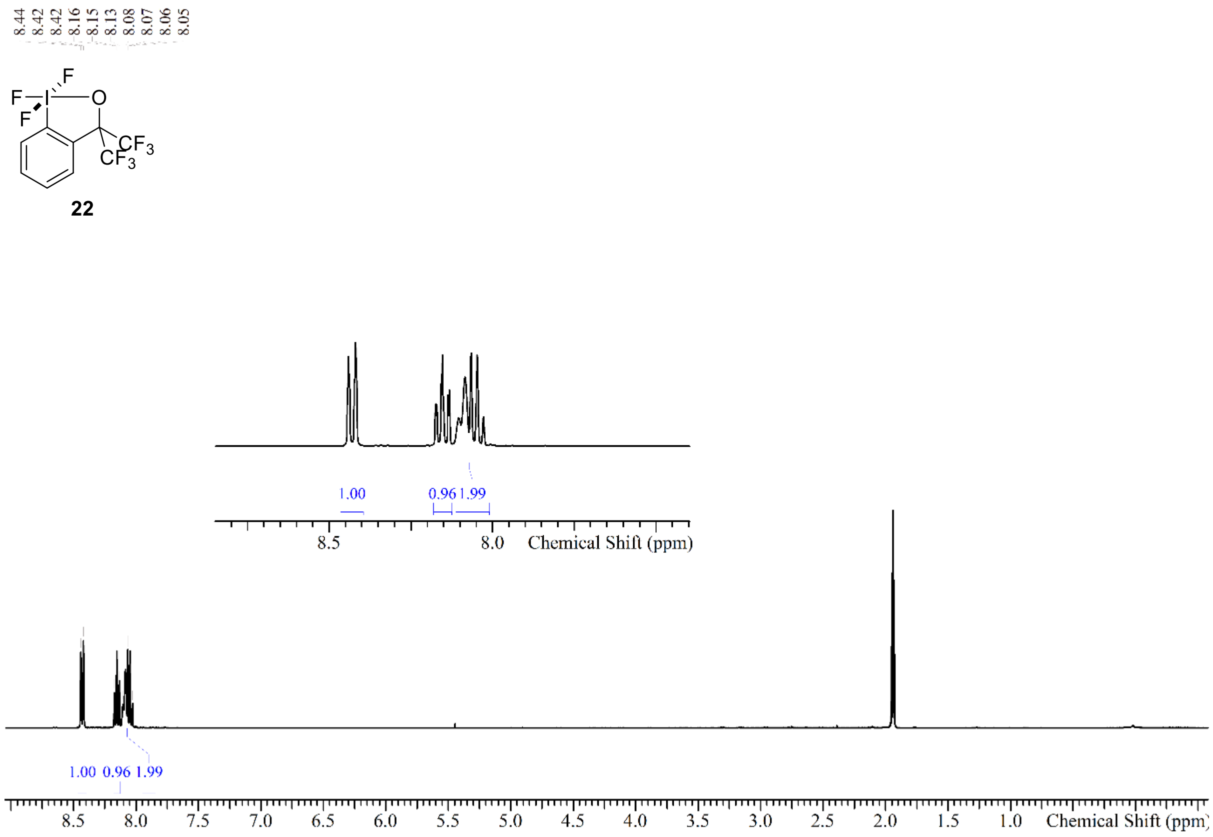

[13] C NMR spectrum of bis(trifluoromethyl)trifluoroiodane **22** – CD_3CN

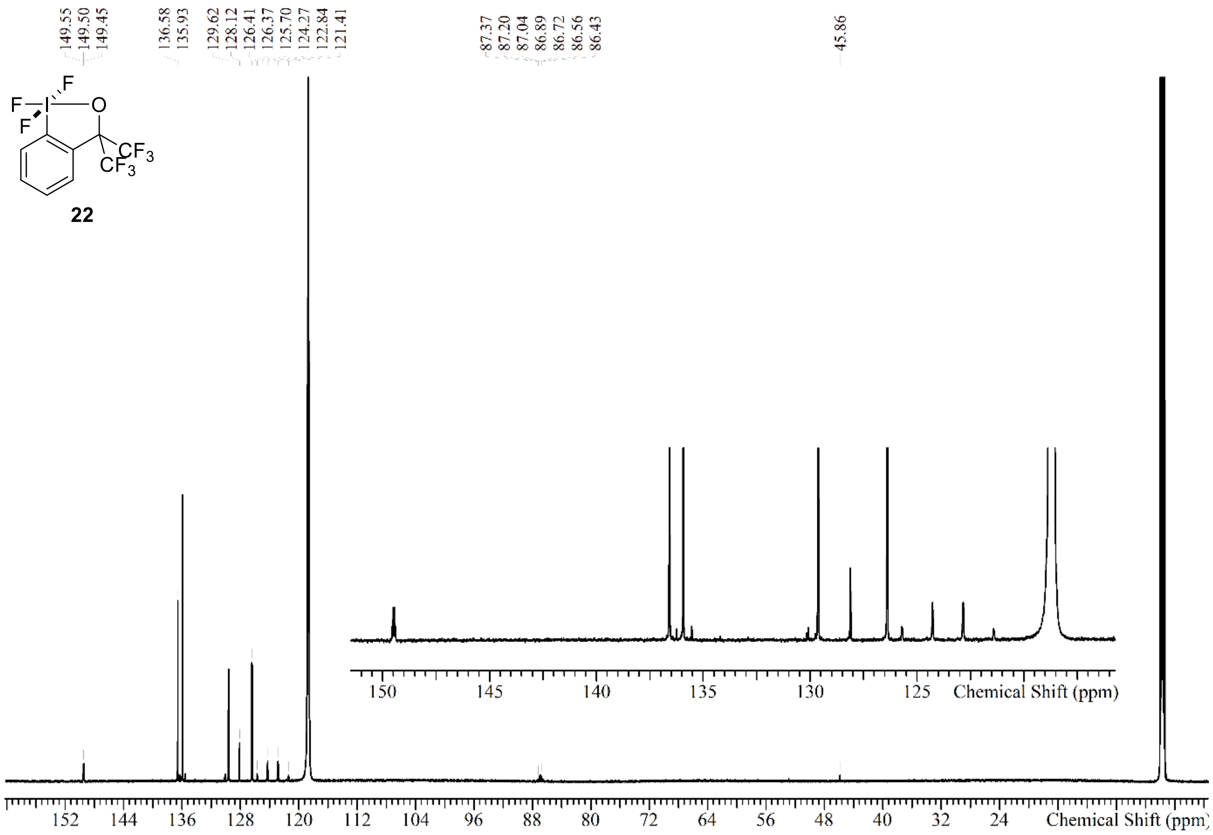

[19] F NMR spectrum of bis(trifluoromethyl)trifluoroiodane **22** – CD_3CN

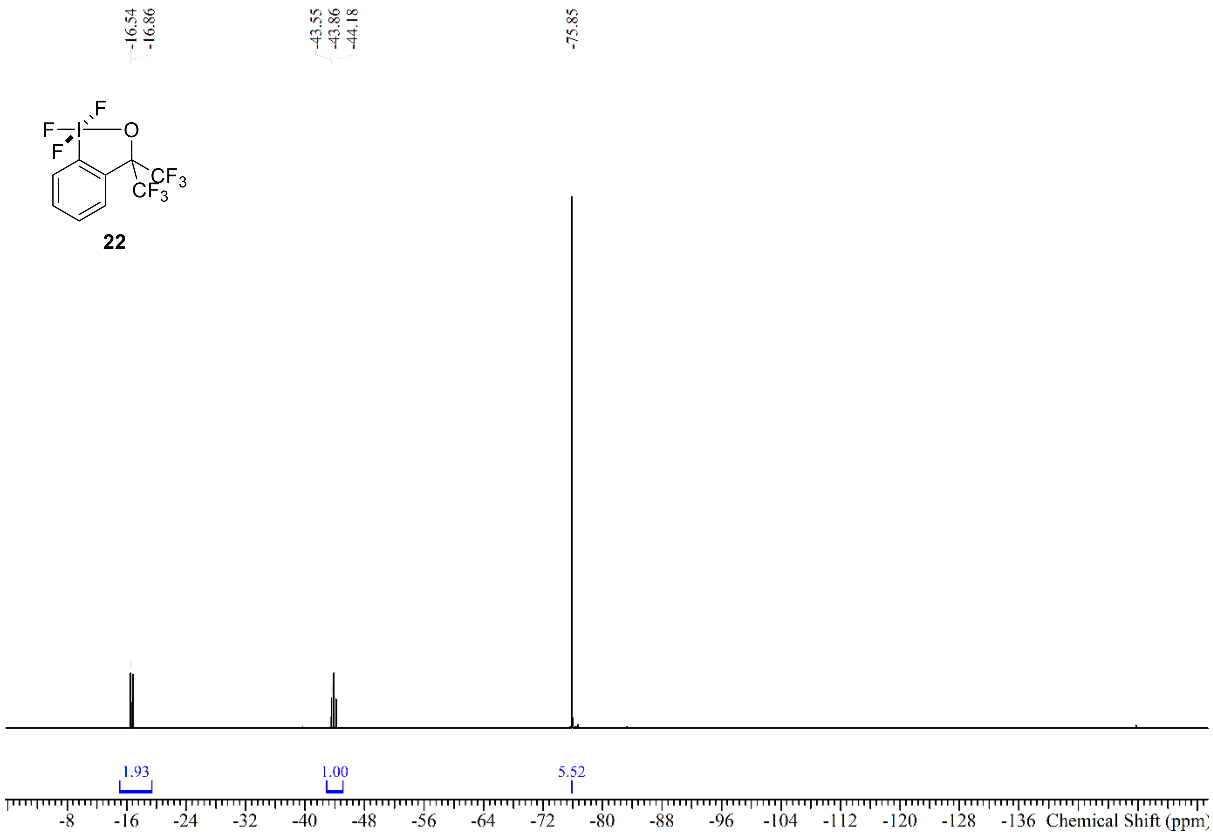

^{1}H NMR spectrum of bis(trifluoromethyl)iodosyl fluoride **S14** – CD_3CN

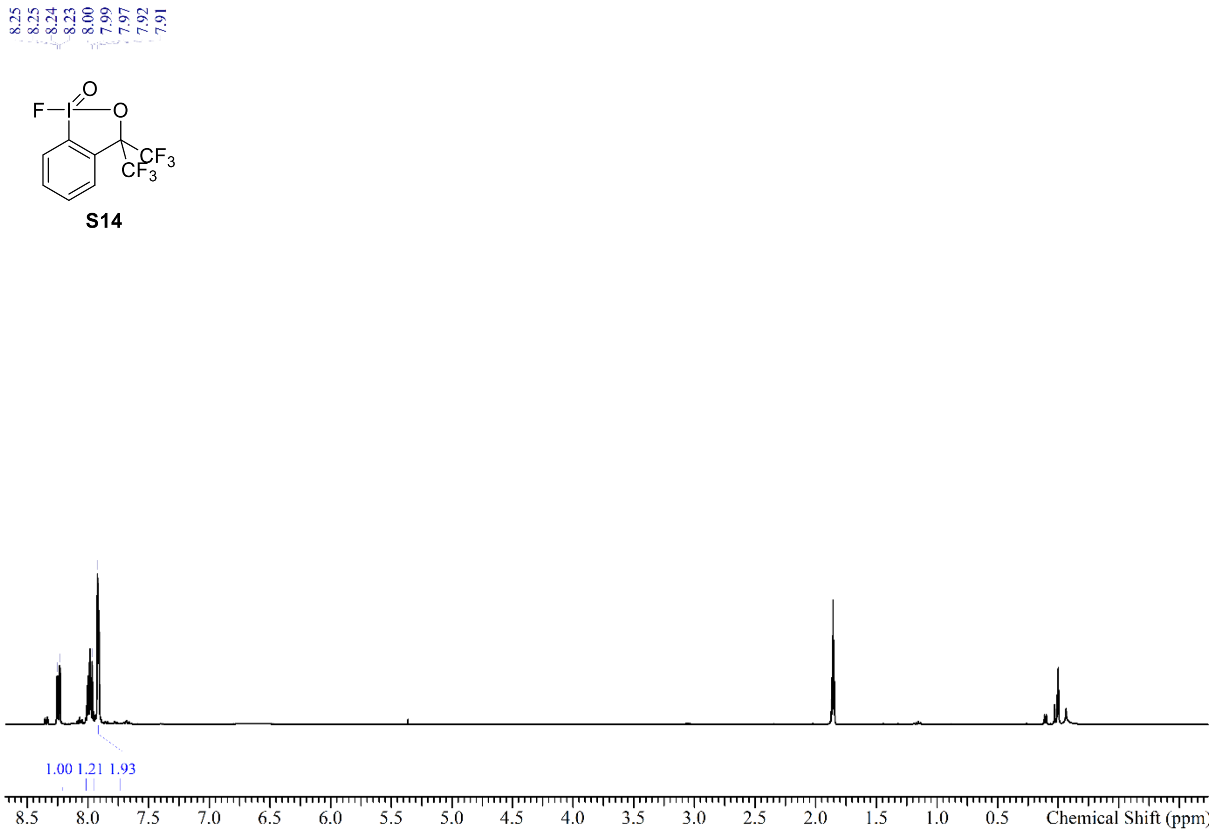

^{13}C NMR spectrum of bis(trifluoromethyl)iodosyl fluoride **S14** – CD_3CN

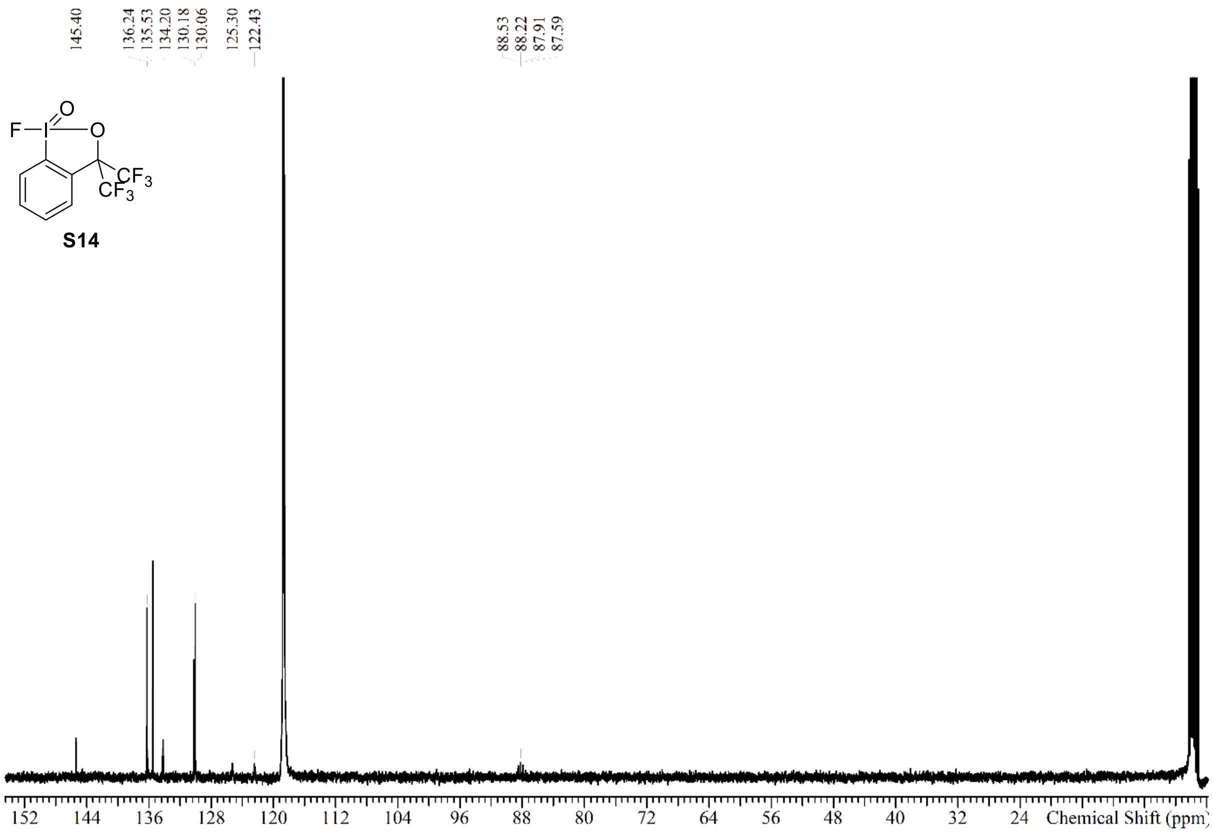

^{19}F NMR spectrum of bis(trifluoromethyl)iodosyl fluoride **S14** – CD_3CN

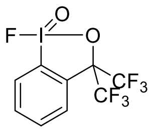

S14

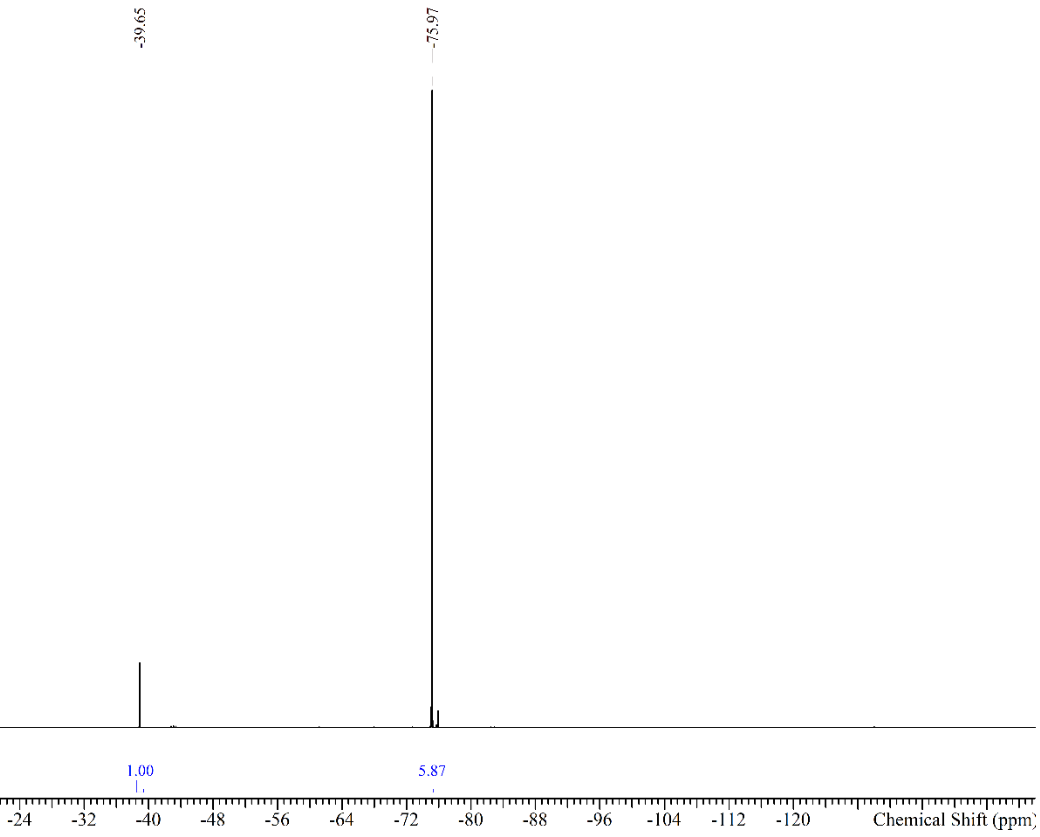

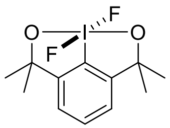

6

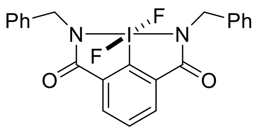

7a

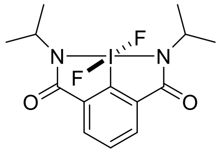

7b

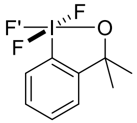

20

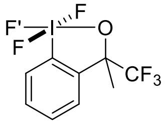

21

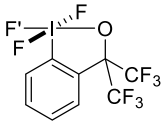

22

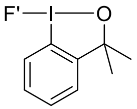

2

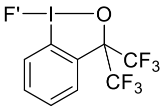

19

Table S5: Comparing properties of hypervalent iodine(V) fluorides **6**, **20-22**, hypervalent iodine (III) fluorides **2** and **19** and hypothetical iodine(V) fluorides **7a** and **7b** via DFT calculations[a]

	d(C–I) (Å)	d(I–F) (Å)	d(I–F') (Å)	d(I–O/N) (Å)	q_C	q_I	q_F	$q_{F'}$	$q_{O/N}$	θ_{C-I-F} (°)	θ_{F-I-F} (°)	$\theta_{C-I-F'}$ (°)	$\theta_{C-I-O/N}$ (°)	$\theta_{O-I-O/F'}$ /θ_{N-I-N}	$\theta_{F-I-F'}$ (°)	$\phi_{C-C-I-F}$[b] (°)
**6**	2.04	2.00[c]	–	2.03	–0.355	1.689	-0.460	–	–0.561	86.7	173.6	–	80.6	161.1	–	90.0
**20**	2.09	1.98[c]	1.99	1.99	–0.419	1.738	-0.453	–0.449	–0.556	86.0	171.8	87.0	81.8	168.8	88.6	88.8
**21**	2.09	1.98[d]	1.98	2.01	–0.415	1.753	–0.445[e]	–0.439	–0.550	86.1[f]	171.7	87.4	81.2	168.6	88.4[g]	83.6[h]
**22**	2.10	1.97[c]	1.97	2.03	–0.407	1.766	-0.438	–0.428	–0.540	86.2 °	171.4	87.8	80.5	168.5	87.8	92.1
**2**	2.10	–	2.04	2.07	–0.403	0.957	–	–0.490	–0.574	–	–	87.2	80.8	167.9	–	–
**19**	2.10	–	2.02	2.09	–0.387	1.009	–	–0.469	–0.552	–	–	87.9	79.8	167.8	–	–
**7a**	2.06	2.00	–	2.12	–0.312	1.668	-0.426	–	–0.609	88.0	175.9	–	78.3	156.6	–	88.2
**7b**	2.05	1.99[c]	–	2.12	–0.314	1.667	-0.444	–	–0.591	88.4	176.7	–	78.4	156.9	–	90.0

a Calculations performed at wB97xD/cc-pvdz, with a cc-pvdz-PP basis set used for the iodine atom. C refers to ipso carbon atom, F and F' refers to fluorine atom bound to iodine. b Dihedral angle of $C_{ortho}-C_{ipso}-I-F$ and $C_{ortho}-C_{ipso}-I-F'$. c Structure is nearly

symmetric about F‑I‑F (mirror plane). d Average between 1.99 and 1.97. e Average between 0.441 and 0.449. f Average between 85.6 and 86.6. g Average between 88.4 and 88.5. h Average between 84.8 and 82.5.

Coordinates and total energy (Hartree)

6 E = -1111.878380 au

I	0.00000200	1.33381700	0.00010500
C	0.00000500	-0.71120400	-0.00016700
C	1.23803200	-1.30152700	-0.00019200
C	-1.23802300	-1.30152900	-0.00012500
C	1.22737900	-2.69803100	-0.00026400
C	-1.22736800	-2.69803200	-0.00022000
C	0.00000700	-3.37154500	-0.00029000
H	2.16008400	-3.26487900	-0.00028100
H	-2.16007000	-3.26488500	-0.00023000
H	0.00000700	-4.46310300	-0.00035300
F	0.00017200	1.22165400	1.99359600
F	-0.00021600	1.22224800	-1.99340800
O	-1.99951200	1.00172600	0.00031800
O	1.99951200	1.00173700	-0.00015800
C	-2.44385700	-0.35625200	-0.00001400
C	-3.26111800	-0.56786300	1.27572900
C	-3.26091400	-0.56736400	-1.27597700
H	-2.62900300	-0.35978700	2.15043100
H	-4.11476500	0.12456600	1.28434800
H	-3.64061600	-1.59908800	1.34024300
H	-2.62866400	-0.35891700	-2.15049400
H	-3.64036900	-1.59857500	-1.34097200
H	-4.11457500	0.12505000	-1.28444700
C	2.44386400	-0.35625700	-0.00002700
C	3.26125000	-0.56761800	-1.27572700
C	3.26078900	-0.56758500	1.27598000
H	2.62922500	-0.35937300	-2.15045300
H	4.11490200	0.12480700	-1.28412700

H	3.64074700	-1.59883400	-1.34040600
H	2.62844200	-0.35930400	2.15046500
H	3.64025500	-1.59880100	1.34083200
H	4.11443600	0.12484300	1.28466100

20 E = -1019.188701 au

I	1.32621800	-0.19476300	0.00002700
C	-0.48026900	0.86138100	-0.00006700
C	-1.56406100	0.00417200	-0.00009600
C	-0.55758400	2.24671500	-0.00011300
C	-2.83265600	0.59259000	-0.00016400
C	-1.83201700	2.80491500	-0.00019000
C	-2.95936100	1.97862500	-0.00021400
H	-3.72469700	-0.03642600	-0.00017800
H	-1.94588000	3.88956300	-0.00023100
H	-3.95498800	2.42514100	-0.00027300
F	1.22831200	-0.09176000	1.97913600
F	1.22869800	-0.09150600	-1.97907300
O	0.08817500	-1.75253400	-0.00016700
C	-1.31985100	-1.50119600	0.00001000
C	-1.88566800	-2.12782600	-1.27476200
C	-1.88534600	-2.12753800	1.27507500
H	-1.41013300	-1.66603600	-2.15134300
H	-1.66813200	-3.20521800	-1.28057600
H	-2.97503000	-1.98773900	-1.33914300
H	-1.40954400	-1.66557500	2.15142000
H	-2.97468300	-1.98738200	1.33972500
H	-1.66785300	-3.20493800	1.28106200
F	2.24159700	1.57683800	0.00022900
H	0.34942200	2.84815100	-0.00008500

21 E = −1316.853684 au

I	1.45639500	−0.76371600	−0.00070500
C	0.50895300	1.10307200	−0.03276600
C	−0.84140800	1.00397100	0.24793400
C	1.18173900	2.28599100	−0.29767300
C	−1.57283700	2.19456000	0.27869200
C	0.42821100	3.45598100	−0.27311900
C	−0.93745000	3.40487500	0.01432700
H	−2.63979400	2.17192600	0.49665000
H	0.91146700	4.41216900	−0.47683300
H	−1.51967400	4.32727700	0.03379500
F	1.68315900	−0.44269500	1.94536900
F	1.19186400	−0.80209200	−1.95495700
O	−0.42723100	−1.36961000	0.34432900
C	−1.40745600	−0.37769400	0.56438100
C	−2.54948700	−0.71998800	−0.41205500
C	−1.87658800	−0.45786100	2.01655700
H	−1.00825200	−0.27198900	2.66207400
H	−2.65783000	0.28453100	2.22842200
H	−2.26764500	−1.46305300	2.22093300
F	3.16521400	0.19831000	−0.30589000
H	2.24950600	2.27884100	−0.50787600
F	−2.16316200	−0.60886700	−1.68303600
F	−2.98791700	−1.96646800	−0.21709900
F	−3.60481700	0.10366800	−0.23017700

22 E = −1614.508025 au

I	1.68068500	−0.81617600	−0.00003900
C	0.86029700	1.11261600	−0.00009300
C	−0.52347700	1.10880500	−0.00002500

C	1.64579800	2.25363500	-0.00018600
C	-1.17042600	2.34648500	-0.00005300
C	0.97922200	3.47644300	-0.00021200
C	-0.41547600	3.51673700	-0.00014500
H	-2.25732200	2.39221800	-0.00000200
H	1.55572700	4.40211900	-0.00028600
H	-0.92831000	4.47938200	-0.00016700
F	1.69601500	-0.66953500	1.96524900
F	1.69583900	-0.66972000	-1.96534200
O	-0.29747100	-1.29158500	0.00007200
C	-1.22065200	-0.24876300	0.00006800
C	-2.07121400	-0.40944900	-1.28791800
C	-2.07109600	-0.40933900	1.28814600
F	3.46937000	0.01940000	-0.00015700
H	2.73143700	2.17860000	-0.00023600
F	-1.29487700	-0.24609200	-2.35828000
F	-2.62824800	-1.61515300	-1.35545800
F	-3.05638200	0.50177300	-1.34958200
F	-2.62812100	-1.61503800	1.35584300
F	-3.05626100	0.50188500	1.34982300
F	-1.29466100	-0.24588700	2.35842400

2 E = -819.611165 au

I	1.49694800	-0.30472700	-0.00014300
C	-0.25805200	0.85855600	-0.00014100
C	-1.40214100	0.08008000	-0.00008500
C	-0.23758300	2.24555400	-0.00012100
C	-2.62245300	0.76421600	0.00000100
C	-1.46896900	2.89730300	-0.00007000
C	-2.65290700	2.15686500	-0.00000700

H	-3.55789500	0.20099600	0.00008000
H	-1.50197900	3.98777600	-0.00007200
H	-3.61447800	2.67283400	0.00004600
O	0.09228100	-1.82267800	-0.00096500
C	-1.27208400	-1.44582200	0.00006100
C	-1.91977900	-2.02010300	-1.26587100
C	-1.91798200	-2.01958100	1.26716700
H	-1.43506000	-1.60057200	-2.15948100
H	-1.78598900	-3.11144700	-1.27733900
H	-2.99692300	-1.79699200	-1.31187000
H	-1.43190200	-1.59978800	2.15992100
H	-2.99502400	-1.79629000	1.31469500
H	-1.78430900	-3.11093900	1.27883700
F	2.53594500	1.44564900	0.00055900
H	0.71526000	2.77310800	-0.00013400

19 E = -1414.941070 au

I	1.80772000	-1.00873700	0.00003200
C	1.08932400	0.96871200	-0.00007200
C	-0.29341400	1.05401100	-0.00011000
C	1.94421800	2.05889400	-0.00010800
C	-0.85507300	2.33365900	-0.00017500
C	1.35881700	3.32377600	-0.00019500
C	-0.02943200	3.45565000	-0.00022600
H	-1.93692900	2.44897600	-0.00018300
H	1.99541700	4.20959300	-0.00023200
H	-0.48041800	4.44876600	-0.00028400
O	-0.25583900	-1.36516200	-0.00013200
C	-1.08309100	-0.25829900	-0.00001000
C	-1.94638700	-0.34391100	-1.28586000

C	-1.94603200	-0.34385900	1.28608000
F	3.67617600	-0.25308500	-0.00042400
H	3.02246800	1.90724800	-0.00006100
F	-2.85470900	0.64390700	1.36547900
F	-1.14821700	-0.24674600	2.35705700
F	-2.60091600	-1.49952800	1.36499400
F	-2.85531000	0.64366800	-1.36487100
F	-2.60104800	-1.49970700	-1.36469700
F	-1.14892900	-0.24651000	-2.35707000

7a E = -1603.521748 au

I	0.00010200	-0.36924900	0.00009400
C	-0.00024200	1.68582000	0.00001300
C	1.11939900	2.29233400	-0.51241300
C	-1.11996800	2.29216700	0.51246300
C	1.11352800	3.68690400	-0.51249600
C	-1.11436100	3.68674000	0.51239000
C	-0.00048600	4.36395200	-0.00009300
H	1.97573400	4.22322800	-0.91027200
H	-1.97665200	4.22295100	0.91013700
H	-0.00060100	5.45494500	-0.00014300
N	1.90582900	0.06195600	-0.83000900
N	-1.90568300	0.06151600	0.83008900
C	2.21000700	1.39087600	-1.02456700
O	3.21966100	1.80571700	-1.55566900
C	-2.21021600	1.39046000	1.02473200
O	-3.21997300	1.80491400	1.55590800
F	-0.77325700	-0.29870500	-1.83916200
F	0.77346300	-0.29821600	1.83933400
C	2.82499900	-0.95276700	-1.31909000
H	3.19052000	-0.63750300	-2.30828000

H	2.25323200	−1.88450600	−1.47452100
C	−2.82484000	−0.95323600	1.31906700
H	−3.19046700	−0.63803000	2.30823500
H	−2.25313000	−1.88501400	1.47449100
C	−4.00467100	−1.22770400	0.40526600
C	−3.91155900	−1.08105200	−0.98045400
C	−5.20331400	−1.68120000	0.96219500
C	−5.00220800	−1.38815000	−1.79309900
H	−2.98792600	−0.71663800	−1.43353000
C	−6.29121000	−1.99171900	0.14987300
H	−5.29028200	−1.78459300	2.04684200
C	−6.19318300	−1.84602700	−1.23324900
H	−4.91783900	−1.26502900	−2.87450600
H	−7.22262900	−2.33986300	0.60029500
H	−7.04598300	−2.08279300	−1.87199800
C	4.00490000	−1.22737400	−0.40541700
C	3.91187300	−1.08072200	0.98031100
C	5.20343400	−1.68105800	−0.96241800
C	5.00251100	−1.38800700	1.79290500
H	2.98835000	−0.71608700	1.43343200
C	6.29131400	−1.99176000	−0.15014800
H	5.29032400	−1.78444700	−2.04706900
C	6.19337500	−1.84607100	1.23298100
H	4.91821900	−1.26488700	2.87431700
H	7.22266500	−2.34004200	−0.60060500
H	7.04617300	−2.08299200	1.87167800

7b E = −1298.766261 au

I	−0.00003200	−0.81137200	0.00083700
C	0.00002400	1.23866900	−0.00018600
C	−1.23172900	1.84240700	−0.00002600

C	1.23183200	1.84229700	−0.00029800
C	−1.22692100	3.23704700	−0.00028300
C	1.22721000	3.23692800	−0.00052900
C	0.00017200	3.91342700	−0.00049700
H	−2.17591000	3.77434400	−0.00027500
H	2.17624500	3.77413900	−0.00072800
H	0.00021900	5.00453000	−0.00068500
N	−2.07721000	−0.38714600	0.00107300
N	2.07708500	−0.38729000	0.00035600
C	−2.43756000	0.93658100	0.00018900
O	−3.57461400	1.37426700	−0.00037300
C	2.43752600	0.93638100	−0.00039700
O	3.57464000	1.37389600	−0.00093300
F	0.00032000	−0.75302700	1.99124700
F	−0.00028900	−0.75538800	−1.98970500
C	−3.06005200	−1.47980000	−0.00021800
C	−3.89372200	−1.47400200	−1.28090300
C	−3.89764900	−1.47377600	1.27789200
H	−2.45849600	−2.40875700	0.00083400
H	−3.23456300	−1.48708300	−2.16136800
H	−4.54313400	−2.36183900	−1.31282600
H	−4.52284900	−0.57459700	−1.32450500
H	−3.24120300	−1.48568700	2.16039900
H	−4.52772800	−0.57493300	1.31900200
H	−4.54630200	−2.36221900	1.30842700
C	3.06002200	−1.47989200	−0.00019200
C	3.89638700	−1.47399500	1.27870300
C	3.89481100	−1.47373500	−1.28013000
H	2.45852700	−2.40889000	0.00015000
H	3.23908300	−1.48643100	2.16056700

H	4.54529700	−2.36224000	1.30956400
H	4.52612100	−0.57495500	1.32072000
H	3.23640700	−1.48652000	−2.16116100
H	4.52401400	−0.57434300	−1.32290200
H	4.54425700	−2.36155900	−1.3117890
